# Global burden and inequalities of Hodgkin and non-Hodgkin lymphoma among children aged 0–14 years, 1990–2021: a GBD 2021 analysis with forecasts to 2035

**DOI:** 10.1016/j.jped.2026.101617

**Published:** 2026-09-22

**Authors:** Chengjun Wang, Yin Peng, Wenbin Hu

**Affiliations:** a Anhui Provincial Children's Hospital, Hefei, 230002, China; b Affiliated Hospital of the Neurology Research Institute of Anhui University of Chinese Medicine, Hefei, China

**Keywords:** Hodgkin lymphoma, Non-Hodgkin lymphoma, Global Burden of Disease, Estimated annual percentage change, Slope index of inequality, Concentration index of inequality

## Abstract

**Objective:**

Childhood Hodgkin lymphoma (HL) and non-Hodgkin lymphoma (NHL) are potentially curable, but outcomes differ across development levels. The authors assessed global burden and inequalities among children aged 0–14 years from 1990 to 2021 and projected burden to 2035.

**Methods:**

GBD 2021 estimates for 204 locations provided age-standardized incidence, mortality, and disability-adjusted life-year (DALY) rates by sex and Sociodemographic Index (SDI). Estimated annual percentage changes (EAPCs) summarized trends; the slope index of inequality (SII) and concentration index (CII) measured absolute and relative inequalities. An exploratory Country Priority Index (CPI) combined mortality and DALY rates, absolute DALYs, and adverse trajectories. Autoregressive integrated moving average (ARIMA) models generated forecasts.

**Results:**

From 1990 to 2021, incidence declined modestly. Mortality declined from 0.152 to 0.095 per 100,000 for HL and from 0.75 to 0.45 for NHL; corresponding DALY rates fell from 12.50 to 7.78 and from 63.38 to 37.83. Boys and low-SDI settings had higher burdens, and SDI was strongly inversely associated with mortality and DALYs. For NHL mortality, the SII improved from -0.91 to -0.61, whereas the CII worsened from -0.38 to -0.49. The CPI ranked Pakistan, Uganda, South Sudan, Zimbabwe, and Nigeria highest. By 2035, projected HL and NHL incidence rates were 0.20 and 0.98, mortality rates 0.08 and 0.35, and DALY rates 6.22 and 27.25 per 100,000, respectively.

**Conclusion:**

Childhood lymphoma outcomes improved globally, but relative inequalities remained concentrated in lower-SDI settings. CPI-based prioritization supports investment in diagnosis, treatment, essential medicines, supportive care, and surveillance. Forecasts represent conditional baseline trajectories.

## Introduction

Childhood Hodgkin lymphoma (HL) and non-Hodgkin lymphoma (NHL) are potentially curable when accurate diagnosis, protocol-directed treatment, essential medicines, and supportive care are consistently available. However, children in many low-income and middle-income settings still encounter delayed recognition, fragmented referral, treatment abandonment, and preventable treatment-related mortality. These failures produce large geographic differences in survival and health loss [1,2]. Joint analysis is appropriate because both diseases depend on pathology, staging, chemotherapy, supportive care, and sustained follow-up.

Observed incidence is shaped by case ascertainment. Children who never reach specialist services, lack pathological confirmation, or are omitted from registration may be absent from observed counts. Thus, low recorded incidence can coexist with high mortality and may indicate underdiagnosis or incomplete registration rather than low biological occurrence. Registry analyses show that childhood cancer incidence varies with diagnostic and health-system capacity, and international comparisons remain sensitive to coverage and data quality [3,4]. Mortality and disability-adjusted life-years (DALYs) provide complementary information on downstream care and premature health loss.

The Global Burden of Disease (GBD) 2021 study provides internally comparable estimates across locations, years, ages, and sexes [5]. A recent GBD analysis described HL among children and adolescents aged 0–19 years, but the broader age range limits direct comparison with conventional childhood surveillance [6]. The authors therefore examined HL and NHL among children aged 0–14 years from 1990 to 2021 using identical definitions. The present study’s objectives were to quantify global and national burden, assess sex and Sociodemographic Index (SDI) patterns, measure absolute and relative inequalities, identify countries where high burden and adverse trends converge, and provide conditional forecasts to 2035.

## Methods

### Study design and data sources

The authors conducted a population-based secondary analysis of publicly available GBD 2021 estimates for 204 countries and territories, 21 GBD regions, and five SDI strata from 1990 to 2021. The prespecified age range was 0–14 years, consistent with the conventional childhood stratum used in international cancer surveillance [4]. Adolescents aged 15–19 years were excluded; accordingly, the results should not be interpreted as the total lymphoma burden among all children and adolescents.

The authors extracted annual age-, sex-, location-, and cause-specific estimates and 95% uncertainty intervals (UIs) for incidence, deaths, and DALYs due to HL and NHL. GBD maps ICD-9 and ICD-10 codes to standard cause hierarchies and combines cancer-registry, vital-registration, verbal-autopsy, and other data after corrections for incompleteness and misclassification. Cause-specific mortality is estimated using the Cause of Death Ensemble model; non-fatal outcomes are synthesized in DisMod-MR 2.1 [5,7,8]. Years of life lost are combined with years lived with disability to calculate DALYs [9,10]. UIs reflect propagated estimation uncertainty. Reporting followed GATHER recommendations [11].

### Ethics

This study used only publicly available, de-identified, aggregate GBD data and involved no participant recruitment, contact, intervention, or access to identifiable records. Under applicable institutional policy, review and approval by an institutional review board or ethics committee, and individual informed consent, were therefore not required.

### Measures and statistical analysis

Primary outcomes were age-standardized incidence, mortality, and DALY rates per 100,000 population, together with absolute case, death, and DALY counts. SDI is a 0–1 composite of lag-distributed income per capita, mean educational attainment among persons aged 15 years or older, and total fertility among those younger than 25 years. The authors used SDI as a country-level development indicator, not as a measure of individual socioeconomic position. Global, regional, national, sex-specific, and SDI-stratified estimates were compared across 1990–2021.

Temporal trends were summarized using estimated annual percentage changes (EAPCs). For each rate, a log-linear model was fitted as ln(rate) = α+β × calendar year; EAPC was calculated as 100 × [exp(β)-1], with a positive value indicating an average increase. Spearman coefficients assessed monotonic associations between SDI and incidence, mortality, and DALY rates at regional and national levels. These ecological correlations describe co-variation between country development and population burden and do not estimate individual-level effects.

Development-related inequality was evaluated across the full SDI distribution rather than only by comparing extreme quintiles. The slope index of inequality (SII) was obtained from population-weighted regression on the ridit-ranked SDI distribution and represents the modelled absolute rate difference between the highest and lowest development positions. The concentration index of inequality (CII) was calculated as twice the covariance between an outcome and fractional SDI rank divided by the outcome mean. For mortality and DALYs, negative values indicate concentration at the lower-SDI end. Reporting both measures is necessary because an absolute gap can narrow while the remaining burden becomes more relatively concentrated [12,13]. Inequality estimates were calculated across GBD uncertainty draws and summarized with 95% UIs.

The authors constructed an exploratory Country Priority Index (CPI) using three domains: (1) percentile-ranked 2021 HL and NHL mortality and DALY rates, (2) absolute HL and NHL DALYs, and (3) EAPCs for mortality and DALY rates. Component percentiles were averaged within domains; domain scores were equally weighted and rescaled from 0 to 100. Ranks 1–21 were Tier 1, ranks 22–51 Tier 2, and the remainder Tier 3. Mean relative UI width across the four rate inputs was a separate imprecision flag and did not change scores. Robustness was assessed using alternative weights and leave-one-domain-out analyses. The author-defined CPI was treated as a prioritization aid, not a validated performance measure.

Autoregressive integrated moving average (ARIMA) models were fitted to annual age-standardized series for boys, girls, and both sexes. Stationarity was assessed with the KPSS test; candidate autoregressive, differencing, and moving-average orders were compared using Akaike and Bayesian information criteria. Residual autocorrelation was evaluated with the Ljung-Box test. Forecasts to 2035 were accompanied by 80% and 95% prediction intervals. ARIMA is suitable for extrapolating serial patterns but does not represent causal mechanisms or intervention scenarios [14]; projections were therefore interpreted as conditional baselines assuming that historical dynamics broadly persist.

## Results

### Global, national, sex, and development patterns

In 2021, national estimates were highly heterogeneous (Figure 1; Tables S1-S6). The highest estimated HL incidence rates occurred in Libya and Monaco (both 0.828 per 100,000), followed by Pakistan (0.740), Uganda (0.600), and Algeria (0.569). NHL incidence was highest in Estonia (20.64), Niue (14.78), Malta (14.27), Tokelau (11.71), and Iceland (7.29). Several of these locations have small child populations and wide UIs, so the extreme rates should not be interpreted as precise biological rankings.

**Fig. 1:**
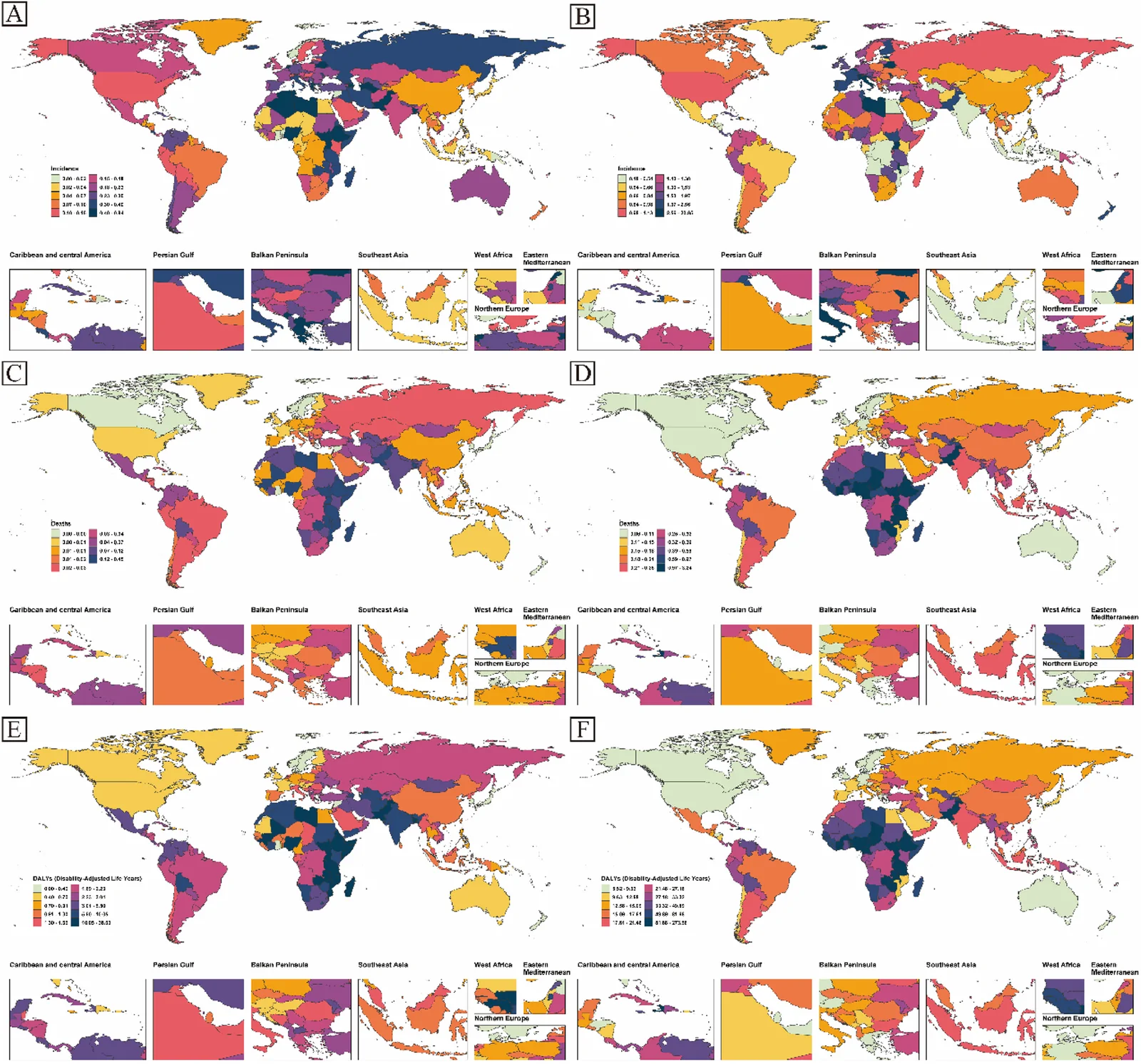
Global distribution of the burden of Hodgkin and non-Hodgkin lymphoma among children aged 0–14 years in 2021. (A) Incidence of Hodgkin lymphoma; (B) Incidence of non-Hodgkin lymphoma; (C) Mortality rate of Hodgkin lymphoma; (D) Mortality rate of non-Hodgkin lymphoma; (E) DALY rate of Hodgkin lymphoma; (F) DALY rate of non-Hodgkin lymphoma.

The highest HL mortality rates were estimated in Pakistan (0.444 per 100,000), Nigeria (0.427), Uganda (0.366), Mozambique (0.288), and Ethiopia (0.287). NHL mortality was highest in Malawi (3.21), Uganda (2.25), South Sudan (1.91), Haiti (1.50), and Eritrea (1.14). Pakistan showed a distinctive combination of high rates and large population burden: its HL DALY rate declined from 38.64 in 1990 to 36.27 in 2021, yet absolute HL DALYs increased from 19,026 to 30,989. Its NHL DALY rate increased from 97.16 to 107.27, while absolute NHL DALYs rose from 47,845 to 91,656.

Globally, HL incidence declined from 0.246 (95% UI 0.159–0.299) in 1990 to 0.213 (0.130–0.277) per 100,000 in 2021, and NHL incidence from 1.23 (1.00–1.44) to 1.03 (0.85–1.26). HL mortality declined from 0.152 (0.085–0.195) to 0.095 (0.052–0.129), and NHL mortality from 0.75 (0.57–0.89) to 0.45 (0.36–0.56). Corresponding DALY rates fell from 12.50 (6.97–16.11) to 7.78 (4.26–10.51) for HL and from 63.38 (48.09–75.72) to 37.83 (29.95–47.17) for NHL (Figure 2). Declines in mortality and DALYs were therefore much larger than declines in incidence.

**Fig. 2:**
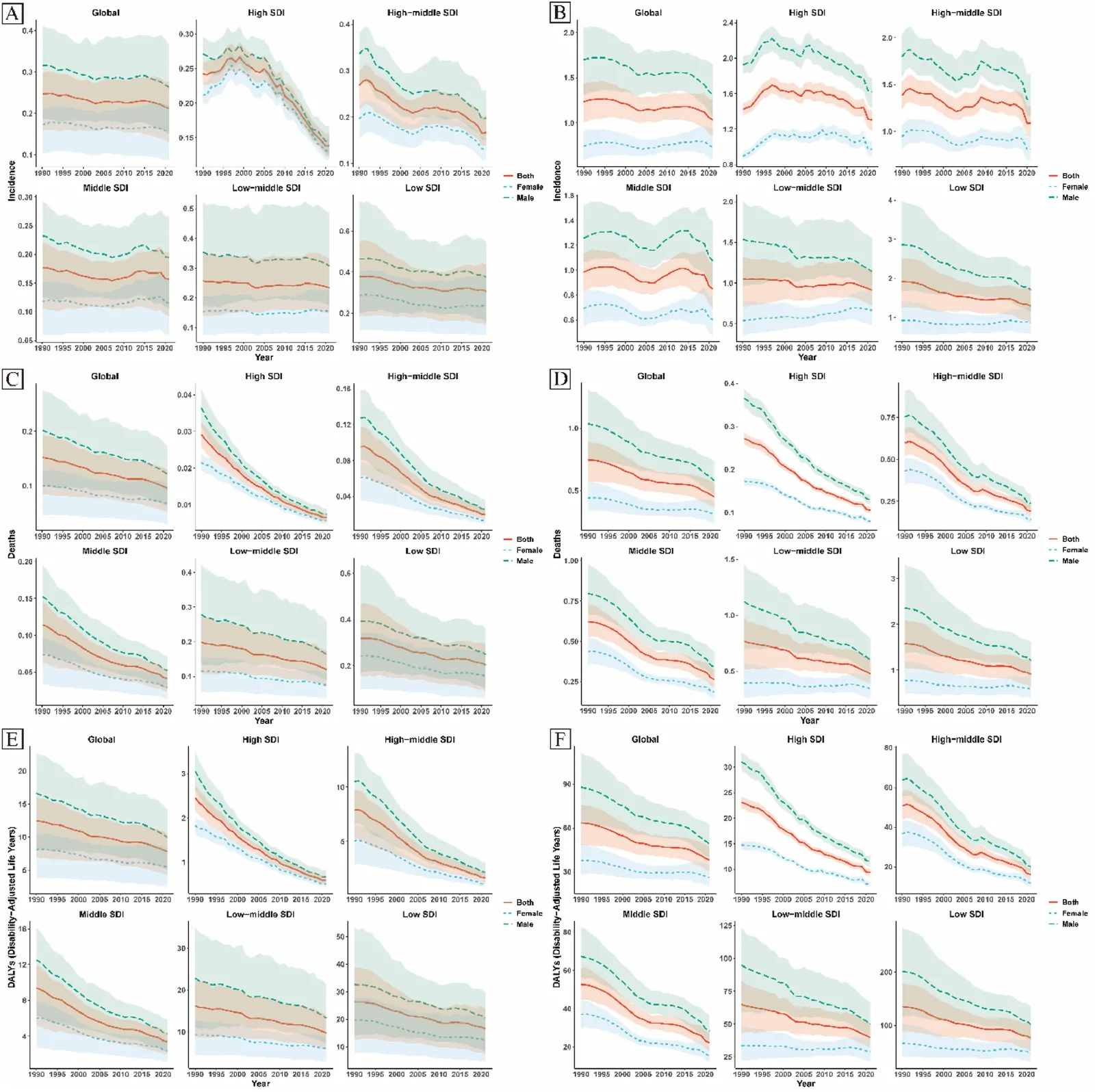
Trends in the burden of Hodgkin and non-Hodgkin lymphoma among children aged 0–14 years across five SDI quintiles worldwide, 1990–2021. (A) Incidence of Hodgkin lymphoma; (B) Incidence of non-Hodgkin lymphoma; (C) Mortality rate of Hodgkin lymphoma; (D) Mortality rate of non-Hodgkin lymphoma; (E) DALY rate of Hodgkin lymphoma; (F) DALY rate of non-Hodgkin lymphoma.

Boys consistently had a higher burden than girls. HL incidence declined from 0.316 to 0.264 among boys and from 0.173 to 0.158 among girls; in 2021, HL mortality was 0.121 versus 0.069, and the DALY rate was 9.97 versus 5.45. NHL incidence declined from 1.70 to 1.31 among boys but remained approximately 0.74 among girls. In 2021, NHL mortality was 0.58 among boys and 0.31 among girls. The sex differences were descriptive and persisted across most development strata.

Development gradients were pronounced for adverse outcomes. HL mortality declined from 0.319 to 0.202 in low-SDI settings and from 0.029 to 0.007 in high-SDI settings; the corresponding 2021 HL DALY rates were 16.52 and 0.59. NHL mortality declined from 1.58 to 0.90 in low-SDI settings and from 0.27 to 0.11 in high-SDI settings; 2021 NHL DALY rates were 76.26 and 9.39. Regional SDI was weakly associated with incidence (HL: r = 0.075, 95% CI −0.004 to 0.174; NHL: r = 0.188, 0.103–0.260) but strongly inversely associated with mortality (HL: r = −0.635, −0.697 to −0.565; NHL: r = −0.782, −0.819 to −0.740) and DALYs (HL: r = −0.623, −0.679 to −0.546; NHL: r = −0.783, −0.821 to −0.740) (Figure 3).

**Fig. 3:**
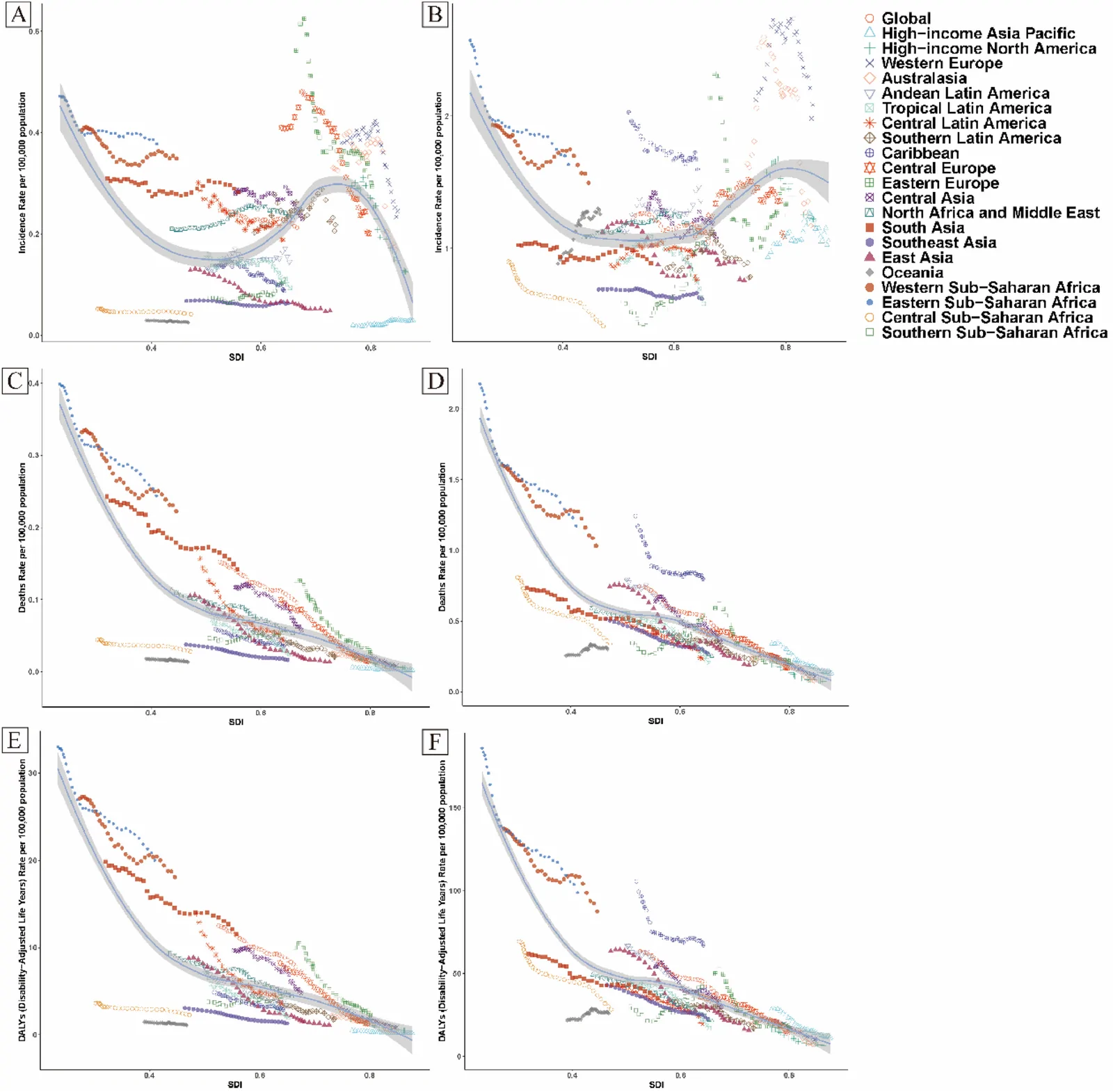
Trends in the burden of Hodgkin and non-Hodgkin lymphoma among children aged 0–14 years across 21 GBD regions worldwide, 1990–2021. (A) Incidence of Hodgkin lymphoma; (B) Incidence of non-Hodgkin lymphoma; (C) Mortality rate of Hodgkin lymphoma; (D) Mortality rate of non-Hodgkin lymphoma; (E) DALY rate of Hodgkin lymphoma; (F) DALY rate of non-Hodgkin lymphoma.

Country EAPCs were heterogeneous (Figure 4). HL incidence EAPCs ranged from −5.14% to 7.13% and NHL incidence EAPCs from −5.84% to 5.43%. HL mortality ranged from −8.06% in Norway to 4.21% in Saint Kitts and Nevis, whereas NHL mortality ranged from −5.70% in Ghana to 5.16% in Zimbabwe. DALY trends were broadly similar: most countries had declining HL DALY rates, while NHL DALY EAPCs ranged from −5.74% in Ghana to 5.22% in Zimbabwe. Rate estimates with broad UIs and trends in small populations require cautious interpretation.

**Fig. 4:**
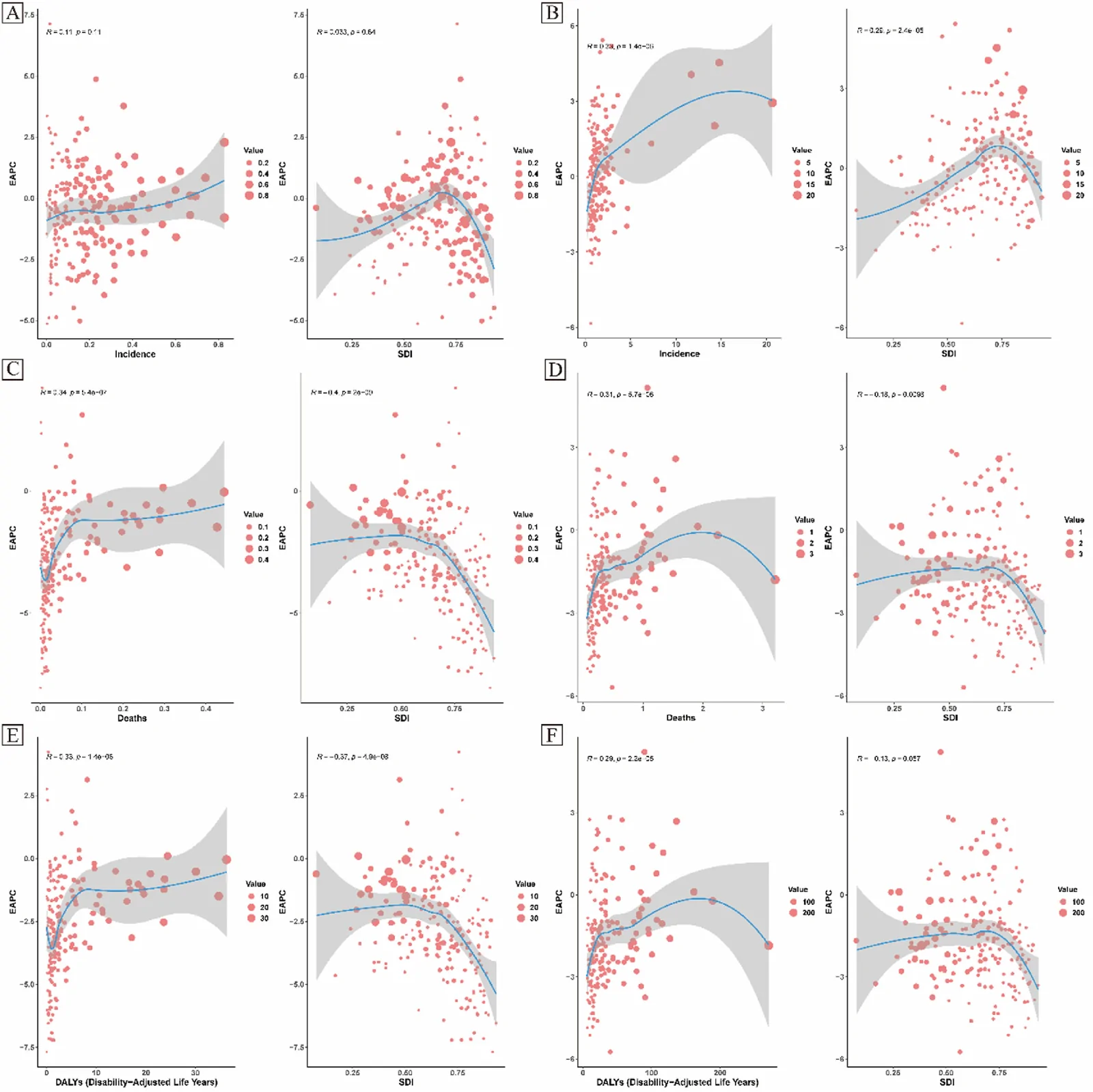
Associations between SDI and the estimated annual percentage change (EAPC) in Hodgkin and non-Hodgkin lymphoma among children aged 0–14 years. (A) Incidence of Hodgkin lymphoma; (B) Incidence of non-Hodgkin lymphoma; (C) Mortality rate of Hodgkin lymphoma; (D) Mortality rate of non-Hodgkin lymphoma; (E) DALY rate of Hodgkin lymphoma; (F) DALY rate of non-Hodgkin lymphoma.

### Inequality and country priorities

Absolute inequality in mortality and DALYs narrowed, but relative concentration in lower-SDI settings increased (Figure 5). For HL mortality, SII changed from −0.07 (95% UI −0.10 to −0.04) in 1990 to −0.06 (−0.07 to −0.05) in 2021, while CII changed from −0.43 (−0.54 to −0.27) to −0.54 (−0.63 to −0.44). For NHL mortality, SII improved from −0.91 (−1.06 to −0.77) to −0.61 (−0.69 to −0.54), whereas CII worsened from −0.38 (−0.50 to −0.25) to −0.49 (−0.59 to −0.37). DALY SIIs improved from −5.88 to −4.72 for HL and from −77.77 to −49.90 for NHL, while both CIIs became more negative. For incidence, HL SII decreased from 0.10 to 0.05 while CII shifted from −0.21 to −0.35; NHL SII changed from −0.02 to 0.60, reinforcing the distinction between occurrence, detection, and outcome inequalities.

**Fig. 5:**
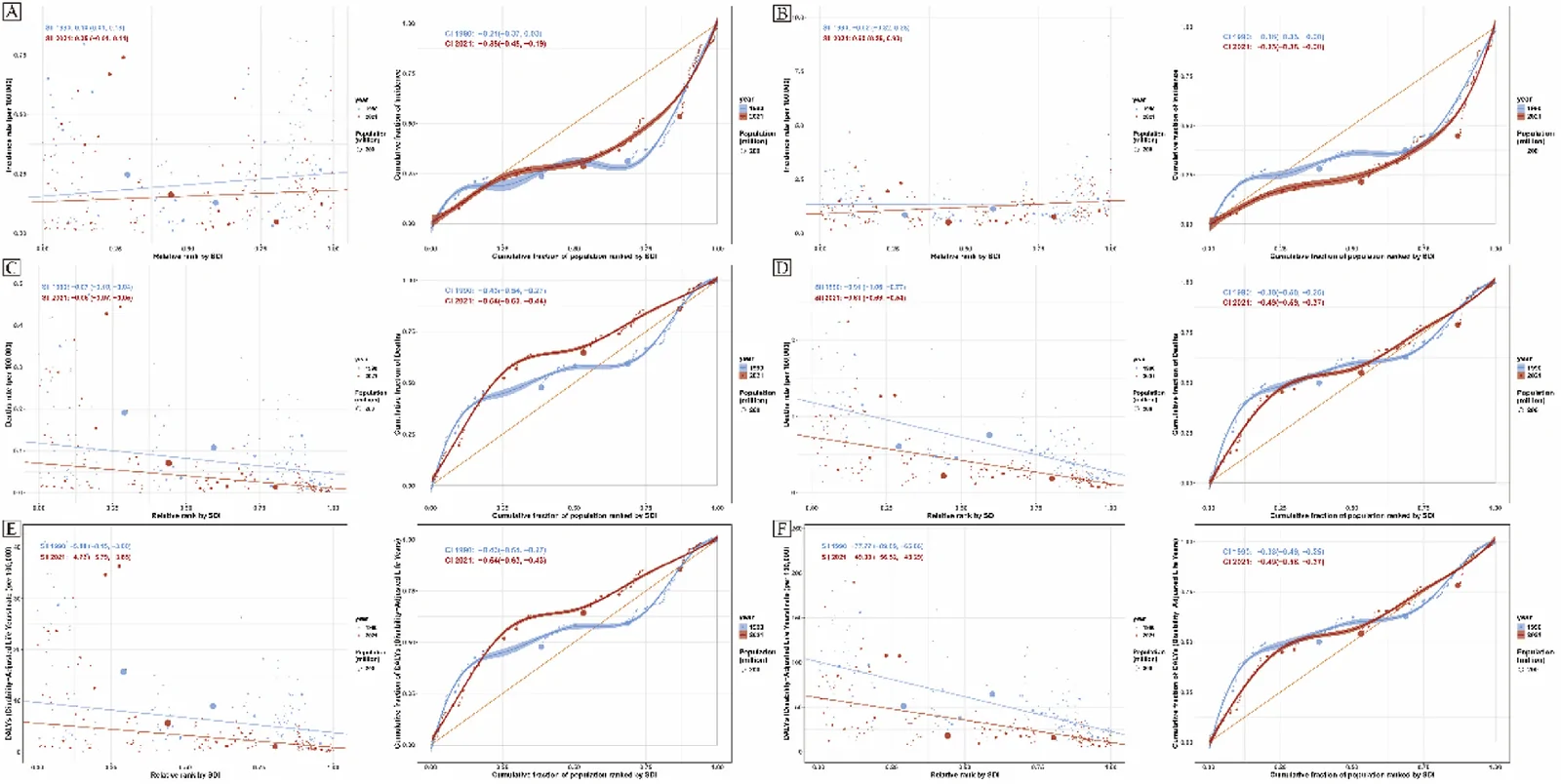
Inequality analyses of Hodgkin and non-Hodgkin lymphoma among children aged 0–14 years. (A) Incidence of Hodgkin lymphoma; (B) Incidence of non-Hodgkin lymphoma; (C) Mortality rate of Hodgkin lymphoma; (D) Mortality rate of non-Hodgkin lymphoma; (E) DALY rate of Hodgkin lymphoma; (F) DALY rate of non-Hodgkin lymphoma.

The CPI ranked Pakistan (96.67), Uganda (94.36), South Sudan (92.94), Zimbabwe (91.58), and Nigeria (91.56) highest (Table S11). Twenty-one locations were Tier 1 and 30 were Tier 2. Alternative specifications correlated strongly with the base ranking (Spearman rho = 0.873–0.989), and Tier 1 overlap ranged from 57.1% to 90.5%; agreement was lowest when population burden was omitted. Twenty-one locations had high relative UI width, indicating that prioritization should be accompanied by local verification rather than determined by rank alone.

### Forecasts

Conditional forecasts suggested continued global improvement through 2035 (Figure 6; Table S7 and S10). Projected HL incidence was 0.244 among boys, 0.158 among girls, and 0.200 for both sexes; corresponding NHL incidence was 1.18, 0.77, and 0.98 per 100,000. Projected HL mortality was 0.104 among boys, 0.057 among girls, and 0.076 overall, while NHL mortality was 0.439, 0.249, and 0.347. Projected DALY rates were 7.83, 4.52, and 6.22 for HL and 36.85, 20.79, and 27.25 for NHL. Residual diagnostics were acceptable for most models, although the NHL mortality model for girls had a marginal Ljung-Box result (p = 0.044), increasing uncertainty for that series.

**Fig. 6:**
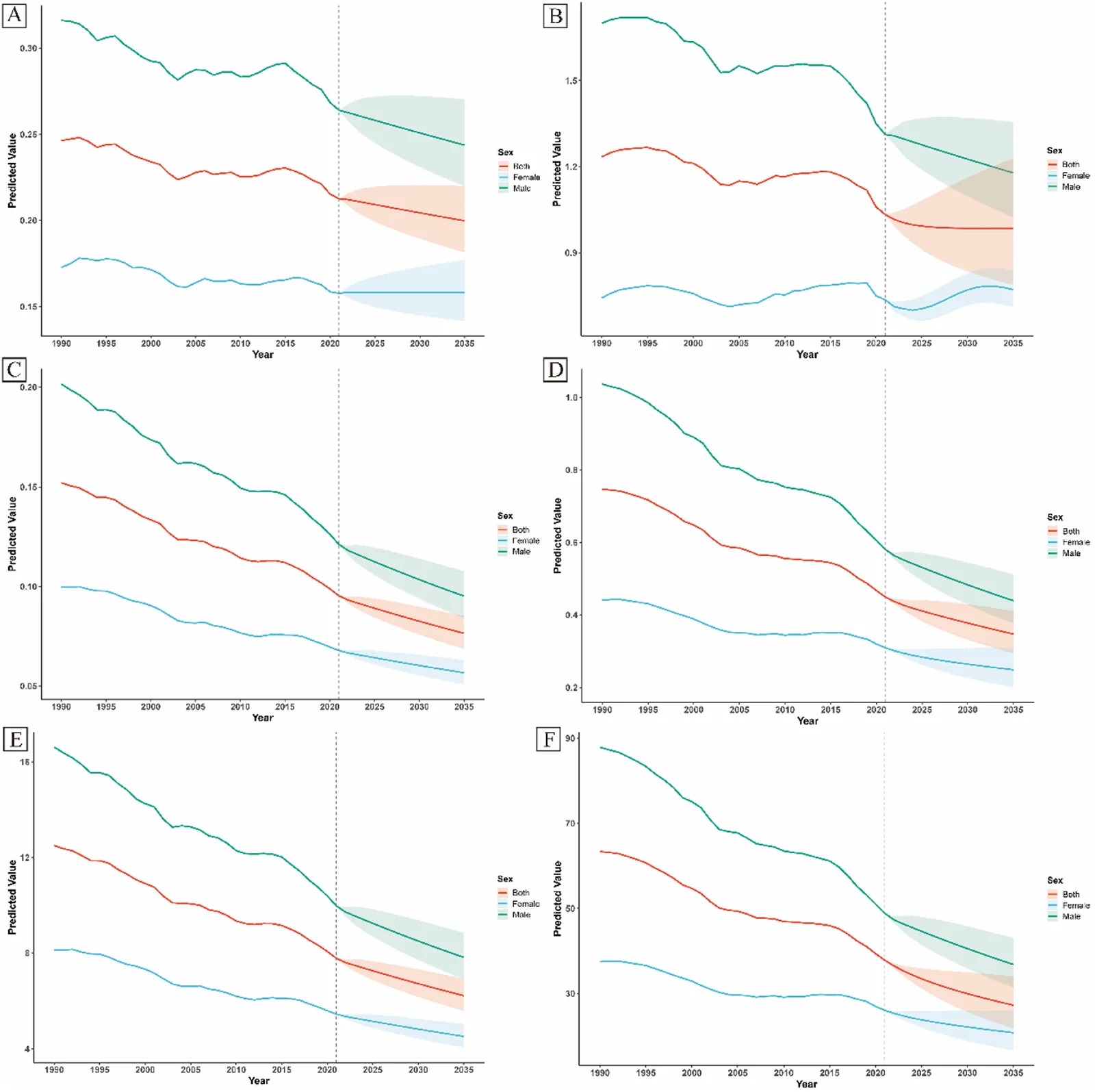
ARIMA-based forecasts of the burden of Hodgkin and non-Hodgkin lymphoma among children aged 0–14 years through 2035. (A) Incidence of Hodgkin lymphoma; (B) Incidence of non-Hodgkin lymphoma; (C) Mortality rate of Hodgkin lymphoma; (D) Mortality rate of non-Hodgkin lymphoma; (E) DALY rate of Hodgkin lymphoma; (F) DALY rate of non-Hodgkin lymphoma.

## Discussion

From 1990 to 2021, global mortality and DALY rates for childhood HL and NHL fell substantially, whereas incidence declined only modestly. Progress was uneven: boys and lower-SDI settings retained higher adverse outcomes, development gradients were much stronger for mortality and DALYs than for incidence, and several countries combined high rates with large or increasing absolute burden. Absolute gaps narrowed, but more negative CIIs showed that the remaining mortality and health loss became increasingly concentrated at the lower end of the development distribution. These paired findings are not contradictory; proportional gains can be smaller in disadvantaged settings even when absolute differences fall.

Incidence requires careful interpretation. Population-based registries remain the reference source for observed occurrence, whereas GBD models complete time series where registration and vital statistics are sparse [3–5]. Extreme estimates should trigger examination of case counts, morphology verification, death-certificate use, completeness, and coding rather than immediate conclusions about biological risk [15,16]. Discordance among outcomes is informative: low incidence paired with high mortality should prompt scrutiny of ascertainment, delayed presentation, and care access. Weak SDI-incidence associations may reflect competing influences of risk and ascertainment. Mortality and DALYs arise further downstream and are more responsive to diagnostic delay, treatment access, completion, and supportive care. These ecological associations cannot establish that national SDI causes an individual child's outcome.

The HL estimates were lower than those in the recent GBD 2021 study of ages 0–19 years, consistent with the exclusion of adolescents, among whom HL incidence is higher [6]. Comparisons with analyses based on the same GBD release demonstrate consistency of data extraction, not independent validation. The higher burden among boys accords with registry evidence that many childhood cancer subtypes are more frequently diagnosed in males [3]. Nevertheless, aggregate GBD data cannot separate biological susceptibility from sex-related differences in symptom recognition, referral, treatment access, or reporting; mechanisms should not be inferred from these descriptive patterns.

The CPI adds practical information by distinguishing countries where adverse rates, total health loss, and stalled or worsening trajectories converge. It should not be used as a league table or automatic funding formula. For Tier 1 locations, the next step is a country-specific assessment that reviews each domain alongside registry completeness, pathology and imaging capacity, referral times, treatment availability, workforce, financing, conflict or displacement, and feasibility. A country driven mainly by high rates may need diagnostic and quality-of-care review, whereas one driven by absolute DALYs may require greater service volume and decentralized referral capacity. Locations with wide UIs should prioritize data strengthening before fine-grained rank interpretation.

Policy implications span the care continuum. National childhood cancer plans can align referral, pathology, risk-adapted protocols, supportive care, and outcome monitoring with the WHO Global Initiative for Childhood Cancer [1,17]. High treatment-related mortality in lower-resource settings supports investment in infection management, blood products, nutrition, intensive-care pathways, and safe chemotherapy delivery [18]. Financial and psychosocial assistance, navigation, accommodation, and prospective tracking can reduce abandonment [19,20]. Essential medicine access requires demand forecasting, coordinated procurement, quality assurance, buffer stocks, and stock-out monitoring because availability and catastrophic expenditure remain unequal [21,22]. Registry training, standard coding, active case finding, and linkage with pathology and vital registration can improve surveillance [15,16].

Forecasts indicate what might occur if historical serial patterns broadly continue; they are not estimates of the effect of planned policies. The marginal residual result for girls' NHL mortality and the small number of annual observations reinforce this caution. Future incidence could change after registry expansion, and mortality could depart sharply from projections because of conflict, medicine shortages, new therapies, health-system investment, or another pandemic. COVID-19 disrupted diagnosis, staffing, treatment, and supply chains for childhood cancer, with disproportionate effects in lower-resource settings [23,24]. Forecasts should therefore be updated when sufficiently complete post-2021 data become available and compared with scenario-based models that explicitly represent interventions.

Strengths include coverage of 204 locations over 32 years, identical definitions for both lymphomas, uncertainty-propagated inequality analyses, and a transparent sensitivity-tested index. Limitations include greater model dependence where data are sparse; error in diagnosis, coding, and cause-of-death assignment; exclusion of ages 15–19 years; and no subtype, stage, treatment, completion, survival, or household-level socioeconomic data. SDI associations are ecological, CPI weights are normative, and ARIMA models are univariate. Country estimates and tiers should therefore be triangulated with local clinical and registry evidence.

In conclusion, childhood HL and NHL outcomes improved globally, but lower-SDI settings retained a disproportionate and increasingly concentrated share of mortality and health loss. Monitoring incidence together with mortality and DALYs, and reporting both absolute and relative inequality, provides a more defensible account of progress. Transparent CPI domains can guide verification and needs assessment, while investment in timely diagnosis, protocol-directed treatment, essential medicines, supportive care, and reliable surveillance is required to convert projected improvement into equitable outcomes.

## Authors’ contributions

Chengjun Wang and Yin Peng were responsible for the conceptualization and methodology of the research. Wenbin Hu contributed to both aspects of the study. All authors were involved in (1) the conception and design of the study, or acquisition of data, or analysis and interpretation of data, (2) drafting the article or revising it critically for important intellectual content, and (3) final approval of the version to be submitted. Hao-nan Shi accessed and verified the underlying data. The authors declare that this manuscript, including related data, figures, and tables, has not been previously published and is not under consideration elsewhere.

## Data availability

GBD study 2021 data resources were available online from the Global Health Data Exchange (GHDx) query tool (http://ghdx.healthdata.org/gbd-results-tool).

## Conflicts of interest

The authors declare no conflicts of interest.

## Appendix Supplementary materials

Image, application 1

## Footnotes

Supplementary material associated with this article can be found in the online version at doi:10.1016/j.jped.2026.101617.

## References

[1] Ortiz R., Vásquez L., Giri B., Kapambwe S., Dille I., Mahmoud L. (2023). Developing and sustaining high-quality care for children with cancer: the WHO Global Initiative for Childhood Cancer. Rev Panam Salud Publica.

[2] Smith E.R., Cotache-Condor C., Leraas H., Truche P., Ward Z.J., Stefan C. (2024). Towards attainment of the 2030 goal for childhood cancer survival for the World Health Organization Global Initiative for Childhood Cancer: an ecological, cross-sectional study. PLOS Glob Public Health.

[3] Wang M., Bi Y., Fan Y., Fu X., Jin Y. (2025). Global incidence of childhood cancer by subtype in 2022: a population-based registry study. EClin Med.

[4] Steliarova-Foucher E., Colombet M., Ries L.A., Moreno F., Dolya A., Bray F. (2017). International incidence of childhood cancer, 2001-10: a population-based registry study. Lancet Oncol.

[5] GBD 2021 Diseases and Injuries Collaborators (2024). Global incidence, prevalence, years lived with disability (YLDs), disability-adjusted life-years (DALYs), and healthy life expectancy (HALE) for 371 diseases and injuries in 204 countries and territories and 811 subnational locations, 1990-2021: a systematic analysis for the Global Burden of Disease Study 2021. Lancet..

[6] Chen J., Wang Y., Guo J., Song Y., Zhu J., Ma J. (2025). Global burden of Hodgkin lymphoma among children and adolescents: a population-based study using GBD 2021. Front Pediatr.

[7] Murray CJ. (2022). The Global Burden of Disease Study at 30 years. Nat Med.

[8] Foreman K.J., Lozano R., Lopez A.D., Murray CJ. (2012). Modeling causes of death: an integrated approach using CODEm. Popul Health Metr.

[9] Salomon J.A., Vos T., Hogan D.R., Gagnon M., Naghavi M., Mokdad A. (2012). Common values in assessing health outcomes from disease and injury: disability weights measurement study for the Global Burden of Disease Study 2010. Lancet.

[10] GBD 2021 Causes of Death Collaborators (2024). Global burden of 288 causes of death and life expectancy decomposition in 204 countries and territories and 811 subnational locations, 1990-2021: a systematic analysis for the Global Burden of Disease Study 2021. Lancet.

[11] Stevens G.A., Alkema L., Black R.E., Boerma J.T., Collins G.S., Ezzati M. (2016). Guidelines for accurate and transparent health estimates reporting: the GATHER statement. Lancet.

[12] Mackenbach J.P., Kunst AE. (1997). Measuring the magnitude of socio-economic inequalities in health: an overview of available measures illustrated with two examples from Europe. Soc Sci Med.

[13] Wagstaff A., Paci P., van Doorslaer E. (1991). On the measurement of inequalities in health. Soc Sci Med.

[14] Jian Y., Zhu D., Zhou D., Li N., Du H., Dong X. (2022). ARIMA model for predicting chronic kidney disease and estimating its economic burden in China. BMC Public Health.

[15] Moreira D.C., Znaor A., Santana V.M., Dolya A., Fox Irwin L., Bhakta N. (2024). Expanding the global capacity for childhood cancer registration: the ChildGICR Masterclass. JCO Glob Oncol.

[16] Bray F., Parkin DM. (2009). Evaluation of data quality in the cancer registry: principles and methods. Part I: comparability, validity and timeliness. Eur J Cancer.

[17] Lam C.G., Vasquez L., Loggetto P., Fuentes-Alabi S., Gonzalez Ruiz A., Benitez Majano S. (2023). Partnering to implement the Global Initiative for Childhood Cancer in the Americas: prioritizing systems strengthening. Rev Panam Salud Publica.

[18] Ehrlich B.S., McNeil M.J., Pham L.T., Chen Y., Rivera J., Acuna C. (2023). Treatment-related mortality in children with cancer in low-income and middle-income countries: a systematic review and meta-analysis. Lancet Oncol.

[19] Jatia S., Prasad M., Paradkar A., Bhatia A., Narula G., Chinnaswamy G. (2019). Holistic support coupled with prospective tracking reduces abandonment in childhood cancers: a report from India. Pediatr Blood Cancer.

[20] Chagaluka G., Afungchwi G.M., Landman L., Njuguna F., Hesseling P., Tchintseme F. (2021). Treatment abandonment: a report from the collaborative African network for childhood cancer care and research-CANCaRe Africa. Pediatr Blood Cancer.

[21] Denburg A.E., Fundytus A., Khan M.S., Howard S.C., Antillon-Klussmann F., Sengar M. (2022). Defining essential childhood cancer medicines to inform prioritization and access: results from an international, cross-sectional survey. JCO Glob Oncol.

[22] Seneviwickrama M., Denagamagei S.S., Jayakody S., Gunasekera S., Liyanage G. (2025). Availability and accessibility of cytotoxic medicines in the WHO model list of essential medicines for childhood cancer in low and lower-middle- income countries: a systematic review. BMC Cancer.

[23] Graetz D., Agulnik A., Ranadive R., Vedaraju Y., Chen Y., Chantada G. (2021). Global effect of the COVID-19 pandemic on paediatric cancer care: a cross-sectional study. Lancet Child Adolesc Health.

[24] Majeed A., Wright T., Guo B., Arora R.S., Lam C.G., Martiniuk AL. (2022). The global impact of COVID-19 on childhood cancer outcomes and care delivery - A systematic review. Front Oncol.

