# Supplementary material for: Global burden and inequalities of Hodgkin and non-Hodgkin lymphoma among children aged 0–14 years, 1990–2021: a GBD 2021 analysis with forecasts to 2035
Source: J Pediatr (Rio J). 2026 Sep 22;103(1):101617. doi: 10.1016/j.jped.2026.101617 (PMC13631578; doi:10.1016/j.jped.2026.101617)
Supplement: Supplementary file 1 [file mmc1.docx]

**JPED-D-26-00079_Supplementary Material**

**Table S1** Incidence of Hodgkin lymphoma in Children Between 1990 and 2021 at 204 Countries and Territories.

| Location | Rate per 100000 (95% UI) | | | | |
| --- | --- | --- | --- | --- | --- |
|  | 1990 | | 2021 | | EAPC^a^ |
|  | Cases | Rates | Cases | Rates |  |
| Global | 4285.13(2762.68,5204.17) | 0.25(0.16,0.30) | 4276.40(2622.82,5567.47) | 0.21(0.13,0.28) | -0.38(-0.45,-0.30) |
| 5 SDI | | | | | |
| High SDI | 449.73(423.09,482.21) | 0.24(0.23,0.26) | 239.06(211.26,270.63) | 0.14(0.12,0.16) | -1.93(-2.33,-1.54) |
| High-middle SDI | 734.39(596.79,821.62) | 0.27(0.22,0.30) | 383.81(318.29,468.59) | 0.17(0.14,0.20) | -1.29(-1.50,-1.09) |
| Middle SDI | 1020.91(602.05,1266.24) | 0.18(0.10,0.22) | 888.12(521.98,1152.22) | 0.16(0.09,0.20) | -0.17(-0.32,-0.02) |
| Low-middle SDI | 1211.66(656.91,1652.09) | 0.26(0.14,0.35) | 1355.41(859.69,1911.60) | 0.23(0.15,0.33) | -0.19(-0.27,-0.11) |
| Low SDI | 865.06(425.26,1255.71) | 0.38(0.19,0.55) | 1407.78(663.27,2017.60) | 0.31(0.14,0.44) | -0.71(-0.84,-0.59) |
| 21 GBD Region | | | | | |
| Andean Latin America | 20.11(13.68,27.07) | 0.14(0.09,0.18) | 26.29(17.67,41.02) | 0.15(0.10,0.23) | 0.53(0.30,0.77) |
| Australasia | 15.15(11.26,20.61) | 0.33(0.25,0.45) | 9.70(6.69,13.75) | 0.17(0.12,0.24) | -1.95(-2.78,-1.11) |
| Caribbean | 15.74(12.17,19.63) | 0.14(0.11,0.17) | 10.11(7.07,13.24) | 0.09(0.06,0.12) | -1.51(-1.70,-1.32) |
| Central Asia | 69.99(55.93,83.86) | 0.28(0.22,0.34) | 63.38(50.43,80.24) | 0.23(0.18,0.29) | -0.48(-0.65,-0.32) |
| Central Europe | 120.67(104.94,137.89) | 0.41(0.36,0.47) | 35.58(30.06,43.68) | 0.20(0.17,0.25) | -2.56(-3.03,-2.09) |
| Central Latin America | 193.64(180.94,207.06) | 0.30(0.28,0.32) | 119.74(103.03,140.35) | 0.19(0.16,0.22) | -1.11(-1.29,-0.94) |
| Central Sub-Saharan Africa | 13.25(7.31,24.37) | 0.05(0.03,0.10) | 24.35(15.57,39.87) | 0.04(0.03,0.07) | -0.54(-0.66,-0.42) |
| East Asia | 422.93(160.85,612.71) | 0.13(0.05,0.19) | 129.91(82.33,207.78) | 0.05(0.03,0.08) | -3.44(-3.70,-3.18) |
| Eastern Europe | 289.53(271.82,309.61) | 0.56(0.53,0.60) | 104.15(95.41,114.64) | 0.29(0.27,0.32) | -2.02(-2.34,-1.70) |
| Eastern Sub-Saharan Africa | 427.17(269.81,617.95) | 0.47(0.30,0.68) | 673.94(353.10,1079.50) | 0.38(0.20,0.60) | -0.67(-0.79,-0.54) |
| High-income Asia Pacific | 7.02(5.84,8.75) | 0.02(0.02,0.02) | 6.60(5.19,8.31) | 0.03(0.02,0.04) | 1.85(1.50,2.20) |
| High-income North America | 182.49(171.51,195.58) | 0.30(0.28,0.32) | 82.36(75.64,88.91) | 0.13(0.12,0.14) | -3.15(-3.46,-2.83) |
| North Africa and Middle East | 295.56(175.43,399.21) | 0.21(0.12,0.28) | 426.22(247.02,581.43) | 0.23(0.13,0.32) | 0.65(0.47,0.83) |
| Oceania | 0.79(0.40,1.18) | 0.03(0.01,0.04) | 1.27(0.49,2.03) | 0.02(0.01,0.04) | -0.39(-0.48,-0.30) |
| South Asia | 1341.09(678.22,1898.42) | 0.31(0.16,0.44) | 1432.19(913.63,2143.07) | 0.28(0.18,0.42) | -0.19(-0.33,-0.05) |
| Southeast Asia | 116.47(74.05,169.86) | 0.07(0.04,0.10) | 109.04(72.75,175.34) | 0.06(0.04,0.10) | -0.44(-0.61,-0.27) |
| Southern Latin America | 30.23(21.97,40.48) | 0.20(0.15,0.27) | 31.62(22.49,43.85) | 0.22(0.16,0.30) | 0.36(0.02,0.71) |
| Southern Sub-Saharan Africa | 15.23(9.85,21.73) | 0.07(0.05,0.11) | 26.49(15.68,36.63) | 0.11(0.07,0.15) | 1.26(0.97,1.56) |
| Tropical Latin America | 82.08(71.36,93.08) | 0.15(0.13,0.17) | 47.35(39.56,55.74) | 0.09(0.08,0.11) | -0.98(-1.33,-0.62) |
| Western Europe | 269.91(244.51,298.27) | 0.38(0.34,0.42) | 166.72(143.96,195.38) | 0.24(0.21,0.29) | -1.35(-1.80,-0.89) |
| Western Sub-Saharan Africa | 356.10(71.10,614.97) | 0.41(0.08,0.70) | 749.42(162.44,1366.80) | 0.35(0.08,0.64) | -0.59(-0.75,-0.42) |
| 204 countries and territories | | | | | |
| Afghanistan | 8.26(4.21,16.02) | 0.19(0.10,0.37) | 25.63(15.33,41.41) | 0.18(0.11,0.29) | 0.45(0.09,0.82) |
| Albania | 3.43(1.32,5.56) | 0.31(0.12,0.50) | 1.30(0.64,2.34) | 0.29(0.14,0.53) | 1.03(-0.00,2.07) |
| Algeria | 65.34(26.00,109.42) | 0.61(0.24,1.02) | 75.65(38.72,130.28) | 0.57(0.29,0.98) | -0.25(-0.39,-0.11) |
| American Samoa | 0.00(0.00,0.00) | 0.00(0.00,0.00) | 0.00(0.00,0.00) | 0.00(0.00,0.01) | 3.37(2.72,4.03) |
| Andorra | 0.03(0.01,0.04) | 0.27(0.16,0.46) | 0.02(0.01,0.05) | 0.24(0.13,0.45) | -0.33(-0.45,-0.20) |
| Angola | 2.99(1.35,6.12) | 0.06(0.03,0.13) | 6.31(3.58,10.80) | 0.04(0.02,0.07) | -1.31(-1.43,-1.20) |
| Antigua and Barbuda | 0.00(0.00,0.01) | 0.02(0.01,0.03) | 0.00(0.00,0.01) | 0.02(0.02,0.03) | -0.46(-1.78,0.89) |
| Argentina | 26.11(18.26,35.73) | 0.26(0.18,0.35) | 21.84(14.78,31.72) | 0.21(0.15,0.31) | -0.52(-0.85,-0.20) |
| Armenia | 2.53(1.54,3.34) | 0.24(0.15,0.32) | 1.31(0.96,1.81) | 0.22(0.16,0.31) | 0.45(0.12,0.78) |
| Australia | 14.59(10.68,19.97) | 0.39(0.28,0.53) | 9.01(6.00,13.05) | 0.19(0.13,0.27) | -2.05(-2.86,-1.25) |
| Austria | 4.66(3.45,6.41) | 0.35(0.26,0.48) | 2.16(1.39,3.26) | 0.17(0.11,0.25) | -1.40(-2.05,-0.74) |
| Azerbaijan | 9.24(4.92,15.69) | 0.38(0.20,0.65) | 12.67(6.30,22.81) | 0.54(0.27,0.97) | 0.75(0.49,1.01) |
| Bahamas | 0.03(0.03,0.04) | 0.04(0.03,0.05) | 0.03(0.02,0.04) | 0.04(0.03,0.05) | -0.59(-1.47,0.31) |
| Bahrain | 0.36(0.21,0.59) | 0.22(0.13,0.36) | 0.98(0.56,1.64) | 0.33(0.19,0.55) | 1.73(1.35,2.11) |
| Bangladesh | 212.53(111.54,391.90) | 0.43(0.23,0.80) | 186.56(98.37,392.80) | 0.41(0.21,0.86) | -0.00(-0.21,0.21) |
| Barbados | 0.05(0.04,0.07) | 0.08(0.06,0.11) | 0.03(0.02,0.05) | 0.07(0.04,0.10) | 0.36(-0.26,0.98) |
| Belarus | 13.40(8.80,20.37) | 0.56(0.37,0.85) | 6.38(3.99,9.78) | 0.40(0.25,0.62) | -0.89(-1.19,-0.59) |
| Belgium | 5.91(3.78,8.73) | 0.33(0.21,0.48) | 4.38(2.73,6.65) | 0.23(0.14,0.35) | -1.53(-2.28,-0.78) |
| Belize | 0.09(0.08,0.11) | 0.11(0.09,0.14) | 0.07(0.05,0.09) | 0.06(0.04,0.07) | -1.64(-1.90,-1.37) |
| Benin | 1.05(0.49,1.78) | 0.04(0.02,0.07) | 1.16(0.53,2.51) | 0.02(0.01,0.04) | -3.26(-3.53,-2.98) |
| Bermuda | 0.01(0.01,0.02) | 0.12(0.09,0.16) | 0.02(0.01,0.02) | 0.18(0.13,0.25) | 1.61(1.05,2.17) |
| Bhutan | 0.67(0.26,1.11) | 0.26(0.10,0.42) | 0.51(0.23,1.17) | 0.27(0.12,0.62) | -0.01(-0.29,0.27) |
| Bolivia (Plurinational State of) | 6.89(3.50,11.49) | 0.26(0.13,0.43) | 5.25(2.61,10.05) | 0.15(0.07,0.29) | -1.86(-1.95,-1.78) |
| Bosnia and Herzegovina | 2.61(1.46,3.95) | 0.24(0.13,0.36) | 1.07(0.60,2.06) | 0.22(0.12,0.42) | -0.11(-0.41,0.19) |
| Botswana | 0.46(0.27,0.79) | 0.08(0.05,0.13) | 0.58(0.32,1.04) | 0.08(0.05,0.15) | 0.36(0.21,0.52) |
| Brazil | 80.11(69.33,91.40) | 0.15(0.13,0.18) | 44.69(37.31,52.77) | 0.09(0.08,0.11) | -1.03(-1.41,-0.65) |
| Brunei Darussalam | 0.03(0.01,0.05) | 0.03(0.02,0.05) | 0.03(0.02,0.04) | 0.03(0.02,0.05) | 1.26(0.68,1.86) |
| Bulgaria | 10.08(7.38,13.37) | 0.58(0.43,0.77) | 2.20(1.55,3.15) | 0.23(0.16,0.32) | -3.42(-3.72,-3.12) |
| Burkina Faso | 2.06(0.93,3.88) | 0.04(0.02,0.08) | 2.15(0.91,4.70) | 0.02(0.01,0.05) | -2.75(-3.06,-2.43) |
| Burundi | 12.71(6.99,19.34) | 0.49(0.27,0.74) | 18.90(7.70,34.82) | 0.32(0.13,0.59) | -0.90(-1.09,-0.70) |
| Cabo Verde | 0.00(0.00,0.01) | 0.00(0.00,0.00) | 0.00(0.00,0.01) | 0.00(0.00,0.00) | -0.29(-1.00,0.42) |
| Cambodia | 2.88(1.61,5.38) | 0.06(0.03,0.12) | 2.18(0.88,5.20) | 0.04(0.02,0.10) | -1.46(-1.65,-1.27) |
| Cameroon | 2.01(0.97,3.51) | 0.04(0.02,0.07) | 2.62(1.25,5.17) | 0.02(0.01,0.04) | -3.12(-3.43,-2.81) |
| Canada | 33.26(23.83,43.81) | 0.58(0.41,0.76) | 9.60(6.54,13.05) | 0.16(0.11,0.21) | -5.02(-5.53,-4.51) |
| Central African Republic | 0.81(0.38,1.58) | 0.07(0.03,0.13) | 1.26(0.63,2.11) | 0.06(0.03,0.09) | -0.55(-0.69,-0.42) |
| Chad | 1.22(0.55,2.13) | 0.04(0.02,0.07) | 2.03(0.95,4.25) | 0.02(0.01,0.05) | -2.34(-2.61,-2.07) |
| Chile | 2.57(1.85,3.55) | 0.06(0.05,0.09) | 8.41(5.74,11.96) | 0.23(0.16,0.33) | 4.87(3.62,6.14) |
| China | 411.73(151.50,597.16) | 0.13(0.05,0.19) | 124.73(78.68,198.42) | 0.05(0.03,0.08) | -3.53(-3.80,-3.25) |
| Colombia | 30.29(23.95,37.77) | 0.26(0.21,0.32) | 30.29(21.92,41.17) | 0.29(0.21,0.39) | 0.51(0.26,0.76) |
| Comoros | 0.87(0.41,1.44) | 0.41(0.19,0.68) | 0.90(0.45,1.73) | 0.37(0.19,0.72) | -0.39(-0.82,0.04) |
| Congo | 0.50(0.26,0.87) | 0.05(0.02,0.08) | 0.77(0.45,1.31) | 0.04(0.02,0.07) | -0.55(-0.72,-0.39) |
| Cook Islands | 0.00(0.00,0.00) | 0.03(0.01,0.06) | 0.00(0.00,0.01) | 0.06(0.02,0.14) | 1.84(1.11,2.56) |
| Costa Rica | 7.62(5.85,9.64) | 0.68(0.52,0.86) | 3.97(2.83,5.62) | 0.39(0.28,0.55) | -1.78(-2.16,-1.40) |
| Croatia | 4.61(3.38,6.30) | 0.47(0.34,0.64) | 1.01(0.69,1.47) | 0.17(0.12,0.25) | -3.13(-3.52,-2.73) |
| Cuba | 7.80(5.23,11.04) | 0.31(0.21,0.44) | 4.24(2.74,6.31) | 0.24(0.15,0.36) | -0.96(-1.28,-0.63) |
| Cyprus | 0.47(0.23,0.81) | 0.24(0.11,0.41) | 0.55(0.31,0.90) | 0.25(0.14,0.41) | 0.12(-1.23,1.48) |
| Czechia | 15.22(10.52,20.93) | 0.69(0.48,0.95) | 4.61(2.94,6.99) | 0.27(0.17,0.41) | -3.97(-4.81,-3.11) |
| Côte d'Ivoire | 9.35(3.21,17.11) | 0.16(0.06,0.30) | 18.55(6.84,34.07) | 0.16(0.06,0.29) | 0.11(-0.06,0.27) |
| Democratic People's Republic of Korea | 5.80(2.79,10.25) | 0.10(0.05,0.17) | 3.57(1.46,8.54) | 0.07(0.03,0.18) | -0.65(-0.82,-0.48) |
| Democratic Republic of the Congo | 8.67(4.41,15.11) | 0.05(0.02,0.09) | 15.46(9.03,25.17) | 0.04(0.02,0.07) | -0.29(-0.44,-0.14) |
| Denmark | 3.33(2.45,4.50) | 0.38(0.28,0.51) | 1.10(0.77,1.50) | 0.12(0.08,0.16) | -3.35(-4.20,-2.48) |
| Djibouti | 0.69(0.39,1.17) | 0.40(0.23,0.67) | 1.42(0.65,2.60) | 0.34(0.16,0.63) | -0.40(-0.70,-0.09) |
| Dominica | 0.01(0.01,0.02) | 0.05(0.03,0.08) | 0.01(0.01,0.02) | 0.10(0.05,0.16) | 1.95(1.63,2.27) |
| Dominican Republic | 0.36(0.20,0.81) | 0.01(0.01,0.03) | 0.40(0.16,0.86) | 0.01(0.01,0.03) | -0.82(-1.56,-0.09) |
| Ecuador | 4.04(2.92,5.77) | 0.10(0.08,0.15) | 7.49(4.86,11.68) | 0.15(0.10,0.23) | 1.54(0.79,2.30) |
| Egypt | 10.54(4.17,27.68) | 0.05(0.02,0.12) | 14.73(4.58,43.28) | 0.04(0.01,0.12) | -0.19(-1.03,0.66) |
| El Salvador | 3.63(2.28,5.44) | 0.17(0.11,0.25) | 3.53(2.30,5.59) | 0.19(0.13,0.31) | 0.82(0.59,1.05) |
| Equatorial Guinea | 0.12(0.06,0.22) | 0.06(0.03,0.11) | 0.26(0.11,0.62) | 0.05(0.02,0.11) | -1.37(-1.75,-0.99) |
| Eritrea | 7.14(4.16,11.23) | 0.45(0.26,0.71) | 10.00(4.74,17.51) | 0.40(0.19,0.69) | -0.30(-0.45,-0.16) |
| Estonia | 2.21(1.44,3.28) | 0.63(0.41,0.94) | 0.58(0.36,0.86) | 0.27(0.17,0.40) | -1.93(-2.34,-1.52) |
| Eswatini | 0.35(0.21,0.60) | 0.09(0.05,0.16) | 0.41(0.23,0.73) | 0.10(0.06,0.18) | 0.38(0.28,0.47) |
| Ethiopia | 158.74(81.24,273.16) | 0.65(0.33,1.12) | 203.99(114.43,381.44) | 0.46(0.26,0.86) | -1.39(-1.63,-1.14) |
| Fiji | 0.21(0.09,0.46) | 0.07(0.03,0.16) | 0.37(0.16,0.70) | 0.13(0.06,0.26) | 2.37(2.05,2.69) |
| Finland | 2.78(2.08,3.73) | 0.29(0.22,0.39) | 1.47(0.97,2.15) | 0.17(0.11,0.25) | -1.90(-3.02,-0.76) |
| France | 31.04(22.23,42.14) | 0.26(0.19,0.36) | 21.78(13.94,33.24) | 0.19(0.12,0.29) | -1.40(-1.75,-1.05) |
| Gabon | 0.17(0.09,0.28) | 0.04(0.02,0.07) | 0.28(0.15,0.47) | 0.04(0.02,0.07) | 0.26(0.10,0.43) |
| Gambia | 0.71(0.22,1.34) | 0.15(0.05,0.29) | 1.24(0.51,2.37) | 0.12(0.05,0.24) | -0.84(-1.09,-0.59) |
| Georgia | 11.07(5.61,17.02) | 0.81(0.41,1.24) | 2.33(1.49,3.42) | 0.32(0.20,0.46) | -2.75(-3.40,-2.09) |
| Germany | 50.51(36.90,67.38) | 0.39(0.29,0.52) | 30.86(21.46,44.77) | 0.26(0.18,0.37) | -1.21(-2.18,-0.24) |
| Ghana | 0.42(0.13,0.94) | 0.01(0.00,0.01) | 0.27(0.09,0.65) | 0.00(0.00,0.01) | -5.14(-6.03,-4.23) |
| Greece | 19.46(14.97,25.25) | 0.96(0.74,1.25) | 6.34(4.75,8.48) | 0.45(0.34,0.61) | -2.24(-2.59,-1.90) |
| Greenland | 0.01(0.00,0.03) | 0.10(0.02,0.21) | 0.01(0.00,0.01) | 0.04(0.03,0.07) | -2.48(-3.09,-1.86) |
| Grenada | 0.02(0.01,0.03) | 0.05(0.03,0.08) | 0.01(0.01,0.02) | 0.05(0.03,0.07) | -0.44(-1.15,0.29) |
| Guam | 0.04(0.02,0.07) | 0.09(0.05,0.16) | 0.03(0.01,0.07) | 0.09(0.04,0.18) | 0.14(-0.89,1.19) |
| Guatemala | 5.31(2.98,6.79) | 0.13(0.07,0.17) | 3.31(2.63,4.25) | 0.07(0.05,0.09) | -1.20(-1.47,-0.93) |
| Guinea | 7.35(2.03,14.44) | 0.27(0.07,0.52) | 11.32(4.01,24.56) | 0.19(0.07,0.41) | -0.95(-1.06,-0.84) |
| Guinea-Bissau | 0.27(0.12,0.45) | 0.06(0.03,0.09) | 0.18(0.07,0.41) | 0.02(0.01,0.05) | -3.89(-4.23,-3.56) |
| Guyana | 0.17(0.15,0.21) | 0.06(0.05,0.07) | 0.11(0.08,0.14) | 0.05(0.04,0.07) | 0.49(-0.17,1.16) |
| Haiti | 2.86(1.29,5.05) | 0.11(0.05,0.19) | 3.90(1.91,6.45) | 0.09(0.04,0.15) | -0.35(-0.43,-0.27) |
| Honduras | 2.18(1.06,3.30) | 0.10(0.05,0.15) | 1.45(0.70,2.66) | 0.04(0.02,0.08) | -2.71(-2.91,-2.51) |
| Hungary | 6.33(4.21,9.12) | 0.30(0.20,0.43) | 1.50(0.98,2.23) | 0.11(0.07,0.16) | -2.70(-3.57,-1.81) |
| Iceland | 0.39(0.27,0.52) | 0.61(0.43,0.83) | 0.21(0.14,0.32) | 0.31(0.20,0.47) | -3.36(-4.91,-1.79) |
| India | 807.56(387.16,1118.73) | 0.25(0.12,0.34) | 591.29(350.64,909.00) | 0.16(0.10,0.25) | -1.25(-1.43,-1.07) |
| Indonesia | 36.66(24.19,69.46) | 0.05(0.04,0.10) | 26.24(12.96,59.91) | 0.04(0.02,0.09) | -1.13(-1.29,-0.97) |
| Iran (Islamic Republic of) | 35.40(22.85,54.76) | 0.14(0.09,0.22) | 72.47(30.51,119.57) | 0.36(0.15,0.59) | 3.78(3.22,4.33) |
| Iraq | 24.82(9.83,41.85) | 0.30(0.12,0.51) | 40.28(18.03,74.54) | 0.30(0.13,0.55) | 0.36(0.15,0.56) |
| Ireland | 3.44(2.48,4.83) | 0.35(0.25,0.49) | 1.89(1.25,2.67) | 0.19(0.13,0.27) | -2.31(-3.32,-1.30) |
| Israel | 3.54(2.63,4.66) | 0.23(0.17,0.30) | 4.31(3.08,5.88) | 0.16(0.12,0.22) | -0.58(-1.69,0.55) |
| Italy | 48.10(37.74,60.52) | 0.52(0.41,0.66) | 30.22(23.97,36.72) | 0.40(0.32,0.48) | -0.84(-1.18,-0.51) |
| Jamaica | 1.70(1.22,2.29) | 0.20(0.15,0.27) | 0.27(0.18,0.40) | 0.05(0.03,0.07) | -4.87(-5.85,-3.89) |
| Japan | 4.78(3.92,5.77) | 0.02(0.02,0.02) | 4.57(3.60,5.76) | 0.03(0.02,0.04) | 1.56(1.34,1.79) |
| Jordan | 1.62(0.99,2.69) | 0.10(0.06,0.16) | 6.80(3.91,10.54) | 0.19(0.11,0.29) | 2.83(2.33,3.33) |
| Kazakhstan | 9.96(8.08,12.72) | 0.19(0.16,0.24) | 8.87(6.49,11.63) | 0.16(0.12,0.21) | -0.20(-0.50,0.10) |
| Kenya | 15.57(9.79,25.60) | 0.14(0.09,0.23) | 25.78(13.68,53.95) | 0.14(0.07,0.29) | 0.78(0.40,1.17) |
| Kiribati | 0.00(0.00,0.01) | 0.01(0.00,0.03) | 0.01(0.00,0.01) | 0.01(0.00,0.03) | 0.12(0.01,0.22) |
| Kuwait | 5.09(3.74,7.03) | 0.92(0.67,1.27) | 2.07(1.41,2.82) | 0.24(0.17,0.33) | 0.81(-1.60,3.28) |
| Kyrgyzstan | 6.59(4.39,9.28) | 0.39(0.26,0.55) | 3.08(1.89,4.59) | 0.14(0.08,0.20) | -1.42(-2.57,-0.26) |
| Lao People's Democratic Republic | 1.19(0.62,2.43) | 0.06(0.03,0.13) | 0.88(0.38,2.10) | 0.04(0.02,0.09) | -1.67(-1.76,-1.58) |
| Latvia | 3.23(2.09,4.65) | 0.57(0.37,0.82) | 0.42(0.25,0.65) | 0.14(0.09,0.22) | -3.37(-3.77,-2.97) |
| Lebanon | 4.60(2.24,8.01) | 0.44(0.21,0.77) | 7.95(3.92,14.31) | 0.62(0.31,1.12) | 1.10(0.70,1.50) |
| Lesotho | 0.50(0.27,0.88) | 0.07(0.04,0.13) | 0.64(0.33,1.20) | 0.10(0.05,0.19) | 1.50(1.31,1.68) |
| Liberia | 0.50(0.22,0.84) | 0.04(0.02,0.07) | 0.55(0.24,1.14) | 0.03(0.01,0.05) | -2.81(-3.29,-2.33) |
| Libya | 9.25(4.84,14.73) | 0.51(0.27,0.81) | 12.35(6.32,20.06) | 0.83(0.42,1.34) | 2.28(1.95,2.62) |
| Lithuania | 4.94(3.53,6.74) | 0.59(0.42,0.81) | 0.94(0.59,1.40) | 0.23(0.14,0.34) | -3.18(-3.59,-2.78) |
| Luxembourg | 0.12(0.09,0.15) | 0.18(0.14,0.23) | 0.10(0.07,0.14) | 0.10(0.07,0.13) | -3.28(-3.83,-2.71) |
| Madagascar | 19.78(11.09,31.40) | 0.36(0.20,0.58) | 35.07(17.94,60.34) | 0.30(0.15,0.51) | -0.38(-0.51,-0.26) |
| Malawi | 15.13(7.26,26.07) | 0.33(0.16,0.57) | 17.15(6.65,32.75) | 0.21(0.08,0.40) | -1.39(-1.55,-1.23) |
| Malaysia | 6.15(2.13,12.07) | 0.09(0.03,0.18) | 7.39(2.95,12.37) | 0.10(0.04,0.16) | 0.18(-0.20,0.56) |
| Maldives | 0.08(0.04,0.13) | 0.08(0.04,0.13) | 0.11(0.06,0.18) | 0.11(0.06,0.18) | 1.30(1.11,1.50) |
| Mali | 13.64(3.40,27.03) | 0.33(0.08,0.65) | 24.28(8.67,49.97) | 0.21(0.07,0.43) | -1.29(-1.42,-1.16) |
| Malta | 0.27(0.19,0.35) | 0.30(0.22,0.40) | 0.43(0.28,0.67) | 0.67(0.44,1.04) | 0.11(-1.39,1.63) |
| Marshall Islands | 0.00(0.00,0.01) | 0.01(0.00,0.03) | 0.00(0.00,0.01) | 0.02(0.00,0.03) | 0.25(0.01,0.49) |
| Mauritania | 0.34(0.17,0.57) | 0.04(0.02,0.06) | 0.38(0.16,0.83) | 0.02(0.01,0.04) | -2.71(-3.07,-2.35) |
| Mauritius | 0.30(0.24,0.36) | 0.09(0.07,0.11) | 0.19(0.15,0.24) | 0.09(0.07,0.12) | -1.92(-3.12,-0.71) |
| Mexico | 128.47(118.88,140.16) | 0.38(0.36,0.42) | 57.45(50.13,66.17) | 0.18(0.16,0.21) | -2.04(-2.27,-1.80) |
| Micronesia (Federated States of) | 0.01(0.00,0.02) | 0.02(0.01,0.04) | 0.00(0.00,0.01) | 0.01(0.00,0.03) | -0.80(-0.90,-0.71) |
| Monaco | 0.03(0.01,0.06) | 0.85(0.39,1.68) | 0.04(0.02,0.11) | 0.83(0.34,2.14) | -0.80(-1.13,-0.46) |
| Mongolia | 1.16(0.51,2.38) | 0.13(0.06,0.26) | 1.74(0.94,2.88) | 0.16(0.09,0.27) | 0.97(0.34,1.61) |
| Montenegro | 1.31(0.82,1.89) | 0.81(0.51,1.17) | 0.60(0.36,1.08) | 0.54(0.32,0.97) | -1.07(-1.70,-0.44) |
| Morocco | 27.72(13.59,46.39) | 0.28(0.14,0.47) | 21.94(11.18,39.33) | 0.22(0.11,0.40) | -0.35(-0.54,-0.16) |
| Mozambique | 36.03(19.81,55.45) | 0.58(0.32,0.89) | 56.41(21.55,105.27) | 0.40(0.15,0.74) | -0.80(-1.06,-0.55) |
| Myanmar | 10.83(5.24,21.89) | 0.07(0.04,0.15) | 7.17(2.88,17.13) | 0.05(0.02,0.11) | -1.89(-2.12,-1.65) |
| Namibia | 0.72(0.30,1.21) | 0.12(0.05,0.20) | 1.39(0.66,2.40) | 0.17(0.08,0.29) | 1.35(1.16,1.54) |
| Nauru | 0.00(0.00,0.00) | 0.02(0.01,0.04) | 0.00(0.00,0.00) | 0.02(0.01,0.04) | 0.28(0.15,0.42) |
| Nepal | 27.65(13.50,52.56) | 0.33(0.16,0.62) | 21.63(9.34,50.96) | 0.23(0.10,0.55) | -0.82(-1.03,-0.61) |
| Netherlands | 18.00(13.44,24.08) | 0.66(0.49,0.88) | 12.76(8.86,17.78) | 0.48(0.33,0.66) | -0.41(-0.74,-0.08) |
| New Zealand | 0.57(0.40,0.81) | 0.07(0.05,0.10) | 0.69(0.45,1.04) | 0.07(0.05,0.11) | -0.63(-2.10,0.85) |
| Nicaragua | 2.69(1.63,4.04) | 0.15(0.09,0.22) | 1.78(1.08,3.13) | 0.09(0.05,0.16) | -0.77(-1.06,-0.47) |
| Niger | 2.28(1.04,3.88) | 0.06(0.03,0.10) | 2.55(1.05,5.83) | 0.02(0.01,0.05) | -3.93(-4.33,-3.53) |
| Nigeria | 311.56(54.69,551.24) | 0.80(0.14,1.41) | 679.40(126.87,1284.03) | 0.67(0.12,1.26) | -0.69(-0.88,-0.51) |
| Niue | 0.00(0.00,0.00) | 0.02(0.01,0.04) | 0.00(0.00,0.00) | 0.07(0.03,0.15) | 1.54(0.47,2.63) |
| North Macedonia | 2.44(1.44,3.49) | 0.46(0.27,0.66) | 1.46(0.89,2.70) | 0.45(0.27,0.83) | 0.18(-0.22,0.58) |
| Northern Mariana Islands | 0.00(0.00,0.00) | 0.01(0.00,0.02) | 0.00(0.00,0.00) | 0.01(0.01,0.02) | 2.86(2.48,3.25) |
| Norway | 0.50(0.39,0.62) | 0.06(0.05,0.08) | 0.11(0.08,0.13) | 0.01(0.01,0.01) | -4.89(-6.69,-3.06) |
| Oman | 2.05(0.86,3.57) | 0.24(0.10,0.42) | 3.35(1.41,5.96) | 0.27(0.11,0.49) | 0.88(0.54,1.21) |
| Pakistan | 292.68(144.84,439.17) | 0.59(0.29,0.89) | 632.19(342.41,1013.54) | 0.74(0.40,1.19) | 0.83(0.71,0.96) |
| Palau | 0.00(0.00,0.00) | 0.00(0.00,0.00) | 0.00(0.00,0.00) | 0.00(0.00,0.00) | -0.19(-0.32,-0.06) |
| Palestine | 2.78(1.63,4.19) | 0.29(0.17,0.43) | 6.78(3.80,11.07) | 0.36(0.20,0.59) | 1.38(0.89,1.87) |
| Panama | 1.40(1.06,1.74) | 0.17(0.13,0.21) | 2.06(1.55,2.75) | 0.18(0.13,0.24) | 0.25(0.00,0.49) |
| Papua New Guinea | 0.29(0.08,0.62) | 0.02(0.00,0.04) | 0.59(0.19,1.25) | 0.02(0.00,0.03) | -0.38(-0.49,-0.27) |
| Paraguay | 1.98(1.09,3.23) | 0.12(0.07,0.19) | 2.65(1.50,4.38) | 0.13(0.07,0.22) | 0.45(0.13,0.77) |
| Peru | 9.19(5.78,14.56) | 0.11(0.07,0.18) | 13.55(7.20,24.65) | 0.14(0.08,0.26) | 1.29(1.08,1.49) |
| Philippines | 8.03(4.91,12.30) | 0.03(0.02,0.05) | 9.40(6.26,13.72) | 0.03(0.02,0.04) | -0.19(-0.41,0.02) |
| Poland | 34.26(28.48,41.77) | 0.36(0.30,0.44) | 9.03(7.30,11.03) | 0.15(0.12,0.19) | -3.20(-3.86,-2.53) |
| Portugal | 6.98(4.96,9.92) | 0.33(0.23,0.47) | 3.13(2.03,4.53) | 0.23(0.15,0.33) | -1.82(-2.16,-1.48) |
| Puerto Rico | 1.61(1.18,2.22) | 0.16(0.12,0.22) | 0.37(0.24,0.53) | 0.08(0.06,0.12) | -1.71(-2.29,-1.13) |
| Qatar | 0.10(0.05,0.20) | 0.08(0.04,0.16) | 0.81(0.38,1.69) | 0.16(0.08,0.34) | 3.26(2.71,3.82) |
| Republic of Korea | 1.95(1.22,3.30) | 0.02(0.01,0.03) | 1.67(0.94,3.05) | 0.03(0.02,0.05) | 2.24(1.55,2.94) |
| Republic of Moldova | 10.14(8.03,12.88) | 0.82(0.65,1.04) | 2.06(1.48,2.73) | 0.39(0.28,0.52) | -2.24(-2.72,-1.76) |
| Romania | 23.11(16.72,31.28) | 0.41(0.30,0.56) | 5.79(3.58,8.86) | 0.19(0.12,0.29) | -3.04(-3.53,-2.56) |
| Russian Federation | 226.67(213.09,241.63) | 0.65(0.61,0.70) | 80.63(73.52,87.28) | 0.31(0.28,0.33) | -2.23(-2.66,-1.81) |
| Rwanda | 19.18(11.24,29.72) | 0.57(0.33,0.88) | 16.75(7.18,32.37) | 0.34(0.14,0.65) | -1.96(-2.14,-1.77) |
| Saint Kitts and Nevis | 0.00(0.00,0.00) | 0.00(0.00,0.00) | 0.00(0.00,0.00) | 0.02(0.01,0.02) | 7.13(4.50,9.84) |
| Saint Lucia | 0.05(0.04,0.06) | 0.09(0.08,0.13) | 0.03(0.02,0.05) | 0.11(0.08,0.16) | -0.05(-0.61,0.52) |
| Saint Vincent and the Grenadines | 0.05(0.04,0.06) | 0.11(0.09,0.14) | 0.02(0.02,0.03) | 0.08(0.06,0.11) | -0.91(-1.54,-0.28) |
| Samoa | 0.14(0.07,0.27) | 0.20(0.09,0.37) | 0.14(0.06,0.30) | 0.18(0.07,0.37) | -0.48(-0.59,-0.37) |
| San Marino | 0.04(0.02,0.08) | 1.05(0.55,1.89) | 0.03(0.01,0.05) | 0.60(0.30,1.24) | -1.59(-1.80,-1.38) |
| Sao Tome and Principe | 0.00(0.00,0.00) | 0.00(0.00,0.00) | 0.00(0.00,0.00) | 0.00(0.00,0.00) | -1.46(-1.70,-1.22) |
| Saudi Arabia | 8.31(1.87,17.39) | 0.13(0.03,0.27) | 10.68(1.92,23.10) | 0.14(0.03,0.31) | 0.67(0.12,1.22) |
| Senegal | 1.80(0.84,3.07) | 0.05(0.02,0.08) | 1.30(0.55,2.97) | 0.02(0.01,0.05) | -3.47(-3.77,-3.16) |
| Serbia | 8.48(4.38,14.34) | 0.39(0.20,0.66) | 3.44(1.60,7.53) | 0.26(0.12,0.57) | -1.68(-1.98,-1.38) |
| Seychelles | 0.02(0.01,0.03) | 0.07(0.04,0.11) | 0.01(0.00,0.02) | 0.04(0.02,0.07) | -0.21(-0.94,0.53) |
| Sierra Leone | 0.79(0.33,1.38) | 0.04(0.02,0.08) | 0.81(0.39,1.67) | 0.02(0.01,0.05) | -2.58(-2.85,-2.31) |
| Singapore | 0.26(0.20,0.33) | 0.04(0.03,0.05) | 0.33(0.23,0.47) | 0.04(0.03,0.06) | 2.60(1.15,4.07) |
| Slovakia | 4.74(2.69,7.27) | 0.36(0.20,0.55) | 2.51(1.40,4.42) | 0.29(0.16,0.52) | -0.56(-0.83,-0.29) |
| Slovenia | 2.12(1.54,2.84) | 0.51(0.37,0.69) | 0.54(0.37,0.75) | 0.17(0.12,0.24) | -2.55(-3.39,-1.71) |
| Solomon Islands | 0.02(0.01,0.05) | 0.02(0.01,0.03) | 0.04(0.01,0.08) | 0.01(0.00,0.03) | -0.05(-0.23,0.12) |
| Somalia | 14.76(7.87,24.20) | 0.38(0.20,0.62) | 32.02(13.79,59.90) | 0.31(0.13,0.58) | -0.39(-0.74,-0.05) |
| South Africa | 8.84(6.31,14.63) | 0.06(0.05,0.11) | 14.11(8.91,20.64) | 0.09(0.06,0.14) | 0.60(0.25,0.96) |
| South Sudan | 9.69(5.25,15.94) | 0.37(0.20,0.61) | 18.86(10.95,29.63) | 0.44(0.25,0.69) | 0.73(0.21,1.25) |
| Spain | 35.69(25.73,48.46) | 0.46(0.33,0.62) | 19.35(13.03,27.66) | 0.30(0.20,0.43) | -1.75(-2.06,-1.45) |
| Sri Lanka | 6.71(3.89,10.14) | 0.12(0.07,0.18) | 5.52(2.84,10.65) | 0.11(0.06,0.21) | -1.02(-1.60,-0.44) |
| Sudan | 16.54(8.31,32.14) | 0.19(0.09,0.36) | 35.45(19.56,62.32) | 0.21(0.12,0.38) | 0.72(0.61,0.83) |
| Suriname | 0.08(0.04,0.12) | 0.06(0.03,0.10) | 0.10(0.06,0.17) | 0.07(0.04,0.12) | 0.60(0.27,0.94) |
| Sweden | 1.70(1.24,2.33) | 0.11(0.08,0.15) | 1.98(1.36,2.82) | 0.11(0.07,0.15) | 0.57(-0.76,1.92) |
| Switzerland | 5.46(3.91,7.14) | 0.47(0.34,0.62) | 1.69(1.20,2.28) | 0.13(0.09,0.17) | -4.49(-5.51,-3.45) |
| Syrian Arab Republic | 0.58(0.38,0.87) | 0.01(0.01,0.01) | 0.70(0.38,1.03) | 0.02(0.01,0.03) | 2.68(2.16,3.19) |
| Taiwan (Province of China) | 5.40(4.07,7.28) | 0.10(0.07,0.13) | 1.61(1.14,2.16) | 0.05(0.04,0.07) | -2.16(-2.72,-1.60) |
| Tajikistan | 1.28(0.61,2.23) | 0.06(0.03,0.10) | 1.92(1.01,3.58) | 0.05(0.03,0.10) | -0.81(-1.20,-0.43) |
| Thailand | 15.20(8.40,23.82) | 0.09(0.05,0.14) | 7.22(3.58,15.09) | 0.07(0.04,0.15) | -1.15(-1.46,-0.83) |
| Timor-Leste | 0.18(0.09,0.33) | 0.05(0.03,0.10) | 0.19(0.08,0.45) | 0.04(0.02,0.09) | -1.08(-1.46,-0.71) |
| Togo | 0.75(0.37,1.28) | 0.04(0.02,0.07) | 0.61(0.28,1.25) | 0.02(0.01,0.04) | -3.39(-3.72,-3.05) |
| Tokelau | 0.00(0.00,0.00) | 0.02(0.01,0.04) | 0.00(0.00,0.00) | 0.09(0.02,0.23) | 1.65(0.05,3.28) |
| Tonga | 0.00(0.00,0.01) | 0.01(0.00,0.02) | 0.00(0.00,0.01) | 0.01(0.00,0.02) | 0.14(0.01,0.27) |
| Trinidad and Tobago | 0.29(0.20,0.37) | 0.07(0.05,0.09) | 0.14(0.11,0.19) | 0.05(0.04,0.07) | -1.06(-1.43,-0.70) |
| Tunisia | 20.79(10.68,32.52) | 0.67(0.34,1.05) | 19.18(9.83,33.77) | 0.69(0.36,1.22) | 0.08(-0.04,0.20) |
| Turkey | 3.59(2.77,4.58) | 0.24(0.18,0.31) | 6.27(4.65,8.41) | 0.41(0.31,0.55) | 1.71(1.27,2.15) |
| Turkmenistan | 0.00(0.00,0.00) | 0.02(0.01,0.04) | 0.00(0.00,0.00) | 0.02(0.01,0.03) | -0.76(-0.84,-0.68) |
| Tuvalu | 40.97(24.42,74.08) | 0.20(0.12,0.36) | 44.94(25.09,85.46) | 0.24(0.14,0.46) | 1.10(0.61,1.58) |
| Uganda | 44.69(24.47,67.97) | 0.53(0.29,0.81) | 119.01(51.22,207.86) | 0.60(0.26,1.05) | 0.33(0.18,0.47) |
| Ukraine | 28.93(17.51,40.58) | 0.25(0.15,0.36) | 13.14(9.26,18.47) | 0.21(0.15,0.29) | -1.28(-1.55,-1.01) |
| United Arab Emirates | 0.66(0.33,1.22) | 0.11(0.06,0.21) | 1.21(0.68,2.51) | 0.09(0.05,0.19) | -0.51(-0.88,-0.14) |
| United Kingdom | 27.24(25.15,29.37) | 0.25(0.23,0.27) | 21.68(19.72,23.53) | 0.18(0.17,0.20) | -0.53(-0.96,-0.11) |
| United Republic of Tanzania | 56.46(32.41,85.15) | 0.47(0.27,0.71) | 91.05(45.98,158.46) | 0.37(0.19,0.65) | -0.29(-0.54,-0.04) |
| United States Virgin Islands | 0.01(0.00,0.01) | 0.02(0.01,0.05) | 0.00(0.00,0.00) | 0.02(0.01,0.04) | -0.63(-1.10,-0.16) |
| United States of America | 149.21(142.21,157.08) | 0.27(0.25,0.28) | 72.75(67.22,78.32) | 0.12(0.11,0.13) | -2.84(-3.15,-2.52) |
| Uruguay | 1.54(1.13,2.05) | 0.19(0.14,0.25) | 1.36(0.95,2.03) | 0.21(0.14,0.31) | 0.03(-0.64,0.70) |
| Uzbekistan | 24.58(16.25,35.18) | 0.29(0.19,0.41) | 25.20(16.58,36.37) | 0.25(0.16,0.36) | -0.19(-0.46,0.09) |
| Vanuatu | 0.01(0.00,0.02) | 0.01(0.00,0.03) | 0.02(0.00,0.03) | 0.01(0.00,0.03) | 0.14(-0.04,0.33) |
| Venezuela (Bolivarian Republic of) | 12.05(9.88,14.46) | 0.17(0.14,0.20) | 15.90(11.07,21.77) | 0.24(0.17,0.33) | 1.23(0.91,1.55) |
| Viet Nam | 28.08(10.99,51.42) | 0.11(0.04,0.19) | 42.39(16.96,78.65) | 0.17(0.07,0.32) | 1.58(1.50,1.65) |
| Yemen | 9.60(5.37,16.88) | 0.14(0.08,0.24) | 21.85(12.13,38.90) | 0.16(0.09,0.28) | 0.55(0.45,0.65) |
| Zambia | 15.41(8.69,25.02) | 0.41(0.23,0.67) | 26.05(12.44,48.68) | 0.31(0.15,0.59) | -0.79(-0.98,-0.61) |
| Zimbabwe | 4.35(1.79,7.27) | 0.09(0.04,0.15) | 9.37(4.55,15.10) | 0.15(0.07,0.24) | 2.66(1.85,3.48) |
| ^a^EAPC is expressed as 95% CIs | | | | | |

**Table S2** Incidence of Non-Hodgkin Lymphoma in Children Between 1990 and 2021 at 204 Countries and Territories.

| Location | Rate per 100000 (95% UI) | | | | |
| --- | --- | --- | --- | --- | --- |
|  | 1990 | | 2021 | | EAPC^a^ |
|  | Cases | Rates | Cases | Rates |  |
| Global | 21469.72(17405.39,24965.96) | 1.23(1.00,1.44) | 20788.76(17199.49,25305.83) | 1.03(0.85,1.26) | -0.43(-0.55,-0.32) |
| 5 SDI |  |  |  |  |  |
| High SDI | 2645.39(2521.37,2767.43) | 1.42(1.36,1.49) | 2249.56(2041.06,2443.45) | 1.30(1.18,1.42) | -0.31(-0.55,-0.08) |
| High-middle SDI | 3779.54(3389.04,4173.63) | 1.38(1.24,1.53) | 2510.88(2191.23,2927.62) | 1.09(0.95,1.27) | -0.53(-0.72,-0.34) |
| Middle SDI | 5677.01(4774.10,6530.52) | 0.98(0.83,1.13) | 4799.77(3948.21,5966.06) | 0.85(0.70,1.05) | -0.26(-0.44,-0.08) |
| Low-middle SDI | 4970.40(3345.75,6266.44) | 1.05(0.71,1.33) | 5277.19(4217.08,6668.60) | 0.91(0.73,1.15) | -0.34(-0.43,-0.24) |
| Low SDI | 4377.35(2848.30,5768.16) | 1.91(1.24,2.52) | 5932.40(3988.74,8047.35) | 1.29(0.87,1.75) | -1.16(-1.28,-1.04) |
| 21 GBD Region | | | | | |
| Andean Latin America | 169.78(141.72,210.93) | 1.14(0.95,1.42) | 218.50(160.85,287.45) | 1.21(0.89,1.59) | 0.46(0.25,0.66) |
| Australasia | 75.26(63.65,89.03) | 1.64(1.39,1.94) | 67.16(52.43,84.09) | 1.17(0.91,1.47) | -1.35(-2.14,-0.54) |
| Caribbean | 231.64(161.79,289.06) | 2.03(1.42,2.53) | 183.94(126.81,247.11) | 1.60(1.10,2.15) | -0.56(-0.66,-0.45) |
| Central Asia | 324.46(265.47,382.95) | 1.30(1.06,1.53) | 298.46(242.89,371.99) | 1.08(0.88,1.34) | -0.60(-0.83,-0.36) |
| Central Europe | 321.12(295.65,347.57) | 1.09(1.00,1.18) | 193.03(169.75,219.87) | 1.09(0.96,1.24) | 0.29(-0.10,0.69) |
| Central Latin America | 566.05(522.70,615.90) | 0.88(0.81,0.96) | 485.28(406.15,575.58) | 0.76(0.64,0.91) | 0.05(-0.27,0.36) |
| Central Sub-Saharan Africa | 227.93(67.60,349.57) | 0.90(0.27,1.38) | 243.55(158.69,340.70) | 0.42(0.27,0.58) | -2.15(-2.34,-1.96) |
| East Asia | 3701.78(3060.56,4553.18) | 1.12(0.93,1.38) | 2028.44(1606.73,2618.11) | 0.76(0.60,0.98) | -1.15(-1.56,-0.74) |
| Eastern Europe | 1100.28(1037.65,1171.54) | 2.14(2.02,2.28) | 443.59(405.55,486.16) | 1.25(1.14,1.37) | -1.66(-2.37,-0.95) |
| Eastern Sub-Saharan Africa | 2328.46(1629.73,3042.01) | 2.57(1.80,3.36) | 2916.18(1898.38,4221.69) | 1.63(1.06,2.37) | -1.28(-1.43,-1.13) |
| High-income Asia Pacific | 328.52(285.55,383.14) | 0.93(0.81,1.09) | 236.81(193.98,286.82) | 1.06(0.86,1.28) | 0.36(0.11,0.62) |
| High-income North America | 972.61(919.58,1029.72) | 1.58(1.49,1.67) | 683.85(605.47,757.17) | 1.04(0.92,1.15) | -1.43(-1.58,-1.27) |
| North Africa and Middle East | 1528.65(1142.55,1980.11) | 1.09(0.81,1.41) | 1848.35(1540.59,2309.64) | 1.01(0.84,1.26) | 0.10(-0.12,0.33) |
| Oceania | 23.78(15.62,34.43) | 0.89(0.58,1.28) | 63.97(39.80,94.99) | 1.26(0.78,1.87) | 1.14(0.91,1.37) |
| South Asia | 4446.04(2790.87,5707.71) | 1.03(0.64,1.32) | 4514.77(3508.85,5872.59) | 0.89(0.69,1.16) | -0.34(-0.49,-0.18) |
| Southeast Asia | 1181.40(780.70,1556.84) | 0.69(0.46,0.91) | 1071.56(867.43,1427.84) | 0.62(0.50,0.83) | -0.35(-0.43,-0.27) |
| Southern Latin America | 127.32(108.81,149.47) | 0.85(0.73,1.00) | 114.08(92.04,141.44) | 0.79(0.63,0.98) | 0.03(-0.36,0.43) |
| Southern Sub-Saharan Africa | 124.50(97.76,159.74) | 0.60(0.47,0.77) | 249.55(186.77,317.54) | 1.04(0.78,1.32) | 2.25(1.72,2.78) |
| Tropical Latin America | 482.92(428.99,538.39) | 0.90(0.80,1.00) | 292.47(234.72,348.22) | 0.58(0.47,0.69) | -0.93(-1.43,-0.43) |
| Western Europe | 1508.30(1381.87,1663.71) | 2.12(1.95,2.34) | 1421.96(1252.50,1601.73) | 2.09(1.84,2.35) | -0.21(-0.57,0.15) |
| Western Sub-Saharan Africa | 1698.91(1188.70,2245.72) | 1.93(1.35,2.56) | 3213.26(1906.57,4577.45) | 1.50(0.89,2.13) | -0.66(-0.80,-0.51) |
| 204 countries and territories | | | | | |
| Afghanistan | 36.96(13.05,63.56) | 0.86(0.30,1.48) | 82.39(50.72,131.86) | 0.58(0.36,0.93) | -0.90(-1.15,-0.64) |
| Albania | 9.27(6.54,12.45) | 0.83(0.59,1.11) | 4.58(2.91,7.01) | 1.03(0.66,1.58) | 1.44(0.34,2.56) |
| Algeria | 157.21(109.22,215.26) | 1.47(1.02,2.01) | 192.98(131.84,276.98) | 1.45(0.99,2.08) | 0.14(-0.01,0.29) |
| American Samoa | 0.15(0.10,0.24) | 0.79(0.51,1.26) | 0.27(0.15,0.45) | 1.90(1.08,3.17) | 3.24(2.97,3.51) |
| Andorra | 0.66(0.38,1.02) | 6.94(4.00,10.73) | 0.33(0.21,0.51) | 3.24(2.09,5.02) | -2.22(-2.60,-1.84) |
| Angola | 61.13(12.78,106.29) | 1.30(0.27,2.25) | 74.34(44.69,113.28) | 0.49(0.29,0.74) | -2.95(-3.23,-2.67) |
| Antigua and Barbuda | 0.16(0.12,0.19) | 0.85(0.66,1.05) | 0.21(0.16,0.27) | 1.25(0.97,1.60) | 1.35(0.76,1.94) |
| Argentina | 93.66(75.38,115.10) | 0.92(0.74,1.14) | 85.57(66.01,111.74) | 0.84(0.65,1.10) | 0.02(-0.36,0.40) |
| Armenia | 7.92(5.42,10.24) | 0.76(0.52,0.98) | 6.82(4.79,9.58) | 1.15(0.81,1.62) | 3.12(2.46,3.78) |
| Australia | 59.24(48.67,73.04) | 1.56(1.29,1.93) | 45.41(33.27,60.76) | 0.96(0.70,1.28) | -1.64(-2.32,-0.94) |
| Austria | 26.35(20.72,33.84) | 1.95(1.54,2.51) | 22.76(17.31,29.63) | 1.75(1.33,2.28) | -0.31(-0.69,0.08) |
| Azerbaijan | 22.42(15.19,31.84) | 0.92(0.63,1.31) | 28.07(16.60,42.85) | 1.19(0.70,1.82) | 0.43(0.19,0.67) |
| Bahamas | 0.84(0.69,1.04) | 1.05(0.86,1.29) | 0.81(0.60,1.11) | 1.00(0.73,1.37) | -0.38(-1.01,0.26) |
| Bahrain | 1.17(0.86,1.61) | 0.72(0.53,0.98) | 2.95(1.96,4.29) | 0.99(0.66,1.45) | 1.23(0.84,1.62) |
| Bangladesh | 771.00(330.27,1140.61) | 1.58(0.68,2.33) | 561.48(355.08,958.16) | 1.23(0.78,2.09) | -0.55(-0.76,-0.34) |
| Barbados | 1.26(1.04,1.56) | 2.03(1.66,2.51) | 0.82(0.57,1.15) | 1.73(1.20,2.44) | 0.45(0.00,0.90) |
| Belarus | 22.80(16.85,31.26) | 0.95(0.70,1.30) | 45.99(31.36,66.62) | 2.91(1.99,4.22) | 5.19(4.46,5.93) |
| Belgium | 35.45(26.17,46.93) | 1.96(1.45,2.60) | 49.60(34.79,70.28) | 2.59(1.82,3.68) | 0.10(-0.27,0.47) |
| Belize | 0.80(0.66,0.95) | 0.97(0.81,1.16) | 0.61(0.48,0.77) | 0.50(0.39,0.63) | -1.51(-1.82,-1.19) |
| Benin | 38.55(25.12,56.51) | 1.59(1.04,2.33) | 78.26(48.54,114.59) | 1.29(0.80,1.88) | -0.49(-0.63,-0.35) |
| Bermuda | 0.21(0.16,0.28) | 1.79(1.36,2.37) | 0.25(0.18,0.35) | 2.91(2.09,4.11) | 2.01(1.49,2.53) |
| Bhutan | 3.20(1.36,5.20) | 1.22(0.52,1.98) | 2.04(1.15,3.78) | 1.09(0.61,2.02) | -0.76(-1.05,-0.47) |
| Bolivia (Plurinational State of) | 42.39(29.87,57.77) | 1.58(1.11,2.15) | 34.94(24.95,49.66) | 1.00(0.72,1.42) | -1.51(-1.61,-1.42) |
| Bosnia and Herzegovina | 7.18(5.21,9.55) | 0.66(0.48,0.87) | 4.82(2.96,7.00) | 0.98(0.60,1.43) | 2.10(1.56,2.65) |
| Botswana | 2.50(1.68,3.79) | 0.42(0.28,0.64) | 5.56(3.44,8.46) | 0.80(0.49,1.21) | 2.83(2.37,3.30) |
| Brazil | 468.22(413.47,522.67) | 0.90(0.80,1.01) | 272.57(217.18,322.50) | 0.57(0.45,0.67) | -1.01(-1.54,-0.48) |
| Brunei Darussalam | 0.78(0.56,1.05) | 0.86(0.62,1.16) | 0.65(0.47,0.85) | 0.68(0.50,0.89) | 0.39(-0.14,0.93) |
| Bulgaria | 21.89(18.11,26.89) | 1.26(1.04,1.55) | 13.38(9.90,18.00) | 1.37(1.01,1.84) | 0.41(-0.06,0.88) |
| Burkina Faso | 73.82(45.28,107.87) | 1.56(0.96,2.29) | 134.62(90.96,185.50) | 1.30(0.88,1.79) | -0.25(-0.45,-0.05) |
| Burundi | 77.43(48.24,119.73) | 2.95(1.84,4.57) | 67.80(35.07,124.76) | 1.16(0.60,2.13) | -2.45(-2.70,-2.20) |
| Cabo Verde | 0.43(0.24,0.74) | 0.27(0.15,0.47) | 2.74(1.09,4.03) | 1.91(0.76,2.81) | 5.43(4.55,6.32) |
| Cambodia | 33.63(14.65,49.44) | 0.72(0.31,1.06) | 28.10(19.01,45.92) | 0.55(0.37,0.90) | -1.23(-1.49,-0.97) |
| Cameroon | 74.90(49.07,103.81) | 1.53(1.01,2.13) | 195.74(128.80,283.49) | 1.45(0.96,2.11) | 0.15(-0.03,0.33) |
| Canada | 87.55(67.82,113.80) | 1.52(1.18,1.98) | 55.94(39.85,76.37) | 0.91(0.65,1.24) | -1.98(-2.26,-1.70) |
| Central African Republic | 11.61(3.30,19.86) | 0.95(0.27,1.62) | 14.95(7.16,23.55) | 0.65(0.31,1.03) | -0.93(-1.09,-0.78) |
| Chad | 36.58(22.05,55.54) | 1.25(0.75,1.90) | 109.33(65.38,159.15) | 1.21(0.73,1.77) | 0.25(0.13,0.37) |
| Chile | 24.82(20.97,29.44) | 0.62(0.53,0.74) | 21.37(16.92,26.90) | 0.59(0.46,0.74) | 0.08(-0.32,0.49) |
| China | 3625.27(2990.85,4465.12) | 1.14(0.94,1.40) | 1985.21(1562.60,2570.22) | 0.76(0.60,0.99) | -1.17(-1.59,-0.75) |
| Colombia | 138.13(113.29,165.11) | 1.18(0.97,1.42) | 126.35(92.62,170.44) | 1.19(0.87,1.61) | 0.97(0.53,1.40) |
| Comoros | 4.01(2.09,5.69) | 1.89(0.98,2.68) | 3.72(2.31,5.71) | 1.55(0.96,2.38) | -0.69(-1.08,-0.29) |
| Congo | 7.68(3.15,11.86) | 0.73(0.30,1.13) | 8.66(5.80,12.44) | 0.45(0.30,0.64) | -1.49(-1.68,-1.30) |
| Cook Islands | 0.06(0.03,0.09) | 0.88(0.50,1.40) | 0.09(0.04,0.18) | 2.34(1.06,4.78) | 1.48(0.80,2.17) |
| Costa Rica | 13.66(10.75,16.94) | 1.22(0.96,1.51) | 16.09(11.74,21.98) | 1.58(1.15,2.16) | 0.94(0.77,1.11) |
| Croatia | 15.80(11.49,21.08) | 1.60(1.16,2.14) | 11.10(7.56,15.86) | 1.86(1.27,2.66) | 1.24(0.60,1.88) |
| Cuba | 47.67(39.20,58.22) | 1.90(1.57,2.33) | 30.75(22.20,41.83) | 1.73(1.25,2.35) | -0.11(-0.41,0.21) |
| Cyprus | 5.35(3.61,7.93) | 2.70(1.83,4.01) | 6.02(3.97,8.78) | 2.75(1.82,4.01) | 1.44(0.29,2.60) |
| Czechia | 23.35(17.84,29.90) | 1.06(0.81,1.36) | 23.17(15.68,32.42) | 1.35(0.91,1.89) | 1.12(0.77,1.46) |
| Côte d'Ivoire | 93.05(64.80,130.08) | 1.63(1.14,2.28) | 196.86(123.69,351.71) | 1.70(1.07,3.04) | 0.41(0.17,0.65) |
| Democratic People's Republic of Korea | 32.63(20.45,47.92) | 0.55(0.34,0.81) | 18.24(11.85,29.76) | 0.38(0.25,0.62) | -0.83(-1.10,-0.56) |
| Democratic Republic of the Congo | 143.46(46.44,215.19) | 0.81(0.26,1.22) | 138.01(83.42,197.12) | 0.36(0.22,0.52) | -2.13(-2.35,-1.90) |
| Denmark | 11.05(8.85,14.06) | 1.25(1.00,1.59) | 12.71(9.08,17.87) | 1.33(0.95,1.87) | 0.04(-0.29,0.37) |
| Djibouti | 3.13(2.09,4.51) | 1.80(1.20,2.59) | 5.14(2.94,8.88) | 1.24(0.71,2.15) | -1.11(-1.47,-0.74) |
| Dominica | 0.35(0.26,0.47) | 1.40(1.04,1.88) | 0.43(0.30,0.61) | 3.13(2.18,4.43) | 2.78(2.56,3.00) |
| Dominican Republic | 31.29(22.67,40.82) | 1.16(0.84,1.51) | 24.61(15.26,37.43) | 0.84(0.52,1.27) | -1.46(-1.85,-1.08) |
| Ecuador | 27.78(23.46,32.86) | 0.72(0.61,0.85) | 42.93(31.63,56.72) | 0.85(0.62,1.12) | 0.61(-0.07,1.30) |
| Egypt | 160.11(116.11,262.30) | 0.72(0.52,1.18) | 146.69(86.40,330.09) | 0.40(0.23,0.90) | -1.91(-2.49,-1.32) |
| El Salvador | 12.68(9.80,16.17) | 0.59(0.45,0.75) | 12.17(8.79,16.44) | 0.67(0.48,0.90) | 0.72(0.50,0.93) |
| Equatorial Guinea | 1.65(0.55,2.66) | 0.84(0.28,1.35) | 3.56(1.67,6.85) | 0.61(0.29,1.17) | -1.65(-2.07,-1.22) |
| Eritrea | 32.74(21.29,45.86) | 2.06(1.34,2.88) | 35.33(20.93,63.18) | 1.40(0.83,2.50) | -1.18(-1.35,-1.01) |
| Estonia | 31.04(20.93,44.07) | 8.89(5.99,12.62) | 44.62(28.91,66.19) | 20.64(13.38,30.62) | 2.94(2.11,3.78) |
| Eswatini | 2.66(1.55,4.11) | 0.69(0.40,1.07) | 3.64(2.23,5.55) | 0.88(0.54,1.35) | 1.05(0.69,1.42) |
| Ethiopia | 704.39(185.39,1084.71) | 2.89(0.76,4.45) | 656.80(434.34,950.60) | 1.48(0.98,2.14) | -2.53(-2.87,-2.19) |
| Fiji | 1.86(1.16,2.92) | 0.66(0.41,1.04) | 4.15(2.41,6.75) | 1.52(0.89,2.48) | 2.81(2.60,3.01) |
| Finland | 13.80(10.51,17.64) | 1.43(1.09,1.83) | 19.00(12.91,26.59) | 2.24(1.52,3.14) | 0.90(0.26,1.54) |
| France | 222.89(172.13,291.07) | 1.90(1.47,2.48) | 285.87(203.89,388.93) | 2.46(1.76,3.35) | 0.57(0.29,0.84) |
| Gabon | 2.40(1.36,3.57) | 0.59(0.33,0.88) | 4.02(2.48,6.33) | 0.63(0.39,0.99) | 0.73(0.48,0.99) |
| Gambia | 8.60(5.64,12.06) | 1.86(1.22,2.61) | 12.95(7.68,23.16) | 1.30(0.77,2.33) | -1.35(-1.64,-1.07) |
| Georgia | 26.31(18.03,36.49) | 1.92(1.32,2.67) | 5.01(3.37,7.15) | 0.68(0.46,0.97) | -3.44(-4.17,-2.71) |
| Germany | 220.97(170.43,276.26) | 1.71(1.32,2.13) | 176.14(133.96,233.32) | 1.47(1.12,1.95) | -0.50(-0.84,-0.16) |
| Ghana | 153.90(74.86,231.85) | 2.29(1.11,3.45) | 78.53(48.70,135.01) | 0.61(0.38,1.05) | -5.84(-6.91,-4.76) |
| Greece | 21.78(16.35,29.52) | 1.08(0.81,1.46) | 13.24(10.16,17.36) | 0.95(0.73,1.24) | -0.15(-0.67,0.36) |
| Greenland | 0.18(0.08,0.25) | 1.24(0.58,1.76) | 0.07(0.04,0.10) | 0.59(0.38,0.87) | -2.13(-2.63,-1.64) |
| Grenada | 0.83(0.65,1.08) | 2.48(1.93,3.24) | 0.62(0.47,0.84) | 2.84(2.16,3.86) | 0.75(0.28,1.23) |
| Guam | 1.11(0.78,1.54) | 2.67(1.86,3.69) | 0.84(0.50,1.37) | 2.31(1.37,3.73) | 2.38(1.31,3.47) |
| Guatemala | 21.68(19.14,24.89) | 0.53(0.47,0.61) | 16.44(12.92,21.49) | 0.33(0.26,0.44) | -0.59(-0.86,-0.32) |
| Guinea | 58.01(38.43,82.15) | 2.11(1.40,2.99) | 64.37(37.92,131.99) | 1.06(0.63,2.18) | -1.69(-1.85,-1.53) |
| Guinea-Bissau | 9.28(5.66,13.62) | 1.92(1.17,2.82) | 8.39(5.16,13.16) | 0.93(0.57,1.47) | -2.09(-2.42,-1.75) |
| Guyana | 1.98(1.52,2.46) | 0.67(0.52,0.84) | 1.60(1.14,2.14) | 0.75(0.54,1.00) | 1.68(1.15,2.22) |
| Haiti | 92.30(25.56,146.31) | 3.40(0.94,5.39) | 95.68(39.14,157.11) | 2.20(0.90,3.61) | -0.99(-1.21,-0.76) |
| Honduras | 9.47(7.11,12.21) | 0.43(0.32,0.55) | 5.97(3.11,10.34) | 0.18(0.09,0.32) | -2.97(-3.29,-2.65) |
| Hungary | 23.54(18.33,29.98) | 1.10(0.86,1.41) | 16.23(12.04,22.41) | 1.17(0.87,1.61) | 0.91(0.48,1.35) |
| Iceland | 3.50(2.51,4.75) | 5.52(3.96,7.48) | 4.93(3.22,7.20) | 7.29(4.77,10.66) | 1.31(0.38,2.25) |
| India | 2788.41(1697.94,3755.23) | 0.85(0.52,1.15) | 1895.15(1460.53,2495.08) | 0.52(0.40,0.68) | -1.59(-1.79,-1.39) |
| Indonesia | 394.24(217.08,597.31) | 0.58(0.32,0.88) | 321.73(226.23,503.68) | 0.48(0.34,0.75) | -0.60(-0.66,-0.54) |
| Iran (Islamic Republic of) | 214.75(150.86,292.88) | 0.85(0.59,1.15) | 260.70(176.88,368.55) | 1.29(0.88,1.83) | 1.89(1.38,2.41) |
| Iraq | 132.20(79.67,197.54) | 1.61(0.97,2.40) | 179.03(117.62,255.38) | 1.33(0.87,1.90) | 0.11(-0.19,0.42) |
| Ireland | 19.48(14.01,26.86) | 1.98(1.43,2.73) | 19.45(13.30,28.26) | 1.95(1.33,2.83) | 0.43(-0.08,0.94) |
| Israel | 35.61(27.60,46.42) | 2.32(1.80,3.03) | 60.09(44.73,81.77) | 2.29(1.70,3.11) | 0.29(-0.32,0.91) |
| Italy | 328.58(236.57,441.71) | 3.56(2.56,4.79) | 233.12(173.69,313.21) | 3.07(2.29,4.12) | -1.14(-1.63,-0.65) |
| Jamaica | 21.09(16.82,26.55) | 2.52(2.01,3.18) | 10.68(7.73,15.10) | 1.83(1.32,2.59) | -1.15(-1.35,-0.94) |
| Japan | 241.22(203.13,281.50) | 1.04(0.88,1.22) | 179.31(141.66,225.05) | 1.16(0.92,1.46) | 0.17(-0.05,0.39) |
| Jordan | 24.39(17.05,33.82) | 1.49(1.04,2.07) | 92.96(63.05,133.87) | 2.56(1.74,3.68) | 1.88(1.49,2.27) |
| Kazakhstan | 78.81(63.83,97.87) | 1.52(1.23,1.88) | 41.73(30.43,56.78) | 0.77(0.56,1.05) | -1.93(-2.58,-1.28) |
| Kenya | 82.79(53.52,118.46) | 0.74(0.48,1.06) | 104.02(72.91,137.60) | 0.56(0.39,0.74) | 0.08(-0.44,0.61) |
| Kiribati | 0.08(0.04,0.11) | 0.26(0.14,0.38) | 0.12(0.06,0.20) | 0.28(0.13,0.48) | -0.03(-0.31,0.24) |
| Kuwait | 17.80(13.61,22.87) | 3.21(2.45,4.12) | 19.88(13.57,27.38) | 2.35(1.60,3.24) | 1.13(0.12,2.16) |
| Kyrgyzstan | 11.88(8.85,16.26) | 0.71(0.53,0.97) | 13.93(9.80,19.55) | 0.61(0.43,0.86) | -0.38(-0.96,0.19) |
| Lao People's Democratic Republic | 15.58(5.54,24.51) | 0.85(0.30,1.33) | 12.34(7.83,20.14) | 0.54(0.34,0.88) | -1.52(-1.59,-1.45) |
| Latvia | 2.59(2.12,3.13) | 0.45(0.37,0.55) | 1.69(1.25,2.21) | 0.57(0.42,0.75) | 2.46(1.76,3.17) |
| Lebanon | 16.07(10.83,22.39) | 1.54(1.04,2.14) | 26.85(16.57,41.69) | 2.10(1.30,3.26) | 1.09(0.65,1.54) |
| Lesotho | 2.51(1.67,4.04) | 0.37(0.24,0.59) | 4.44(2.71,6.72) | 0.70(0.43,1.07) | 3.02(2.64,3.40) |
| Liberia | 24.77(13.63,34.36) | 2.19(1.21,3.04) | 26.50(16.55,38.76) | 1.21(0.76,1.77) | -1.72(-2.47,-0.97) |
| Libya | 30.75(20.56,42.93) | 1.70(1.14,2.37) | 53.40(34.83,76.46) | 3.58(2.33,5.13) | 3.24(2.81,3.66) |
| Lithuania | 5.08(3.80,6.78) | 0.61(0.46,0.82) | 5.37(3.83,7.56) | 1.32(0.94,1.85) | 1.95(1.34,2.56) |
| Luxembourg | 1.14(0.85,1.53) | 1.73(1.29,2.32) | 1.87(1.39,2.53) | 1.85(1.38,2.50) | -1.31(-1.84,-0.78) |
| Madagascar | 107.69(76.19,144.87) | 1.97(1.40,2.66) | 125.16(79.42,187.59) | 1.07(0.68,1.60) | -1.56(-1.77,-1.36) |
| Malawi | 349.37(249.76,466.12) | 7.68(5.49,10.25) | 379.63(180.31,782.00) | 4.67(2.22,9.63) | -1.26(-1.52,-1.00) |
| Malaysia | 33.82(23.67,45.77) | 0.51(0.36,0.70) | 42.65(28.47,60.77) | 0.56(0.37,0.80) | 0.75(0.42,1.09) |
| Maldives | 0.59(0.28,0.87) | 0.56(0.27,0.83) | 0.57(0.36,0.86) | 0.57(0.36,0.86) | 0.72(0.42,1.01) |
| Mali | 56.29(35.72,82.05) | 1.36(0.86,1.99) | 76.77(44.87,152.13) | 0.66(0.39,1.31) | -2.03(-2.20,-1.85) |
| Malta | 3.65(2.31,5.28) | 4.17(2.64,6.03) | 9.14(5.75,13.44) | 14.27(8.98,20.99) | 2.02(1.17,2.88) |
| Marshall Islands | 0.09(0.06,0.14) | 0.41(0.26,0.65) | 0.18(0.10,0.31) | 1.01(0.58,1.79) | 2.82(2.45,3.18) |
| Mauritania | 8.76(5.80,12.78) | 0.95(0.63,1.38) | 17.54(10.90,25.58) | 0.95(0.59,1.38) | -0.29(-0.50,-0.07) |
| Mauritius | 0.45(0.39,0.52) | 0.14(0.12,0.16) | 0.88(0.68,1.13) | 0.43(0.33,0.54) | 2.22(1.48,2.96) |
| Mexico | 283.66(256.75,314.24) | 0.85(0.77,0.94) | 203.61(171.45,239.91) | 0.63(0.53,0.75) | -0.56(-1.04,-0.08) |
| Micronesia (Federated States of) | 0.31(0.19,0.50) | 0.68(0.40,1.10) | 0.32(0.18,0.54) | 1.04(0.58,1.77) | 1.50(1.28,1.72) |
| Monaco | 0.10(0.06,0.16) | 2.81(1.69,4.43) | 0.20(0.13,0.32) | 4.04(2.57,6.39) | -0.22(-0.75,0.32) |
| Mongolia | 12.93(8.31,18.07) | 1.44(0.92,2.01) | 7.18(4.82,10.35) | 0.66(0.44,0.95) | -2.51(-3.14,-1.89) |
| Montenegro | 2.81(2.05,3.65) | 1.74(1.27,2.26) | 1.04(0.68,1.59) | 0.94(0.61,1.43) | -1.35(-1.96,-0.74) |
| Morocco | 90.40(57.19,131.32) | 0.92(0.58,1.34) | 70.00(44.09,103.69) | 0.71(0.45,1.06) | -0.46(-0.63,-0.29) |
| Mozambique | 25.01(13.42,63.71) | 0.40(0.22,1.03) | 25.96(12.48,59.62) | 0.18(0.09,0.42) | -2.14(-2.44,-1.84) |
| Myanmar | 148.31(51.56,230.62) | 1.00(0.35,1.56) | 105.97(66.91,163.94) | 0.68(0.43,1.05) | -1.54(-1.70,-1.38) |
| Namibia | 4.60(2.88,6.82) | 0.77(0.48,1.13) | 11.17(6.73,17.28) | 1.35(0.82,2.09) | 2.86(2.17,3.55) |
| Nauru | 0.05(0.03,0.07) | 1.07(0.66,1.62) | 0.08(0.05,0.13) | 2.03(1.17,3.24) | 1.80(1.59,2.02) |
| Nepal | 98.87(44.42,154.17) | 1.17(0.53,1.83) | 65.13(40.88,107.43) | 0.71(0.44,1.16) | -1.35(-1.57,-1.13) |
| Netherlands | 39.28(31.33,48.58) | 1.44(1.15,1.78) | 41.11(31.48,54.45) | 1.53(1.17,2.03) | 0.42(0.01,0.84) |
| New Zealand | 16.01(12.17,20.56) | 2.00(1.52,2.57) | 21.75(16.41,28.78) | 2.22(1.67,2.93) | -0.47(-1.63,0.71) |
| Nicaragua | 15.05(11.08,19.12) | 0.83(0.61,1.05) | 8.44(6.10,11.71) | 0.43(0.31,0.59) | -1.02(-1.39,-0.66) |
| Niger | 89.23(50.21,134.30) | 2.20(1.24,3.31) | 108.05(60.63,166.79) | 0.85(0.48,1.31) | -3.09(-3.34,-2.84) |
| Nigeria | 854.62(549.17,1208.59) | 2.18(1.40,3.09) | 1977.44(977.58,3011.15) | 1.95(0.96,2.96) | -0.07(-0.22,0.08) |
| Niue | 0.01(0.01,0.01) | 1.14(0.70,1.77) | 0.06(0.04,0.09) | 14.78(9.19,24.50) | 4.53(3.16,5.91) |
| North Macedonia | 5.49(4.13,7.31) | 1.04(0.78,1.39) | 3.29(2.06,4.77) | 1.01(0.63,1.46) | 0.96(0.42,1.50) |
| Northern Mariana Islands | 0.07(0.04,0.14) | 0.60(0.33,1.14) | 0.16(0.09,0.28) | 1.43(0.80,2.51) | 3.56(2.72,4.40) |
| Norway | 15.76(12.20,20.19) | 1.97(1.53,2.53) | 13.78(10.24,18.71) | 1.49(1.11,2.03) | -0.58(-1.40,0.24) |
| Oman | 8.39(5.06,12.49) | 1.00(0.60,1.49) | 16.62(11.29,23.92) | 1.36(0.92,1.96) | 2.01(1.53,2.50) |
| Pakistan | 784.56(559.80,1049.17) | 1.59(1.14,2.13) | 1990.97(1340.98,2880.86) | 2.33(1.57,3.37) | 1.70(1.51,1.88) |
| Palau | 0.03(0.02,0.04) | 0.58(0.34,0.91) | 0.02(0.01,0.03) | 0.64(0.36,1.05) | 0.27(0.01,0.53) |
| Palestine | 7.01(4.67,10.17) | 0.72(0.48,1.05) | 16.97(11.60,23.88) | 0.91(0.62,1.28) | 1.44(0.94,1.94) |
| Panama | 8.10(6.58,9.98) | 0.97(0.79,1.20) | 18.22(13.91,23.55) | 1.58(1.21,2.04) | 1.36(1.11,1.62) |
| Papua New Guinea | 14.89(7.62,23.80) | 0.88(0.45,1.40) | 48.35(26.49,78.30) | 1.23(0.68,2.00) | 1.06(0.83,1.28) |
| Paraguay | 14.70(10.47,19.95) | 0.88(0.63,1.19) | 19.90(13.43,29.55) | 0.99(0.67,1.47) | 0.63(0.18,1.08) |
| Peru | 99.61(78.71,133.54) | 1.20(0.95,1.61) | 140.63(95.43,202.62) | 1.47(1.00,2.12) | 1.13(0.90,1.35) |
| Philippines | 199.85(133.81,256.85) | 0.79(0.53,1.02) | 201.13(153.09,271.56) | 0.59(0.45,0.80) | -0.61(-0.76,-0.46) |
| Poland | 81.22(71.74,92.21) | 0.85(0.75,0.96) | 56.49(44.51,72.78) | 0.96(0.76,1.24) | 0.46(-0.20,1.14) |
| Portugal | 57.55(43.12,76.38) | 2.72(2.04,3.61) | 33.12(22.74,46.83) | 2.43(1.67,3.44) | -1.32(-1.74,-0.89) |
| Puerto Rico | 15.00(12.08,18.51) | 1.51(1.21,1.86) | 4.17(3.19,5.51) | 0.94(0.72,1.24) | -0.77(-1.13,-0.42) |
| Qatar | 0.66(0.44,0.98) | 0.53(0.35,0.79) | 4.78(3.06,7.38) | 0.97(0.62,1.49) | 3.01(2.51,3.51) |
| Republic of Korea | 81.54(62.56,112.23) | 0.72(0.55,0.99) | 48.57(31.47,72.73) | 0.80(0.52,1.20) | 0.58(0.16,1.01) |
| Republic of Moldova | 50.79(37.57,68.57) | 4.11(3.04,5.55) | 13.42(9.77,18.10) | 2.57(1.87,3.46) | -0.64(-1.33,0.06) |
| Romania | 69.44(57.70,85.92) | 1.25(1.04,1.54) | 28.66(22.16,36.88) | 0.95(0.74,1.23) | -0.77(-1.18,-0.35) |
| Russian Federation | 868.37(827.23,911.97) | 2.50(2.38,2.63) | 277.92(254.93,301.10) | 1.07(0.98,1.15) | -2.88(-3.65,-2.11) |
| Rwanda | 111.06(74.57,156.08) | 3.27(2.20,4.60) | 73.81(47.61,126.92) | 1.48(0.96,2.55) | -3.07(-3.36,-2.78) |
| Saint Kitts and Nevis | 0.17(0.14,0.20) | 1.21(1.02,1.44) | 0.16(0.12,0.21) | 1.59(1.22,2.09) | 1.17(0.84,1.50) |
| Saint Lucia | 0.79(0.64,0.98) | 1.53(1.25,1.91) | 0.60(0.44,0.81) | 2.01(1.47,2.74) | 0.32(-0.11,0.74) |
| Saint Vincent and the Grenadines | 0.97(0.79,1.20) | 2.37(1.92,2.93) | 0.60(0.45,0.77) | 2.41(1.81,3.10) | 0.18(-0.22,0.58) |
| Samoa | 1.43(0.89,2.13) | 2.00(1.25,2.99) | 1.80(1.00,3.21) | 2.26(1.25,4.01) | 0.18(-0.03,0.38) |
| San Marino | 0.37(0.24,0.54) | 9.14(5.93,13.17) | 0.20(0.13,0.31) | 4.60(2.94,7.07) | -1.97(-2.17,-1.77) |
| Sao Tome and Principe | 0.95(0.66,1.39) | 1.67(1.16,2.46) | 0.61(0.33,1.14) | 0.79(0.43,1.46) | -2.09(-2.61,-1.57) |
| Saudi Arabia | 57.81(33.09,82.95) | 0.88(0.50,1.27) | 61.19(35.33,96.49) | 0.81(0.47,1.28) | -0.07(-0.25,0.10) |
| Senegal | 57.84(39.87,82.58) | 1.58(1.09,2.26) | 52.56(34.41,83.90) | 0.83(0.54,1.32) | -2.15(-2.39,-1.90) |
| Serbia | 37.46(24.00,52.50) | 1.73(1.11,2.42) | 10.27(6.61,15.45) | 0.77(0.50,1.16) | -3.00(-3.49,-2.50) |
| Seychelles | 0.08(0.06,0.11) | 0.33(0.24,0.47) | 0.08(0.05,0.12) | 0.35(0.23,0.52) | 1.91(0.83,3.00) |
| Sierra Leone | 37.60(21.75,54.11) | 2.07(1.20,2.98) | 42.61(28.01,62.09) | 1.19(0.78,1.74) | -1.75(-1.92,-1.58) |
| Singapore | 4.98(4.02,6.20) | 0.77(0.62,0.96) | 8.28(6.04,11.15) | 1.02(0.74,1.37) | 1.70(1.13,2.28) |
| Slovakia | 12.59(9.13,16.99) | 0.95(0.69,1.28) | 10.15(6.90,14.81) | 1.18(0.81,1.73) | 0.76(0.35,1.19) |
| Slovenia | 5.95(4.42,7.60) | 1.44(1.07,1.84) | 7.04(4.84,9.70) | 2.25(1.55,3.11) | 2.66(2.15,3.18) |
| Solomon Islands | 0.65(0.34,1.05) | 0.42(0.22,0.67) | 2.00(1.25,3.17) | 0.77(0.48,1.22) | 1.96(1.63,2.30) |
| Somalia | 73.19(38.98,109.21) | 1.88(1.00,2.80) | 102.81(50.31,160.81) | 1.00(0.49,1.56) | -1.59(-1.98,-1.21) |
| South Africa | 78.61(52.87,119.32) | 0.58(0.39,0.88) | 121.51(98.29,154.62) | 0.80(0.65,1.02) | 0.83(0.34,1.32) |
| South Sudan | 66.29(37.63,94.24) | 2.53(1.43,3.59) | 105.67(63.15,154.01) | 2.46(1.47,3.59) | 0.41(-0.30,1.13) |
| Spain | 140.51(108.87,173.81) | 1.79(1.39,2.22) | 142.17(101.80,195.47) | 2.19(1.57,3.02) | 0.33(-0.12,0.78) |
| Sri Lanka | 24.69(17.69,32.61) | 0.45(0.32,0.59) | 23.47(14.76,34.15) | 0.46(0.29,0.67) | 0.20(-0.42,0.83) |
| Sudan | 135.90(55.23,275.55) | 1.53(0.62,3.10) | 186.65(120.81,274.39) | 1.13(0.73,1.65) | -0.76(-0.87,-0.65) |
| Suriname | 1.57(0.94,2.08) | 1.20(0.72,1.60) | 1.69(1.14,2.47) | 1.18(0.79,1.72) | 0.21(-0.10,0.52) |
| Sweden | 20.79(16.49,26.05) | 1.35(1.07,1.69) | 20.65(15.87,26.76) | 1.13(0.87,1.47) | 0.42(-0.46,1.31) |
| Switzerland | 39.94(30.01,52.97) | 3.46(2.60,4.58) | 39.37(27.84,54.03) | 2.95(2.09,4.05) | -1.10(-1.78,-0.42) |
| Syrian Arab Republic | 47.43(32.26,62.42) | 0.80(0.54,1.05) | 37.56(24.55,56.61) | 1.03(0.67,1.55) | 0.98(0.45,1.52) |
| Taiwan (Province of China) | 43.89(37.67,51.51) | 0.80(0.68,0.94) | 25.00(19.01,31.84) | 0.85(0.65,1.08) | 0.15(-0.32,0.62) |
| Tajikistan | 51.72(23.79,75.88) | 2.23(1.02,3.27) | 60.09(38.72,89.36) | 1.68(1.08,2.49) | -1.66(-2.14,-1.18) |
| Thailand | 147.48(109.46,201.05) | 0.87(0.65,1.19) | 89.22(59.38,139.15) | 0.91(0.61,1.42) | -0.53(-0.94,-0.11) |
| Timor-Leste | 2.41(0.91,3.86) | 0.73(0.27,1.16) | 2.17(1.46,3.45) | 0.42(0.28,0.66) | -1.86(-2.24,-1.47) |
| Togo | 21.66(14.50,31.09) | 1.23(0.82,1.76) | 29.37(17.93,44.54) | 0.89(0.54,1.35) | -0.98(-1.11,-0.86) |
| Tokelau | 0.01(0.00,0.01) | 0.88(0.52,1.39) | 0.05(0.02,0.08) | 11.71(6.37,20.53) | 4.06(2.24,5.90) |
| Tonga | 1.17(0.71,1.79) | 2.79(1.70,4.28) | 1.81(1.02,3.20) | 4.63(2.63,8.21) | 1.02(0.55,1.50) |
| Trinidad and Tobago | 6.08(4.98,7.38) | 1.50(1.23,1.82) | 3.36(2.49,4.43) | 1.23(0.91,1.63) | 0.01(-0.40,0.41) |
| Tunisia | 69.44(46.52,96.78) | 2.24(1.50,3.12) | 55.26(35.45,82.55) | 2.00(1.28,2.98) | -0.04(-0.20,0.11) |
| Turkey | 7.14(5.79,8.69) | 0.48(0.39,0.58) | 7.80(5.87,10.15) | 0.51(0.39,0.67) | -0.31(-0.83,0.22) |
| Turkmenistan | 0.03(0.02,0.05) | 0.88(0.47,1.37) | 0.04(0.02,0.06) | 1.05(0.66,1.54) | 0.72(0.60,0.83) |
| Tuvalu | 267.91(156.91,374.04) | 1.31(0.77,1.83) | 261.01(185.20,366.13) | 1.41(1.00,1.98) | 0.64(0.25,1.04) |
| Uganda | 249.14(164.03,349.15) | 2.96(1.95,4.15) | 608.41(344.59,1027.66) | 3.07(1.74,5.18) | 0.30(0.07,0.53) |
| Ukraine | 119.62(78.00,164.20) | 1.05(0.69,1.44) | 54.57(41.65,71.98) | 0.86(0.66,1.13) | -0.94(-1.45,-0.43) |
| United Arab Emirates | 3.34(2.02,5.39) | 0.57(0.34,0.91) | 6.08(4.02,8.93) | 0.45(0.30,0.67) | -0.36(-0.78,0.06) |
| United Kingdom | 242.48(216.23,272.24) | 2.22(1.98,2.49) | 215.84(185.70,250.17) | 1.83(1.58,2.12) | -0.41(-0.80,-0.01) |
| United Republic of Tanzania | 331.92(225.91,448.40) | 2.75(1.87,3.71) | 480.01(300.14,788.12) | 1.97(1.23,3.23) | -0.56(-0.85,-0.28) |
| United States Virgin Islands | 0.44(0.31,0.62) | 1.37(0.98,1.93) | 0.08(0.04,0.13) | 0.58(0.33,0.98) | -1.76(-2.33,-1.19) |
| United States of America | 884.86(835.25,940.29) | 1.58(1.49,1.68) | 627.82(555.57,701.47) | 1.06(0.93,1.18) | -1.38(-1.54,-1.22) |
| Uruguay | 8.84(7.22,11.08) | 1.08(0.88,1.35) | 7.13(5.35,9.37) | 1.08(0.81,1.42) | -0.44(-1.08,0.21) |
| Uzbekistan | 105.34(71.85,145.07) | 1.23(0.84,1.70) | 127.83(92.69,173.90) | 1.27(0.92,1.72) | 0.42(0.18,0.66) |
| Vanuatu | 0.27(0.16,0.43) | 0.40(0.23,0.64) | 0.77(0.48,1.20) | 0.66(0.41,1.03) | 1.78(1.36,2.20) |
| Venezuela (Bolivarian Republic of) | 63.61(54.07,75.49) | 0.90(0.76,1.06) | 77.99(57.87,104.13) | 1.18(0.87,1.57) | 1.09(0.68,1.50) |
| Viet Nam | 178.56(121.26,246.25) | 0.67(0.46,0.93) | 241.76(155.21,352.55) | 0.98(0.63,1.42) | 1.50(1.39,1.60) |
| Yemen | 48.12(25.84,91.06) | 0.68(0.36,1.28) | 72.68(44.49,114.50) | 0.53(0.32,0.83) | -0.57(-0.73,-0.41) |
| Zambia | 108.66(73.45,146.60) | 2.89(1.96,3.90) | 139.38(80.67,236.09) | 1.69(0.98,2.85) | -1.58(-1.75,-1.42) |
| Zimbabwe | 33.63(15.91,49.04) | 0.70(0.33,1.02) | 103.23(47.74,150.59) | 1.64(0.76,2.39) | 4.95(3.83,6.08) |
| ^a^EAPC is expressed as 95% CIs | | | | | |

**Table S3** Death of Hodgkin Lymphoma in Children Between 1990 and 2021 at 204 Countries and Territories.

| Location | Rate per 100000 (95% UI) | | | | |
| --- | --- | --- | --- | --- | --- |
|  | 1990 | | 2021 | | EAPC^a^ |
|  | Cases | Rates | Cases | Rates |  |
| Global | 2645.56(1483.97,3394.11) | 0.15(0.09,0.20) | 1918.70(1051.84,2587.73) | 0.10(0.05,0.13) | -1.38(-1.45,-1.31) |
| 5 SDI | | | | | |
| High SDI | 54.28(47.79,60.43) | 0.03(0.03,0.03) | 11.37(9.50,13.42) | 0.01(0.01,0.01) | -4.80(-4.85,-4.75) |
| High-middle SDI | 260.54(173.11,321.42) | 0.10(0.06,0.12) | 45.76(35.33,59.99) | 0.02(0.02,0.03) | -5.19(-5.35,-5.03) |
| Middle SDI | 659.38(364.63,838.11) | 0.11(0.06,0.15) | 234.04(132.67,304.64) | 0.04(0.02,0.05) | -3.11(-3.23,-2.99) |
| Low-middle SDI | 940.14(510.70,1294.67) | 0.20(0.11,0.27) | 696.00(419.67,1007.00) | 0.12(0.07,0.17) | -1.49(-1.58,-1.41) |
| Low SDI | 730.02(353.74,1068.90) | 0.32(0.15,0.47) | 930.81(417.76,1340.70) | 0.20(0.09,0.29) | -1.48(-1.57,-1.39) |
| 21 GBD Region | | | | | |
| Andean Latin America | 14.09(9.59,19.27) | 0.09(0.06,0.13) | 6.96(4.75,10.68) | 0.04(0.03,0.06) | -2.74(-2.95,-2.52) |
| Australasia | 1.18(0.97,1.43) | 0.03(0.02,0.03) | 0.32(0.24,0.43) | 0.01(0.00,0.01) | -4.54(-5.20,-3.87) |
| Caribbean | 6.74(4.94,9.01) | 0.06(0.04,0.08) | 4.04(2.50,6.04) | 0.04(0.02,0.05) | -1.62(-1.74,-1.49) |
| Central Asia | 29.25(23.57,34.69) | 0.12(0.09,0.14) | 15.92(12.84,20.19) | 0.06(0.05,0.07) | -2.32(-2.68,-1.95) |
| Central Europe | 29.39(25.77,32.90) | 0.10(0.09,0.11) | 2.56(2.16,3.25) | 0.01(0.01,0.02) | -6.47(-6.77,-6.17) |
| Central Latin America | 109.12(102.26,116.97) | 0.17(0.16,0.18) | 28.00(24.45,32.30) | 0.04(0.04,0.05) | -3.83(-4.09,-3.56) |
| Central Sub-Saharan Africa | 11.19(6.12,20.33) | 0.04(0.02,0.08) | 16.36(10.64,26.48) | 0.03(0.02,0.05) | -1.22(-1.35,-1.09) |
| East Asia | 345.91(128.09,500.10) | 0.10(0.04,0.15) | 35.83(23.28,57.13) | 0.01(0.01,0.02) | -7.14(-7.40,-6.88) |
| Eastern Europe | 62.11(58.38,66.23) | 0.12(0.11,0.13) | 9.21(8.46,10.31) | 0.03(0.02,0.03) | -5.20(-5.42,-4.98) |
| Eastern Sub-Saharan Africa | 361.31(228.97,523.39) | 0.40(0.25,0.58) | 434.98(220.48,700.76) | 0.24(0.12,0.39) | -1.50(-1.59,-1.41) |
| High-income Asia Pacific | 2.07(1.67,2.79) | 0.01(0.00,0.01) | 0.46(0.41,0.55) | 0.00(0.00,0.00) | -3.06(-3.34,-2.78) |
| High-income North America | 13.40(12.92,13.95) | 0.02(0.02,0.02) | 3.47(3.24,3.70) | 0.01(0.00,0.01) | -4.75(-4.91,-4.59) |
| North Africa and Middle East | 160.73(97.17,220.76) | 0.11(0.07,0.16) | 98.98(60.23,139.21) | 0.05(0.03,0.08) | -2.13(-2.34,-1.92) |
| Oceania | 0.48(0.23,0.73) | 0.02(0.01,0.03) | 0.67(0.25,1.12) | 0.01(0.01,0.02) | -0.68(-0.82,-0.54) |
| South Asia | 1052.52(540.63,1496.26) | 0.24(0.12,0.35) | 719.66(453.94,1087.83) | 0.14(0.09,0.21) | -1.63(-1.73,-1.52) |
| Southeast Asia | 64.39(43.08,99.28) | 0.04(0.03,0.06) | 28.03(19.23,51.41) | 0.02(0.01,0.03) | -2.76(-2.84,-2.67) |
| Southern Latin America | 8.33(6.62,10.69) | 0.06(0.04,0.07) | 3.05(2.35,3.95) | 0.02(0.02,0.03) | -2.80(-3.09,-2.51) |
| Southern Sub-Saharan Africa | 9.63(6.07,13.47) | 0.05(0.03,0.07) | 13.67(7.93,19.11) | 0.06(0.03,0.08) | 0.73(0.52,0.93) |
| Tropical Latin America | 46.26(40.61,52.39) | 0.09(0.08,0.10) | 12.33(10.24,14.49) | 0.02(0.02,0.03) | -3.35(-3.70,-2.99) |
| Western Europe | 25.68(24.00,27.68) | 0.04(0.03,0.04) | 6.05(5.41,6.89) | 0.01(0.01,0.01) | -4.26(-4.46,-4.06) |
| Western Sub-Saharan Africa | 291.79(56.20,498.77) | 0.33(0.06,0.57) | 478.14(100.70,868.91) | 0.22(0.05,0.40) | -1.38(-1.50,-1.25) |
| 204 countries and territories | | | | | |
| Afghanistan | 7.07(3.57,14.11) | 0.16(0.08,0.33) | 16.97(10.48,27.30) | 0.12(0.07,0.19) | -0.39(-0.80,0.03) |
| Albania | 1.21(0.49,1.98) | 0.11(0.04,0.18) | 0.12(0.07,0.21) | 0.03(0.01,0.05) | -3.90(-4.46,-3.34) |
| Algeria | 34.68(14.60,57.78) | 0.32(0.14,0.54) | 13.95(6.99,23.48) | 0.10(0.05,0.18) | -3.62(-3.78,-3.47) |
| American Samoa | 0.00(0.00,0.00) | 0.00(0.00,0.00) | 0.00(0.00,0.00) | 0.00(0.00,0.00) | 2.79(2.05,3.54) |
| Andorra | 0.00(0.00,0.00) | 0.02(0.01,0.03) | 0.00(0.00,0.00) | 0.01(0.00,0.01) | -2.74(-3.00,-2.49) |
| Angola | 2.59(1.15,5.32) | 0.05(0.02,0.11) | 4.10(2.32,6.95) | 0.03(0.02,0.05) | -2.20(-2.34,-2.06) |
| Antigua and Barbuda | 0.00(0.00,0.00) | 0.01(0.01,0.01) | 0.00(0.00,0.00) | 0.00(0.00,0.01) | -2.61(-3.89,-1.31) |
| Argentina | 6.99(5.53,9.00) | 0.07(0.05,0.09) | 2.34(1.79,3.03) | 0.02(0.02,0.03) | -3.18(-3.49,-2.86) |
| Armenia | 0.81(0.48,0.99) | 0.08(0.05,0.10) | 0.18(0.15,0.23) | 0.03(0.02,0.04) | -2.59(-2.90,-2.27) |
| Australia | 1.08(0.88,1.33) | 0.03(0.02,0.04) | 0.28(0.20,0.38) | 0.01(0.00,0.01) | -4.65(-5.27,-4.03) |
| Austria | 0.49(0.40,0.60) | 0.04(0.03,0.04) | 0.09(0.06,0.12) | 0.01(0.00,0.01) | -4.34(-4.74,-3.93) |
| Azerbaijan | 4.35(2.36,7.47) | 0.18(0.10,0.31) | 2.86(1.42,5.03) | 0.12(0.06,0.21) | -1.88(-2.45,-1.32) |
| Bahamas | 0.02(0.01,0.02) | 0.02(0.02,0.02) | 0.01(0.01,0.01) | 0.01(0.01,0.01) | -2.45(-3.10,-1.80) |
| Bahrain | 0.15(0.09,0.25) | 0.09(0.06,0.15) | 0.10(0.06,0.17) | 0.04(0.02,0.06) | -3.01(-3.35,-2.66) |
| Bangladesh | 171.04(88.19,317.83) | 0.35(0.18,0.65) | 70.76(36.77,150.22) | 0.15(0.08,0.33) | -2.43(-2.56,-2.31) |
| Barbados | 0.02(0.01,0.02) | 0.03(0.02,0.04) | 0.01(0.00,0.01) | 0.01(0.01,0.02) | -1.69(-2.34,-1.04) |
| Belarus | 2.62(1.84,3.90) | 0.11(0.08,0.16) | 0.41(0.28,0.60) | 0.03(0.02,0.04) | -4.65(-4.91,-4.40) |
| Belgium | 0.52(0.37,0.71) | 0.03(0.02,0.04) | 0.17(0.11,0.25) | 0.01(0.01,0.01) | -4.07(-4.71,-3.42) |
| Belize | 0.05(0.04,0.06) | 0.06(0.05,0.07) | 0.02(0.02,0.03) | 0.02(0.02,0.02) | -3.22(-3.51,-2.93) |
| Benin | 0.84(0.38,1.43) | 0.03(0.02,0.06) | 0.71(0.33,1.51) | 0.01(0.01,0.02) | -4.04(-4.33,-3.76) |
| Bermuda | 0.00(0.00,0.00) | 0.03(0.02,0.04) | 0.00(0.00,0.00) | 0.01(0.01,0.01) | -2.84(-3.14,-2.54) |
| Bhutan | 0.54(0.21,0.89) | 0.21(0.08,0.34) | 0.21(0.10,0.50) | 0.11(0.05,0.27) | -2.16(-2.49,-1.82) |
| Bolivia (Plurinational State of) | 5.60(2.85,9.48) | 0.21(0.11,0.35) | 2.50(1.27,4.71) | 0.07(0.04,0.14) | -3.59(-3.67,-3.51) |
| Bosnia and Herzegovina | 0.72(0.42,1.08) | 0.07(0.04,0.10) | 0.09(0.05,0.17) | 0.02(0.01,0.03) | -4.50(-4.82,-4.19) |
| Botswana | 0.34(0.20,0.57) | 0.06(0.03,0.10) | 0.30(0.17,0.55) | 0.04(0.02,0.08) | -0.74(-0.92,-0.56) |
| Brazil | 45.23(39.70,51.42) | 0.09(0.08,0.10) | 11.61(9.68,13.71) | 0.02(0.02,0.03) | -3.42(-3.80,-3.05) |
| Brunei Darussalam | 0.02(0.01,0.03) | 0.02(0.01,0.04) | 0.01(0.01,0.02) | 0.01(0.01,0.02) | -0.71(-1.19,-0.23) |
| Bulgaria | 2.34(1.89,2.81) | 0.13(0.11,0.16) | 0.22(0.18,0.28) | 0.02(0.02,0.03) | -6.05(-6.45,-5.65) |
| Burkina Faso | 1.62(0.73,2.99) | 0.03(0.02,0.06) | 1.42(0.62,3.10) | 0.01(0.01,0.03) | -3.31(-3.66,-2.97) |
| Burundi | 10.98(5.90,16.65) | 0.42(0.22,0.64) | 14.03(5.74,26.00) | 0.24(0.10,0.44) | -1.40(-1.59,-1.21) |
| Cabo Verde | 0.00(0.00,0.00) | 0.00(0.00,0.00) | 0.00(0.00,0.00) | 0.00(0.00,0.00) | -2.88(-3.47,-2.30) |
| Cambodia | 2.20(1.24,4.08) | 0.05(0.03,0.09) | 0.93(0.40,2.21) | 0.02(0.01,0.04) | -3.37(-3.54,-3.19) |
| Cameroon | 1.53(0.71,2.58) | 0.03(0.01,0.05) | 1.50(0.74,3.01) | 0.01(0.01,0.02) | -3.91(-4.25,-3.57) |
| Canada | 2.13(1.76,2.60) | 0.04(0.03,0.05) | 0.28(0.20,0.37) | 0.00(0.00,0.01) | -7.48(-7.92,-7.03) |
| Central African Republic | 0.72(0.34,1.43) | 0.06(0.03,0.12) | 1.07(0.53,1.80) | 0.05(0.02,0.08) | -0.71(-0.84,-0.58) |
| Chad | 1.00(0.45,1.70) | 0.03(0.02,0.06) | 1.48(0.69,3.13) | 0.02(0.01,0.03) | -2.73(-3.01,-2.45) |
| Chile | 0.88(0.68,1.15) | 0.02(0.02,0.03) | 0.57(0.44,0.76) | 0.02(0.01,0.02) | -0.71(-1.76,0.36) |
| China | 342.71(125.88,496.47) | 0.11(0.04,0.16) | 34.96(22.71,55.41) | 0.01(0.01,0.02) | -7.21(-7.48,-6.95) |
| Colombia | 16.14(13.49,19.50) | 0.14(0.12,0.17) | 4.91(3.81,6.32) | 0.05(0.04,0.06) | -3.29(-3.52,-3.06) |
| Comoros | 0.70(0.32,1.18) | 0.33(0.15,0.55) | 0.57(0.28,1.06) | 0.24(0.12,0.44) | -1.22(-1.63,-0.81) |
| Congo | 0.42(0.22,0.72) | 0.04(0.02,0.07) | 0.46(0.28,0.78) | 0.02(0.01,0.04) | -1.63(-1.82,-1.44) |
| Cook Islands | 0.00(0.00,0.00) | 0.01(0.00,0.02) | 0.00(0.00,0.00) | 0.01(0.00,0.02) | -1.34(-1.98,-0.69) |
| Costa Rica | 2.25(1.87,2.65) | 0.20(0.17,0.24) | 0.51(0.40,0.65) | 0.05(0.04,0.06) | -4.44(-4.84,-4.03) |
| Croatia | 0.54(0.43,0.67) | 0.05(0.04,0.07) | 0.04(0.03,0.06) | 0.01(0.01,0.01) | -6.17(-6.50,-5.84) |
| Cuba | 2.17(1.50,2.88) | 0.09(0.06,0.12) | 0.52(0.36,0.72) | 0.03(0.02,0.04) | -3.44(-3.71,-3.17) |
| Cyprus | 0.07(0.03,0.12) | 0.04(0.02,0.06) | 0.02(0.01,0.03) | 0.01(0.00,0.01) | -4.85(-5.89,-3.79) |
| Czechia | 2.14(1.61,2.82) | 0.10(0.07,0.13) | 0.19(0.13,0.28) | 0.01(0.01,0.02) | -7.23(-7.61,-6.86) |
| Côte d'Ivoire | 7.20(2.44,12.86) | 0.13(0.04,0.23) | 10.56(3.81,19.48) | 0.09(0.03,0.17) | -0.81(-0.98,-0.63) |
| Democratic People's Republic of Korea | 2.45(1.20,4.36) | 0.04(0.02,0.07) | 0.79(0.32,1.83) | 0.02(0.01,0.04) | -2.89(-3.07,-2.72) |
| Democratic Republic of the Congo | 7.23(3.68,12.43) | 0.04(0.02,0.07) | 10.48(6.38,17.15) | 0.03(0.02,0.05) | -0.89(-1.05,-0.73) |
| Denmark | 0.32(0.27,0.38) | 0.04(0.03,0.04) | 0.04(0.03,0.05) | 0.00(0.00,0.01) | -6.53(-7.19,-5.87) |
| Djibouti | 0.54(0.30,0.90) | 0.31(0.17,0.52) | 0.85(0.40,1.56) | 0.21(0.10,0.38) | -1.22(-1.64,-0.80) |
| Dominica | 0.01(0.00,0.01) | 0.03(0.02,0.04) | 0.01(0.00,0.01) | 0.04(0.02,0.06) | 0.98(0.79,1.18) |
| Dominican Republic | 0.23(0.13,0.52) | 0.01(0.00,0.02) | 0.15(0.06,0.32) | 0.01(0.00,0.01) | -2.37(-2.87,-1.87) |
| Ecuador | 2.59(1.90,3.59) | 0.07(0.05,0.09) | 2.00(1.36,2.83) | 0.04(0.03,0.06) | -1.19(-1.84,-0.54) |
| Egypt | 6.88(2.71,17.80) | 0.03(0.01,0.08) | 3.93(1.32,11.32) | 0.01(0.00,0.03) | -2.75(-3.63,-1.87) |
| El Salvador | 2.33(1.48,3.47) | 0.11(0.07,0.16) | 0.81(0.53,1.30) | 0.04(0.03,0.07) | -2.56(-2.90,-2.23) |
| Equatorial Guinea | 0.10(0.05,0.20) | 0.05(0.02,0.10) | 0.11(0.05,0.26) | 0.02(0.01,0.04) | -3.89(-4.24,-3.54) |
| Eritrea | 6.19(3.55,9.68) | 0.39(0.22,0.61) | 7.30(3.45,12.90) | 0.29(0.14,0.51) | -0.80(-0.97,-0.64) |
| Estonia | 0.40(0.28,0.55) | 0.11(0.08,0.16) | 0.03(0.02,0.04) | 0.01(0.01,0.02) | -6.51(-6.76,-6.26) |
| Eswatini | 0.26(0.15,0.43) | 0.07(0.04,0.11) | 0.25(0.14,0.43) | 0.06(0.03,0.10) | -0.13(-0.27,0.01) |
| Ethiopia | 142.83(73.32,245.14) | 0.59(0.30,1.01) | 127.48(71.58,236.84) | 0.29(0.16,0.53) | -2.52(-2.68,-2.37) |
| Fiji | 0.12(0.05,0.26) | 0.04(0.02,0.09) | 0.17(0.07,0.31) | 0.06(0.03,0.11) | 1.88(1.49,2.27) |
| Finland | 0.31(0.25,0.38) | 0.03(0.03,0.04) | 0.05(0.04,0.07) | 0.01(0.00,0.01) | -5.67(-6.48,-4.85) |
| France | 3.28(2.61,4.23) | 0.03(0.02,0.04) | 0.77(0.51,1.15) | 0.01(0.00,0.01) | -4.93(-5.12,-4.74) |
| Gabon | 0.13(0.07,0.22) | 0.03(0.02,0.05) | 0.13(0.08,0.22) | 0.02(0.01,0.03) | -1.17(-1.30,-1.04) |
| Gambia | 0.52(0.16,1.01) | 0.11(0.03,0.22) | 0.70(0.30,1.36) | 0.07(0.03,0.14) | -1.65(-1.89,-1.40) |
| Georgia | 3.04(1.52,4.41) | 0.22(0.11,0.32) | 0.44(0.30,0.62) | 0.06(0.04,0.08) | -3.51(-4.22,-2.80) |
| Germany | 4.21(3.45,5.10) | 0.03(0.03,0.04) | 0.96(0.70,1.31) | 0.01(0.01,0.01) | -3.84(-4.44,-3.24) |
| Ghana | 0.30(0.09,0.67) | 0.00(0.00,0.01) | 0.14(0.05,0.33) | 0.00(0.00,0.00) | -6.04(-6.88,-5.20) |
| Greece | 1.34(1.17,1.52) | 0.07(0.06,0.07) | 0.27(0.22,0.33) | 0.02(0.02,0.02) | -3.53(-3.87,-3.19) |
| Greenland | 0.01(0.00,0.01) | 0.04(0.01,0.09) | 0.00(0.00,0.00) | 0.01(0.00,0.01) | -5.42(-6.02,-4.83) |
| Grenada | 0.01(0.01,0.01) | 0.03(0.02,0.04) | 0.00(0.00,0.00) | 0.01(0.01,0.02) | -2.10(-2.70,-1.50) |
| Guam | 0.01(0.01,0.02) | 0.02(0.01,0.04) | 0.01(0.00,0.01) | 0.02(0.01,0.03) | -0.85(-2.00,0.31) |
| Guatemala | 4.13(2.36,5.22) | 0.10(0.06,0.13) | 1.39(1.15,1.67) | 0.03(0.02,0.03) | -3.15(-3.45,-2.84) |
| Guinea | 6.09(1.65,11.91) | 0.22(0.06,0.43) | 7.91(2.73,17.17) | 0.13(0.05,0.28) | -1.42(-1.54,-1.29) |
| Guinea-Bissau | 0.23(0.10,0.40) | 0.05(0.02,0.08) | 0.13(0.05,0.30) | 0.01(0.01,0.03) | -4.40(-4.75,-4.05) |
| Guyana | 0.13(0.11,0.15) | 0.04(0.04,0.05) | 0.06(0.04,0.07) | 0.03(0.02,0.03) | -0.39(-1.05,0.28) |
| Haiti | 2.52(1.15,4.53) | 0.09(0.04,0.17) | 2.94(1.46,4.86) | 0.07(0.03,0.11) | -0.84(-0.92,-0.76) |
| Honduras | 1.59(0.78,2.39) | 0.07(0.04,0.11) | 0.71(0.36,1.31) | 0.02(0.01,0.04) | -3.91(-4.08,-3.75) |
| Hungary | 1.31(0.92,1.74) | 0.06(0.04,0.08) | 0.09(0.06,0.13) | 0.01(0.00,0.01) | -6.20(-6.76,-5.63) |
| Iceland | 0.02(0.02,0.03) | 0.04(0.03,0.05) | 0.00(0.00,0.01) | 0.01(0.00,0.01) | -5.79(-7.11,-4.45) |
| India | 625.57(301.21,876.22) | 0.19(0.09,0.27) | 258.81(158.36,408.01) | 0.07(0.04,0.11) | -3.04(-3.24,-2.85) |
| Indonesia | 23.43(15.72,44.53) | 0.03(0.02,0.07) | 9.98(5.06,23.95) | 0.01(0.01,0.04) | -2.62(-2.66,-2.57) |
| Iran (Islamic Republic of) | 15.44(10.91,25.25) | 0.06(0.04,0.10) | 8.74(4.02,12.82) | 0.04(0.02,0.06) | -0.04(-0.77,0.69) |
| Iraq | 13.15(5.32,21.49) | 0.16(0.06,0.26) | 7.72(3.45,14.23) | 0.06(0.03,0.11) | -3.17(-3.52,-2.82) |
| Ireland | 0.34(0.27,0.44) | 0.04(0.03,0.05) | 0.06(0.04,0.08) | 0.01(0.00,0.01) | -6.16(-6.89,-5.42) |
| Israel | 0.47(0.38,0.58) | 0.03(0.02,0.04) | 0.19(0.15,0.25) | 0.01(0.01,0.01) | -4.14(-4.98,-3.30) |
| Italy | 4.70(4.35,5.13) | 0.05(0.05,0.06) | 1.16(1.02,1.33) | 0.02(0.01,0.02) | -3.65(-4.06,-3.24) |
| Jamaica | 0.67(0.52,0.86) | 0.08(0.06,0.10) | 0.06(0.05,0.09) | 0.01(0.01,0.02) | -6.41(-7.53,-5.28) |
| Japan | 0.95(0.91,0.99) | 0.00(0.00,0.00) | 0.32(0.30,0.34) | 0.00(0.00,0.00) | -1.78(-2.20,-1.37) |
| Jordan | 0.70(0.44,1.17) | 0.04(0.03,0.07) | 0.88(0.54,1.33) | 0.02(0.01,0.04) | -1.71(-2.01,-1.42) |
| Kazakhstan | 3.79(3.19,4.55) | 0.07(0.06,0.09) | 1.42(1.15,1.74) | 0.03(0.02,0.03) | -3.57(-4.20,-2.94) |
| Kenya | 11.18(7.06,18.20) | 0.10(0.06,0.16) | 15.08(8.08,30.76) | 0.08(0.04,0.16) | 0.36(-0.07,0.79) |
| Kiribati | 0.00(0.00,0.01) | 0.01(0.00,0.02) | 0.00(0.00,0.01) | 0.01(0.00,0.02) | -0.24(-0.35,-0.14) |
| Kuwait | 0.95(0.75,1.17) | 0.17(0.14,0.21) | 0.13(0.09,0.17) | 0.02(0.01,0.02) | -2.83(-5.15,-0.45) |
| Kyrgyzstan | 2.97(2.10,4.15) | 0.18(0.12,0.25) | 0.68(0.45,0.94) | 0.03(0.02,0.04) | -3.87(-4.77,-2.96) |
| Lao People's Democratic Republic | 0.98(0.49,2.00) | 0.05(0.03,0.11) | 0.46(0.20,1.08) | 0.02(0.01,0.05) | -3.13(-3.22,-3.05) |
| Latvia | 0.62(0.43,0.84) | 0.11(0.08,0.15) | 0.03(0.02,0.05) | 0.01(0.01,0.02) | -6.05(-6.52,-5.57) |
| Lebanon | 1.80(0.88,3.02) | 0.17(0.08,0.29) | 0.78(0.40,1.32) | 0.06(0.03,0.10) | -3.57(-3.85,-3.30) |
| Lesotho | 0.38(0.21,0.65) | 0.06(0.03,0.10) | 0.46(0.23,0.82) | 0.07(0.04,0.13) | 1.41(1.19,1.64) |
| Liberia | 0.40(0.18,0.67) | 0.04(0.02,0.06) | 0.31(0.14,0.64) | 0.01(0.01,0.03) | -4.07(-4.56,-3.58) |
| Libya | 3.96(2.19,6.25) | 0.22(0.12,0.34) | 2.51(1.35,3.95) | 0.17(0.09,0.26) | -0.55(-0.85,-0.25) |
| Lithuania | 0.82(0.62,1.07) | 0.10(0.07,0.13) | 0.08(0.05,0.11) | 0.02(0.01,0.03) | -5.09(-5.56,-4.62) |
| Luxembourg | 0.01(0.01,0.01) | 0.02(0.02,0.02) | 0.00(0.00,0.00) | 0.00(0.00,0.00) | -6.47(-7.01,-5.93) |
| Madagascar | 16.16(9.17,25.88) | 0.30(0.17,0.47) | 23.79(12.38,40.84) | 0.20(0.11,0.35) | -0.92(-1.06,-0.78) |
| Malawi | 12.53(6.11,21.08) | 0.28(0.13,0.46) | 11.20(4.32,21.92) | 0.14(0.05,0.27) | -2.11(-2.27,-1.96) |
| Malaysia | 2.82(0.95,5.17) | 0.04(0.01,0.08) | 1.36(0.56,2.38) | 0.02(0.01,0.03) | -2.86(-3.27,-2.44) |
| Maldives | 0.05(0.03,0.07) | 0.04(0.02,0.07) | 0.01(0.01,0.02) | 0.01(0.01,0.02) | -3.96(-4.29,-3.62) |
| Mali | 11.17(2.76,22.65) | 0.27(0.07,0.55) | 15.85(5.57,33.24) | 0.14(0.05,0.29) | -1.99(-2.12,-1.85) |
| Malta | 0.03(0.02,0.04) | 0.03(0.02,0.04) | 0.02(0.01,0.02) | 0.02(0.02,0.03) | -3.20(-4.46,-1.94) |
| Marshall Islands | 0.00(0.00,0.00) | 0.01(0.00,0.02) | 0.00(0.00,0.00) | 0.01(0.00,0.02) | -0.29(-0.63,0.04) |
| Mauritania | 0.26(0.13,0.43) | 0.03(0.01,0.05) | 0.16(0.07,0.35) | 0.01(0.00,0.02) | -4.48(-4.79,-4.16) |
| Mauritius | 0.10(0.09,0.11) | 0.03(0.03,0.03) | 0.03(0.03,0.04) | 0.02(0.01,0.02) | -3.56(-4.67,-2.44) |
| Mexico | 74.07(68.74,80.44) | 0.22(0.21,0.24) | 14.69(12.90,16.83) | 0.05(0.04,0.05) | -4.43(-4.78,-4.08) |
| Micronesia (Federated States of) | 0.01(0.00,0.01) | 0.01(0.00,0.03) | 0.00(0.00,0.00) | 0.01(0.00,0.02) | -1.89(-1.95,-1.83) |
| Monaco | 0.00(0.00,0.00) | 0.05(0.03,0.10) | 0.00(0.00,0.00) | 0.03(0.01,0.08) | -2.68(-3.18,-2.17) |
| Mongolia | 0.80(0.36,1.63) | 0.09(0.04,0.18) | 0.58(0.34,0.93) | 0.05(0.03,0.09) | -1.81(-2.47,-1.15) |
| Montenegro | 0.15(0.09,0.21) | 0.09(0.06,0.13) | 0.03(0.02,0.06) | 0.03(0.02,0.05) | -3.68(-4.34,-3.01) |
| Morocco | 17.93(8.67,30.51) | 0.18(0.09,0.31) | 6.71(3.28,12.09) | 0.07(0.03,0.12) | -2.65(-2.95,-2.35) |
| Mozambique | 30.52(16.80,46.42) | 0.49(0.27,0.75) | 41.13(16.01,75.59) | 0.29(0.11,0.53) | -1.17(-1.46,-0.88) |
| Myanmar | 8.16(3.88,16.63) | 0.06(0.03,0.11) | 3.05(1.30,7.34) | 0.02(0.01,0.05) | -3.71(-3.88,-3.53) |
| Namibia | 0.54(0.24,0.88) | 0.09(0.04,0.15) | 0.67(0.31,1.17) | 0.08(0.04,0.14) | -0.02(-0.17,0.14) |
| Nauru | 0.00(0.00,0.00) | 0.01(0.00,0.02) | 0.00(0.00,0.00) | 0.01(0.00,0.02) | -0.37(-0.78,0.05) |
| Nepal | 22.59(11.27,42.53) | 0.27(0.13,0.50) | 10.24(4.63,24.00) | 0.11(0.05,0.26) | -2.52(-2.72,-2.33) |
| Netherlands | 1.36(1.10,1.65) | 0.05(0.04,0.06) | 0.40(0.31,0.52) | 0.01(0.01,0.02) | -3.48(-3.75,-3.20) |
| New Zealand | 0.09(0.08,0.12) | 0.01(0.01,0.01) | 0.04(0.03,0.06) | 0.00(0.00,0.01) | -3.71(-4.95,-2.46) |
| Nicaragua | 1.58(0.95,2.30) | 0.09(0.05,0.13) | 0.48(0.30,0.85) | 0.02(0.02,0.04) | -3.41(-3.69,-3.13) |
| Niger | 1.91(0.86,3.28) | 0.05(0.02,0.08) | 1.78(0.74,3.97) | 0.01(0.01,0.03) | -4.57(-5.00,-4.14) |
| Nigeria | 256.12(43.98,446.66) | 0.65(0.11,1.14) | 433.88(79.75,814.52) | 0.43(0.08,0.80) | -1.49(-1.63,-1.36) |
| Niue | 0.00(0.00,0.00) | 0.01(0.00,0.02) | 0.00(0.00,0.00) | 0.02(0.01,0.04) | -0.02(-1.07,1.04) |
| North Macedonia | 0.73(0.44,1.02) | 0.14(0.08,0.19) | 0.14(0.10,0.27) | 0.04(0.03,0.08) | -3.80(-4.35,-3.25) |
| Northern Mariana Islands | 0.00(0.00,0.00) | 0.00(0.00,0.00) | 0.00(0.00,0.00) | 0.00(0.00,0.00) | 2.35(1.85,2.84) |
| Norway | 0.04(0.04,0.04) | 0.00(0.00,0.01) | 0.00(0.00,0.00) | 0.00(0.00,0.00) | -8.06(-9.65,-6.45) |
| Oman | 0.89(0.37,1.56) | 0.11(0.04,0.19) | 0.38(0.16,0.66) | 0.03(0.01,0.05) | -3.16(-3.46,-2.85) |
| Pakistan | 232.77(118.65,350.69) | 0.47(0.24,0.71) | 379.65(209.15,587.72) | 0.44(0.24,0.69) | -0.05(-0.24,0.14) |
| Palau | 0.00(0.00,0.00) | 0.00(0.00,0.00) | 0.00(0.00,0.00) | 0.00(0.00,0.00) | -1.53(-1.65,-1.41) |
| Palestine | 1.34(0.81,1.99) | 0.14(0.08,0.21) | 1.29(0.75,1.95) | 0.07(0.04,0.10) | -1.55(-1.95,-1.14) |
| Panama | 0.60(0.46,0.70) | 0.07(0.06,0.08) | 0.36(0.28,0.44) | 0.03(0.02,0.04) | -2.32(-2.56,-2.07) |
| Papua New Guinea | 0.20(0.06,0.42) | 0.01(0.00,0.02) | 0.37(0.12,0.73) | 0.01(0.00,0.02) | -0.58(-0.74,-0.42) |
| Paraguay | 1.03(0.58,1.63) | 0.06(0.03,0.10) | 0.72(0.42,1.23) | 0.04(0.02,0.06) | -1.53(-1.86,-1.19) |
| Peru | 5.90(3.78,9.93) | 0.07(0.05,0.12) | 2.45(1.48,4.34) | 0.03(0.02,0.05) | -3.07(-3.22,-2.91) |
| Philippines | 4.03(2.49,6.04) | 0.02(0.01,0.02) | 3.45(2.42,5.13) | 0.01(0.01,0.02) | -0.88(-1.05,-0.72) |
| Poland | 8.86(8.33,9.37) | 0.09(0.09,0.10) | 0.60(0.54,0.67) | 0.01(0.01,0.01) | -7.39(-7.84,-6.93) |
| Portugal | 1.10(0.87,1.39) | 0.05(0.04,0.07) | 0.13(0.09,0.18) | 0.01(0.01,0.01) | -6.00(-6.31,-5.70) |
| Puerto Rico | 0.42(0.33,0.55) | 0.04(0.03,0.05) | 0.03(0.02,0.04) | 0.01(0.01,0.01) | -5.44(-5.83,-5.05) |
| Qatar | 0.03(0.02,0.07) | 0.03(0.01,0.05) | 0.05(0.03,0.11) | 0.01(0.01,0.02) | -2.27(-2.73,-1.81) |
| Republic of Korea | 1.02(0.64,1.73) | 0.01(0.01,0.02) | 0.11(0.07,0.20) | 0.00(0.00,0.00) | -4.81(-5.09,-4.53) |
| Republic of Moldova | 2.89(2.45,3.39) | 0.23(0.20,0.27) | 0.24(0.19,0.30) | 0.05(0.04,0.06) | -4.92(-5.26,-4.57) |
| Romania | 7.53(5.72,9.65) | 0.14(0.10,0.17) | 0.51(0.33,0.72) | 0.02(0.01,0.02) | -7.36(-7.78,-6.95) |
| Russian Federation | 47.13(44.75,49.90) | 0.14(0.13,0.14) | 6.12(5.65,6.62) | 0.02(0.02,0.03) | -5.80(-6.02,-5.57) |
| Rwanda | 16.83(9.83,25.77) | 0.50(0.29,0.76) | 10.38(4.65,19.44) | 0.21(0.09,0.39) | -3.14(-3.35,-2.92) |
| Saint Kitts and Nevis | 0.00(0.00,0.00) | 0.00(0.00,0.00) | 0.00(0.00,0.00) | 0.00(0.00,0.01) | 4.21(1.88,6.61) |
| Saint Lucia | 0.03(0.02,0.03) | 0.05(0.04,0.07) | 0.01(0.01,0.01) | 0.03(0.02,0.04) | -2.19(-2.62,-1.76) |
| Saint Vincent and the Grenadines | 0.02(0.02,0.03) | 0.06(0.05,0.07) | 0.01(0.01,0.01) | 0.03(0.02,0.03) | -2.29(-2.89,-1.67) |
| Samoa | 0.08(0.04,0.14) | 0.11(0.05,0.20) | 0.05(0.02,0.11) | 0.07(0.03,0.13) | -1.55(-1.68,-1.41) |
| San Marino | 0.00(0.00,0.00) | 0.06(0.03,0.10) | 0.00(0.00,0.00) | 0.02(0.01,0.04) | -3.17(-3.50,-2.83) |
| Sao Tome and Principe | 0.00(0.00,0.00) | 0.00(0.00,0.00) | 0.00(0.00,0.00) | 0.00(0.00,0.00) | -3.04(-3.31,-2.77) |
| Saudi Arabia | 4.61(1.00,9.93) | 0.07(0.02,0.15) | 1.42(0.25,3.15) | 0.02(0.00,0.04) | -4.18(-4.55,-3.80) |
| Senegal | 1.38(0.64,2.31) | 0.04(0.02,0.06) | 0.73(0.32,1.67) | 0.01(0.01,0.03) | -4.29(-4.56,-4.01) |
| Serbia | 2.24(1.22,3.67) | 0.10(0.06,0.17) | 0.28(0.14,0.59) | 0.02(0.01,0.04) | -5.70(-6.13,-5.27) |
| Seychelles | 0.01(0.00,0.01) | 0.03(0.02,0.05) | 0.00(0.00,0.00) | 0.01(0.00,0.01) | -2.59(-3.41,-1.77) |
| Sierra Leone | 0.63(0.26,1.09) | 0.03(0.01,0.06) | 0.51(0.23,1.02) | 0.01(0.01,0.03) | -3.25(-3.50,-3.00) |
| Singapore | 0.09(0.07,0.12) | 0.01(0.01,0.02) | 0.02(0.02,0.03) | 0.00(0.00,0.00) | -2.91(-4.10,-1.70) |
| Slovakia | 0.92(0.54,1.38) | 0.07(0.04,0.10) | 0.18(0.10,0.30) | 0.02(0.01,0.03) | -4.01(-4.23,-3.80) |
| Slovenia | 0.21(0.17,0.26) | 0.05(0.04,0.06) | 0.02(0.01,0.02) | 0.01(0.00,0.01) | -6.63(-7.14,-6.12) |
| Solomon Islands | 0.02(0.01,0.04) | 0.01(0.00,0.02) | 0.02(0.01,0.05) | 0.01(0.00,0.02) | -0.55(-0.70,-0.39) |
| Somalia | 12.93(6.91,22.08) | 0.33(0.18,0.57) | 26.46(11.58,49.14) | 0.26(0.11,0.48) | -0.58(-0.93,-0.23) |
| South Africa | 5.20(3.62,8.66) | 0.04(0.03,0.06) | 5.64(3.61,8.24) | 0.04(0.02,0.05) | -0.70(-1.07,-0.32) |
| South Sudan | 7.85(4.28,13.29) | 0.30(0.16,0.51) | 12.74(7.34,20.06) | 0.30(0.17,0.47) | 0.14(-0.37,0.64) |
| Spain | 3.92(3.15,4.95) | 0.05(0.04,0.06) | 0.67(0.50,0.91) | 0.01(0.01,0.01) | -5.27(-5.55,-4.98) |
| Sri Lanka | 2.76(1.68,4.15) | 0.05(0.03,0.08) | 0.67(0.37,1.27) | 0.01(0.01,0.02) | -5.02(-5.60,-4.44) |
| Sudan | 12.26(6.20,24.09) | 0.14(0.07,0.27) | 14.03(7.56,24.16) | 0.08(0.05,0.15) | -1.21(-1.41,-1.00) |
| Suriname | 0.05(0.03,0.08) | 0.04(0.02,0.06) | 0.04(0.03,0.07) | 0.03(0.02,0.05) | -0.73(-1.03,-0.42) |
| Sweden | 0.12(0.10,0.15) | 0.01(0.01,0.01) | 0.06(0.04,0.08) | 0.00(0.00,0.00) | -1.97(-3.06,-0.86) |
| Switzerland | 0.36(0.29,0.43) | 0.03(0.03,0.04) | 0.05(0.04,0.07) | 0.00(0.00,0.01) | -6.85(-7.64,-6.05) |
| Syrian Arab Republic | 0.31(0.20,0.44) | 0.01(0.00,0.01) | 0.11(0.06,0.16) | 0.00(0.00,0.00) | -1.29(-1.88,-0.70) |
| Taiwan (Province of China) | 0.75(0.61,0.89) | 0.01(0.01,0.02) | 0.07(0.06,0.09) | 0.00(0.00,0.00) | -5.52(-5.86,-5.17) |
| Tajikistan | 0.68(0.33,1.17) | 0.03(0.01,0.05) | 0.75(0.42,1.41) | 0.02(0.01,0.04) | -1.64(-1.99,-1.29) |
| Thailand | 5.57(3.12,8.47) | 0.03(0.02,0.05) | 0.83(0.41,1.75) | 0.01(0.00,0.02) | -4.89(-5.26,-4.53) |
| Timor-Leste | 0.13(0.07,0.25) | 0.04(0.02,0.07) | 0.09(0.04,0.22) | 0.02(0.01,0.04) | -2.53(-2.86,-2.19) |
| Togo | 0.57(0.27,0.94) | 0.03(0.02,0.05) | 0.36(0.16,0.73) | 0.01(0.00,0.02) | -4.12(-4.42,-3.82) |
| Tokelau | 0.00(0.00,0.00) | 0.01(0.00,0.02) | 0.00(0.00,0.00) | 0.03(0.01,0.07) | -0.30(-1.82,1.24) |
| Tonga | 0.00(0.00,0.00) | 0.00(0.00,0.01) | 0.00(0.00,0.00) | 0.00(0.00,0.01) | -0.78(-0.96,-0.61) |
| Trinidad and Tobago | 0.15(0.11,0.18) | 0.04(0.03,0.04) | 0.04(0.03,0.05) | 0.01(0.01,0.02) | -3.45(-3.84,-3.05) |
| Tunisia | 8.37(4.28,13.01) | 0.27(0.14,0.42) | 2.56(1.28,4.35) | 0.09(0.05,0.16) | -3.43(-3.51,-3.36) |
| Turkey | 22.71(13.60,41.79) | 0.11(0.07,0.20) | 5.62(3.24,11.18) | 0.03(0.02,0.06) | -4.11(-4.47,-3.75) |
| Turkmenistan | 1.80(1.44,2.22) | 0.12(0.10,0.15) | 1.79(1.42,2.24) | 0.12(0.09,0.15) | -0.20(-0.74,0.34) |
| Tuvalu | 0.00(0.00,0.00) | 0.01(0.00,0.03) | 0.00(0.00,0.00) | 0.01(0.00,0.02) | -2.00(-2.10,-1.90) |
| Uganda | 35.17(18.91,53.89) | 0.42(0.22,0.64) | 72.54(31.42,125.94) | 0.37(0.16,0.63) | -0.50(-0.70,-0.30) |
| Ukraine | 7.63(4.59,10.36) | 0.07(0.04,0.09) | 2.30(1.72,3.10) | 0.04(0.03,0.05) | -2.86(-3.33,-2.38) |
| United Arab Emirates | 0.33(0.17,0.61) | 0.06(0.03,0.10) | 0.23(0.13,0.47) | 0.02(0.01,0.03) | -3.52(-3.88,-3.15) |
| United Kingdom | 2.64(2.53,2.76) | 0.02(0.02,0.03) | 0.91(0.85,0.96) | 0.01(0.01,0.01) | -3.26(-3.54,-2.98) |
| United Republic of Tanzania | 43.86(25.57,65.84) | 0.36(0.21,0.55) | 54.78(27.42,96.74) | 0.22(0.11,0.40) | -1.01(-1.26,-0.76) |
| United States Virgin Islands | 0.00(0.00,0.01) | 0.01(0.01,0.02) | 0.00(0.00,0.00) | 0.00(0.00,0.01) | -2.43(-2.84,-2.01) |
| United States of America | 11.26(10.97,11.55) | 0.02(0.02,0.02) | 3.18(2.99,3.38) | 0.01(0.01,0.01) | -4.41(-4.56,-4.26) |
| Uruguay | 0.46(0.36,0.57) | 0.06(0.04,0.07) | 0.14(0.11,0.18) | 0.02(0.02,0.03) | -3.35(-3.99,-2.72) |
| Uzbekistan | 11.01(7.51,15.33) | 0.13(0.09,0.18) | 7.22(5.03,10.15) | 0.07(0.05,0.10) | -1.83(-2.22,-1.44) |
| Vanuatu | 0.01(0.00,0.01) | 0.01(0.00,0.02) | 0.01(0.00,0.02) | 0.01(0.00,0.02) | -0.13(-0.34,0.08) |
| Venezuela (Bolivarian Republic of) | 6.42(5.41,7.41) | 0.09(0.08,0.10) | 4.13(3.12,5.26) | 0.06(0.05,0.08) | -1.30(-1.67,-0.91) |
| Viet Nam | 14.07(5.35,24.84) | 0.05(0.02,0.09) | 7.13(3.06,12.74) | 0.03(0.01,0.05) | -1.99(-2.13,-1.85) |
| Yemen | 7.08(4.01,12.71) | 0.10(0.06,0.18) | 10.78(5.89,19.37) | 0.08(0.04,0.14) | -0.93(-1.03,-0.83) |
| Zambia | 12.78(7.25,20.38) | 0.34(0.19,0.54) | 16.28(7.57,30.32) | 0.20(0.09,0.37) | -1.68(-1.96,-1.39) |
| Zimbabwe | 2.92(1.15,4.74) | 0.06(0.02,0.10) | 6.36(3.09,10.25) | 0.10(0.05,0.16) | 3.11(2.21,4.02) |
| ^a^EAPC is expressed as 95% CIs | | | | | |

**Table S4** Death of Non-Hodgkin lymphoma in Children Between 1990 and 2021 at 204 Countries and Territories.

| Location | Rate per 100000 (95% UI) | | | | |
| --- | --- | --- | --- | --- | --- |
|  | 1990 | | 2021 | | EAPC^a^ |
|  | Cases | Rates | Cases | Rates |  |
| Global | 12990.00(9904.43,15509.02) | 0.75(0.57,0.89) | 9036.49(7197.45,11251.98) | 0.45(0.36,0.56) | -1.49(-1.60,-1.39) |
| 5 SDI | | | | | |
| High SDI | 505.04(481.15,530.35) | 0.27(0.26,0.29) | 182.62(169.57,196.35) | 0.11(0.10,0.11) | -3.12(-3.21,-3.03) |
| High-middle SDI | 1633.11(1422.84,1867.51) | 0.60(0.52,0.68) | 439.50(380.19,519.97) | 0.19(0.16,0.23) | -3.69(-3.88,-3.51) |
| Middle SDI | 3591.85(3029.39,4179.98) | 0.62(0.52,0.72) | 1499.26(1233.04,1855.05) | 0.26(0.22,0.33) | -2.59(-2.76,-2.41) |
| Low-middle SDI | 3626.63(2446.64,4617.89) | 0.77(0.52,0.98) | 2750.39(2217.99,3493.93) | 0.47(0.38,0.60) | -1.41(-1.50,-1.32) |
| Low SDI | 3622.77(2370.30,4792.28) | 1.58(1.04,2.09) | 4157.48(2868.69,5570.47) | 0.90(0.62,1.21) | -1.68(-1.78,-1.57) |
| 21 GBD Region | | | | | |
| Andean Latin America | 119.37(99.87,148.00) | 0.80(0.67,1.00) | 70.20(56.06,90.20) | 0.39(0.31,0.50) | -2.20(-2.38,-2.01) |
| Australasia | 11.26(10.06,12.64) | 0.25(0.22,0.28) | 4.54(3.78,5.33) | 0.08(0.07,0.09) | -3.57(-4.12,-3.02) |
| Caribbean | 141.92(84.98,192.00) | 1.24(0.74,1.68) | 91.68(55.17,131.85) | 0.80(0.48,1.15) | -1.06(-1.33,-0.79) |
| Central Asia | 159.40(130.76,185.09) | 0.64(0.52,0.74) | 97.61(80.56,120.99) | 0.35(0.29,0.44) | -2.02(-2.21,-1.84) |
| Central Europe | 126.64(119.22,134.40) | 0.43(0.40,0.46) | 30.14(27.25,33.40) | 0.17(0.15,0.19) | -2.87(-3.15,-2.60) |
| Central Latin America | 336.69(315.01,360.91) | 0.52(0.49,0.56) | 148.67(127.00,175.72) | 0.23(0.20,0.28) | -2.08(-2.28,-1.89) |
| Central Sub-Saharan Africa | 204.86(63.28,302.80) | 0.81(0.25,1.20) | 198.83(134.58,274.79) | 0.34(0.23,0.47) | -2.45(-2.65,-2.25) |
| East Asia | 2454.63(2046.08,2989.85) | 0.74(0.62,0.91) | 503.68(406.26,645.39) | 0.19(0.15,0.24) | -4.47(-4.82,-4.12) |
| Eastern Europe | 303.94(288.01,316.97) | 0.59(0.56,0.62) | 67.14(62.54,72.41) | 0.19(0.18,0.20) | -3.79(-4.20,-3.37) |
| Eastern Sub-Saharan Africa | 1971.94(1397.50,2650.84) | 2.18(1.54,2.93) | 2094.62(1418.15,2964.12) | 1.17(0.79,1.66) | -1.80(-1.93,-1.67) |
| High-income Asia Pacific | 119.96(109.41,135.53) | 0.34(0.31,0.39) | 29.51(27.51,31.79) | 0.13(0.12,0.14) | -3.26(-3.49,-3.02) |
| High-income North America | 120.93(118.02,124.24) | 0.20(0.19,0.20) | 47.27(43.95,50.38) | 0.07(0.07,0.08) | -3.39(-3.57,-3.21) |
| North Africa and Middle East | 830.13(618.27,1104.17) | 0.59(0.44,0.79) | 469.17(389.87,610.37) | 0.26(0.21,0.33) | -2.44(-2.64,-2.24) |
| Oceania | 6.86(4.41,9.96) | 0.26(0.16,0.37) | 15.32(10.16,22.75) | 0.30(0.20,0.45) | 0.72(0.49,0.95) |
| South Asia | 3177.06(1996.32,4138.23) | 0.73(0.46,0.95) | 2139.59(1680.09,2795.58) | 0.42(0.33,0.55) | -1.71(-1.83,-1.58) |
| Southeast Asia | 879.41(560.12,1168.28) | 0.52(0.33,0.68) | 472.77(383.69,638.70) | 0.27(0.22,0.37) | -1.93(-1.99,-1.87) |
| Southern Latin America | 60.70(55.33,66.99) | 0.41(0.37,0.45) | 29.19(24.82,34.24) | 0.20(0.17,0.24) | -1.95(-2.25,-1.64) |
| Southern Sub-Saharan Africa | 75.08(59.79,93.97) | 0.36(0.29,0.45) | 129.99(92.28,166.96) | 0.54(0.38,0.69) | 1.90(1.44,2.36) |
| Tropical Latin America | 296.27(264.35,328.36) | 0.55(0.49,0.61) | 102.23(82.96,120.27) | 0.20(0.17,0.24) | -2.67(-3.12,-2.21) |
| Western Europe | 183.77(177.22,190.86) | 0.26(0.25,0.27) | 76.83(70.61,83.16) | 0.11(0.10,0.12) | -2.83(-2.98,-2.67) |
| Western Sub-Saharan Africa | 1409.17(993.63,1865.93) | 1.60(1.13,2.12) | 2217.51(1423.04,2946.83) | 1.03(0.66,1.37) | -1.24(-1.37,-1.11) |
| 204 countries and territories | | | | | |
| Afghanistan | 29.34(10.60,48.88) | 0.68(0.25,1.13) | 50.89(32.22,83.55) | 0.36(0.23,0.59) | -1.71(-1.99,-1.44) |
| Albania | 4.23(3.08,5.52) | 0.38(0.28,0.49) | 0.80(0.53,1.13) | 0.18(0.12,0.26) | -2.05(-2.81,-1.29) |
| Algeria | 80.81(58.42,104.42) | 0.75(0.54,0.97) | 43.43(31.43,58.75) | 0.33(0.24,0.44) | -2.51(-2.64,-2.37) |
| American Samoa | 0.03(0.02,0.04) | 0.14(0.10,0.21) | 0.03(0.02,0.05) | 0.24(0.15,0.38) | 2.22(1.95,2.49) |
| Andorra | 0.06(0.04,0.09) | 0.64(0.41,0.90) | 0.02(0.01,0.02) | 0.16(0.12,0.23) | -4.05(-4.29,-3.80) |
| Angola | 54.67(12.01,90.56) | 1.16(0.25,1.92) | 59.57(36.46,88.40) | 0.39(0.24,0.58) | -3.31(-3.62,-3.00) |
| Antigua and Barbuda | 0.07(0.06,0.08) | 0.39(0.32,0.45) | 0.05(0.05,0.06) | 0.32(0.28,0.36) | -0.60(-1.13,-0.07) |
| Argentina | 43.52(38.20,49.52) | 0.43(0.38,0.49) | 22.79(18.75,27.40) | 0.22(0.18,0.27) | -1.73(-2.04,-1.42) |
| Armenia | 3.11(2.23,3.80) | 0.30(0.21,0.36) | 1.36(1.08,1.73) | 0.23(0.18,0.29) | 0.69(0.09,1.30) |
| Australia | 9.88(8.74,11.24) | 0.26(0.23,0.30) | 3.70(2.99,4.42) | 0.08(0.06,0.09) | -3.70(-4.19,-3.20) |
| Austria | 2.81(2.58,3.05) | 0.21(0.19,0.23) | 1.24(1.09,1.40) | 0.10(0.08,0.11) | -2.12(-2.39,-1.84) |
| Azerbaijan | 11.40(7.78,15.89) | 0.47(0.32,0.65) | 7.90(5.09,12.26) | 0.33(0.22,0.52) | -1.63(-1.98,-1.27) |
| Bahamas | 0.43(0.37,0.51) | 0.54(0.46,0.63) | 0.27(0.22,0.34) | 0.34(0.27,0.42) | -1.79(-2.21,-1.37) |
| Bahrain | 0.49(0.37,0.63) | 0.30(0.23,0.39) | 0.43(0.31,0.59) | 0.15(0.11,0.20) | -2.41(-2.80,-2.03) |
| Bangladesh | 555.86(242.73,824.00) | 1.14(0.50,1.68) | 210.34(138.55,351.22) | 0.46(0.30,0.77) | -2.70(-2.84,-2.55) |
| Barbados | 0.58(0.50,0.66) | 0.92(0.81,1.06) | 0.21(0.16,0.28) | 0.45(0.33,0.60) | -1.12(-1.63,-0.61) |
| Belarus | 4.03(3.38,4.89) | 0.17(0.14,0.20) | 3.61(2.92,4.56) | 0.23(0.18,0.29) | 2.19(1.52,2.87) |
| Belgium | 4.03(3.51,4.64) | 0.22(0.19,0.26) | 2.74(2.24,3.40) | 0.14(0.12,0.18) | -2.17(-2.43,-1.91) |
| Belize | 0.43(0.38,0.48) | 0.53(0.47,0.59) | 0.23(0.19,0.27) | 0.18(0.16,0.22) | -2.69(-3.00,-2.38) |
| Benin | 32.14(21.06,46.68) | 1.33(0.87,1.93) | 59.24(37.44,84.64) | 0.97(0.62,1.39) | -0.76(-0.91,-0.62) |
| Bermuda | 0.07(0.06,0.08) | 0.58(0.48,0.70) | 0.03(0.02,0.03) | 0.30(0.24,0.37) | -1.72(-2.09,-1.34) |
| Bhutan | 2.31(1.04,3.68) | 0.88(0.40,1.40) | 0.82(0.47,1.55) | 0.44(0.25,0.83) | -2.68(-2.99,-2.37) |
| Bolivia (Plurinational State of) | 33.77(23.90,45.46) | 1.26(0.89,1.69) | 18.99(13.89,25.59) | 0.54(0.40,0.73) | -2.79(-2.90,-2.68) |
| Bosnia and Herzegovina | 2.83(2.19,3.56) | 0.26(0.20,0.32) | 0.81(0.55,1.10) | 0.17(0.11,0.22) | -1.13(-1.44,-0.82) |
| Botswana | 1.77(1.18,2.65) | 0.30(0.20,0.45) | 3.04(1.97,4.52) | 0.44(0.28,0.65) | 1.94(1.55,2.32) |
| Brazil | 288.19(256.27,319.50) | 0.55(0.49,0.62) | 95.73(77.79,112.72) | 0.20(0.16,0.23) | -2.75(-3.22,-2.27) |
| Brunei Darussalam | 0.54(0.39,0.72) | 0.60(0.44,0.80) | 0.26(0.19,0.33) | 0.27(0.20,0.35) | -1.38(-1.87,-0.88) |
| Bulgaria | 7.04(6.23,7.73) | 0.41(0.36,0.45) | 2.15(1.86,2.49) | 0.22(0.19,0.26) | -1.96(-2.43,-1.49) |
| Burkina Faso | 61.87(40.09,88.88) | 1.31(0.85,1.88) | 105.36(72.71,145.54) | 1.02(0.70,1.40) | -0.46(-0.66,-0.27) |
| Burundi | 67.86(42.04,104.56) | 2.59(1.60,3.99) | 54.85(29.97,100.40) | 0.94(0.51,1.71) | -2.76(-2.99,-2.53) |
| Cabo Verde | 0.31(0.18,0.52) | 0.20(0.11,0.33) | 0.99(0.42,1.41) | 0.69(0.29,0.98) | 2.75(1.78,3.74) |
| Cambodia | 28.76(12.35,42.47) | 0.62(0.26,0.91) | 15.67(10.97,25.08) | 0.31(0.21,0.49) | -2.61(-2.85,-2.37) |
| Cameroon | 61.31(40.76,84.70) | 1.26(0.83,1.73) | 143.66(95.96,198.47) | 1.07(0.71,1.47) | -0.14(-0.35,0.07) |
| Canada | 15.07(13.25,17.10) | 0.26(0.23,0.30) | 4.38(3.61,5.29) | 0.07(0.06,0.09) | -5.01(-5.45,-4.57) |
| Central African Republic | 10.68(3.09,17.69) | 0.87(0.25,1.45) | 13.52(6.85,21.15) | 0.59(0.30,0.93) | -1.00(-1.15,-0.85) |
| Chad | 31.27(19.10,47.09) | 1.07(0.65,1.61) | 90.17(56.81,130.48) | 1.00(0.63,1.45) | 0.15(0.01,0.28) |
| Chile | 13.76(12.21,15.52) | 0.35(0.31,0.39) | 4.85(4.09,5.80) | 0.13(0.11,0.16) | -2.93(-3.25,-2.61) |
| China | 2407.27(2002.99,2938.59) | 0.76(0.63,0.92) | 488.34(392.42,627.62) | 0.19(0.15,0.24) | -4.53(-4.89,-4.18) |
| Colombia | 78.41(67.78,89.64) | 0.67(0.58,0.77) | 29.99(23.79,37.72) | 0.28(0.22,0.36) | -1.87(-2.25,-1.48) |
| Comoros | 3.43(1.83,4.96) | 1.61(0.86,2.33) | 2.74(1.79,4.10) | 1.14(0.75,1.71) | -1.22(-1.59,-0.85) |
| Congo | 6.76(2.94,10.33) | 0.64(0.28,0.98) | 6.60(4.67,8.99) | 0.34(0.24,0.47) | -1.97(-2.18,-1.75) |
| Cook Islands | 0.01(0.01,0.01) | 0.12(0.08,0.17) | 0.00(0.00,0.01) | 0.12(0.07,0.23) | -1.46(-2.04,-0.87) |
| Costa Rica | 5.13(4.51,5.96) | 0.46(0.40,0.53) | 3.23(2.60,4.08) | 0.32(0.26,0.40) | -1.20(-1.38,-1.02) |
| Croatia | 3.49(2.88,4.17) | 0.35(0.29,0.42) | 1.15(0.88,1.48) | 0.19(0.15,0.25) | -1.41(-1.98,-0.83) |
| Cuba | 17.41(15.54,19.53) | 0.70(0.62,0.78) | 5.48(4.37,6.70) | 0.31(0.25,0.38) | -2.07(-2.42,-1.72) |
| Cyprus | 0.51(0.38,0.65) | 0.26(0.19,0.33) | 0.19(0.14,0.26) | 0.09(0.06,0.12) | -2.91(-3.83,-1.98) |
| Czechia | 7.44(6.35,8.71) | 0.34(0.29,0.40) | 3.03(2.32,3.81) | 0.18(0.13,0.22) | -1.67(-2.01,-1.34) |
| Côte d'Ivoire | 75.48(53.49,103.92) | 1.32(0.94,1.82) | 130.80(87.43,214.47) | 1.13(0.76,1.85) | -0.17(-0.40,0.05) |
| Democratic People's Republic of Korea | 25.12(15.97,36.56) | 0.42(0.27,0.61) | 9.94(6.54,15.67) | 0.21(0.14,0.33) | -1.99(-2.25,-1.73) |
| Democratic Republic of the Congo | 129.18(43.83,189.18) | 0.73(0.25,1.07) | 114.56(73.17,164.84) | 0.30(0.19,0.43) | -2.38(-2.60,-2.16) |
| Denmark | 1.97(1.71,2.27) | 0.22(0.19,0.26) | 0.84(0.69,1.02) | 0.09(0.07,0.11) | -3.38(-3.65,-3.12) |
| Djibouti | 2.55(1.68,3.67) | 1.46(0.96,2.11) | 3.84(2.30,6.37) | 0.93(0.56,1.54) | -1.37(-1.76,-0.98) |
| Dominica | 0.19(0.15,0.24) | 0.77(0.60,0.98) | 0.17(0.12,0.23) | 1.22(0.89,1.66) | 1.81(1.56,2.06) |
| Dominican Republic | 19.44(14.24,24.51) | 0.72(0.53,0.91) | 9.70(6.49,14.00) | 0.33(0.22,0.48) | -2.64(-2.95,-2.33) |
| Ecuador | 18.16(16.13,20.74) | 0.47(0.42,0.54) | 14.85(11.90,18.46) | 0.29(0.23,0.36) | -1.41(-1.98,-0.83) |
| Egypt | 96.36(72.10,162.04) | 0.43(0.33,0.73) | 42.82(26.69,95.40) | 0.12(0.07,0.26) | -3.99(-4.58,-3.39) |
| El Salvador | 8.27(6.52,10.31) | 0.38(0.30,0.48) | 3.57(2.74,4.62) | 0.20(0.15,0.25) | -1.90(-2.15,-1.66) |
| Equatorial Guinea | 1.52(0.52,2.38) | 0.77(0.27,1.21) | 2.07(1.02,3.98) | 0.35(0.18,0.68) | -3.21(-3.55,-2.86) |
| Eritrea | 28.65(18.82,39.49) | 1.80(1.18,2.48) | 28.84(17.36,49.37) | 1.14(0.69,1.96) | -1.40(-1.56,-1.23) |
| Estonia | 1.03(0.86,1.24) | 0.29(0.25,0.35) | 0.55(0.44,0.67) | 0.25(0.20,0.31) | -0.93(-1.71,-0.14) |
| Eswatini | 1.84(1.07,2.83) | 0.48(0.28,0.73) | 2.16(1.37,3.36) | 0.52(0.33,0.81) | 0.65(0.39,0.91) |
| Ethiopia | 620.68(166.21,965.12) | 2.55(0.68,3.96) | 447.56(311.31,618.16) | 1.01(0.70,1.39) | -3.31(-3.59,-3.04) |
| Fiji | 0.42(0.28,0.64) | 0.15(0.10,0.23) | 0.72(0.47,1.09) | 0.27(0.17,0.40) | 2.19(1.94,2.44) |
| Finland | 2.31(1.94,2.76) | 0.24(0.20,0.29) | 1.22(0.97,1.53) | 0.14(0.11,0.18) | -2.41(-2.82,-1.99) |
| France | 28.91(25.86,31.69) | 0.25(0.22,0.27) | 14.63(12.35,17.10) | 0.13(0.11,0.15) | -2.59(-2.81,-2.37) |
| Gabon | 2.04(1.25,2.92) | 0.50(0.31,0.72) | 2.52(1.62,3.73) | 0.39(0.25,0.58) | -0.21(-0.43,0.01) |
| Gambia | 6.58(4.41,9.09) | 1.43(0.96,1.97) | 8.83(5.75,14.67) | 0.89(0.58,1.48) | -1.69(-1.98,-1.40) |
| Georgia | 9.58(6.85,12.57) | 0.70(0.50,0.92) | 1.33(0.94,1.77) | 0.18(0.13,0.24) | -4.11(-4.82,-3.39) |
| Germany | 28.24(25.44,31.81) | 0.22(0.20,0.25) | 12.63(10.85,14.56) | 0.11(0.09,0.12) | -2.15(-2.39,-1.91) |
| Ghana | 126.85(59.36,193.56) | 1.89(0.88,2.88) | 62.11(40.45,103.52) | 0.48(0.31,0.80) | -5.70(-6.63,-4.76) |
| Greece | 2.13(1.94,2.34) | 0.11(0.10,0.12) | 0.86(0.74,0.98) | 0.06(0.05,0.07) | -1.42(-1.76,-1.08) |
| Greenland | 0.09(0.04,0.12) | 0.60(0.28,0.85) | 0.02(0.01,0.03) | 0.17(0.11,0.24) | -3.90(-4.40,-3.40) |
| Grenada | 0.49(0.39,0.60) | 1.46(1.17,1.80) | 0.22(0.17,0.27) | 0.99(0.79,1.22) | -0.67(-0.99,-0.34) |
| Guam | 0.11(0.09,0.14) | 0.27(0.22,0.33) | 0.07(0.05,0.09) | 0.19(0.14,0.26) | 1.52(0.57,2.49) |
| Guatemala | 16.75(15.20,18.50) | 0.41(0.37,0.46) | 7.87(6.46,9.68) | 0.16(0.13,0.20) | -2.16(-2.46,-1.85) |
| Guinea | 48.16(32.12,66.84) | 1.75(1.17,2.43) | 49.81(30.90,98.71) | 0.82(0.51,1.63) | -1.86(-2.04,-1.69) |
| Guinea-Bissau | 8.13(5.11,12.08) | 1.69(1.06,2.50) | 6.98(4.32,10.97) | 0.78(0.48,1.22) | -2.23(-2.57,-1.89) |
| Guyana | 1.44(1.16,1.73) | 0.49(0.39,0.59) | 0.81(0.62,1.04) | 0.38(0.29,0.49) | 0.77(0.24,1.29) |
| Haiti | 76.38(21.40,122.88) | 2.82(0.79,4.53) | 65.39(28.43,105.51) | 1.50(0.65,2.42) | -1.57(-1.84,-1.30) |
| Honduras | 6.87(5.21,8.87) | 0.31(0.24,0.40) | 3.23(1.72,5.46) | 0.10(0.05,0.17) | -3.85(-4.10,-3.59) |
| Hungary | 7.86(6.62,9.30) | 0.37(0.31,0.44) | 2.18(1.80,2.68) | 0.16(0.13,0.19) | -1.89(-2.31,-1.48) |
| Iceland | 0.14(0.12,0.16) | 0.22(0.19,0.25) | 0.07(0.06,0.08) | 0.10(0.08,0.12) | -2.07(-2.75,-1.38) |
| India | 1979.21(1182.95,2650.46) | 0.61(0.36,0.81) | 807.21(631.39,1031.71) | 0.22(0.17,0.28) | -3.27(-3.50,-3.04) |
| Indonesia | 310.76(170.79,467.93) | 0.46(0.25,0.69) | 167.67(119.25,259.67) | 0.25(0.18,0.39) | -1.84(-1.89,-1.79) |
| Iran (Islamic Republic of) | 93.31(72.54,120.68) | 0.37(0.29,0.48) | 40.67(31.86,53.15) | 0.20(0.16,0.26) | -1.30(-1.83,-0.76) |
| Iraq | 68.65(40.42,97.35) | 0.83(0.49,1.18) | 40.83(28.44,57.27) | 0.30(0.21,0.43) | -2.79(-3.14,-2.44) |
| Ireland | 2.40(2.01,2.81) | 0.24(0.20,0.29) | 0.90(0.71,1.15) | 0.09(0.07,0.12) | -3.18(-3.57,-2.79) |
| Israel | 5.60(4.97,6.35) | 0.37(0.32,0.41) | 4.14(3.43,5.06) | 0.16(0.13,0.19) | -2.56(-2.97,-2.15) |
| Italy | 34.03(32.22,35.97) | 0.37(0.35,0.39) | 10.60(9.45,11.97) | 0.14(0.12,0.16) | -3.58(-3.82,-3.35) |
| Jamaica | 9.30(7.80,10.80) | 1.11(0.93,1.29) | 3.06(2.40,3.88) | 0.52(0.41,0.66) | -2.31(-2.66,-1.96) |
| Japan | 68.97(67.07,70.69) | 0.30(0.29,0.31) | 21.72(20.47,22.75) | 0.14(0.13,0.15) | -2.61(-2.85,-2.37) |
| Jordan | 10.64(7.93,14.32) | 0.65(0.49,0.88) | 15.65(11.59,21.23) | 0.43(0.32,0.58) | -1.68(-2.06,-1.29) |
| Kazakhstan | 35.32(30.88,39.95) | 0.68(0.59,0.77) | 9.62(7.79,11.80) | 0.18(0.14,0.22) | -4.40(-4.84,-3.96) |
| Kenya | 63.79(42.33,91.47) | 0.57(0.38,0.82) | 71.50(54.02,90.97) | 0.38(0.29,0.49) | -0.15(-0.65,0.36) |
| Kiribati | 0.03(0.02,0.05) | 0.11(0.06,0.16) | 0.04(0.02,0.06) | 0.09(0.04,0.14) | -1.01(-1.21,-0.82) |
| Kuwait | 4.11(3.54,4.80) | 0.74(0.64,0.87) | 1.97(1.53,2.43) | 0.23(0.18,0.29) | -1.85(-2.78,-0.92) |
| Kyrgyzstan | 6.06(4.79,7.95) | 0.36(0.29,0.47) | 3.97(2.98,5.03) | 0.17(0.13,0.22) | -2.30(-2.82,-1.78) |
| Lao People's Democratic Republic | 13.71(4.75,21.55) | 0.74(0.26,1.17) | 7.79(5.04,12.40) | 0.34(0.22,0.54) | -2.60(-2.67,-2.53) |
| Latvia | 1.19(1.02,1.37) | 0.21(0.18,0.24) | 0.49(0.40,0.60) | 0.16(0.13,0.20) | 0.96(0.23,1.70) |
| Lebanon | 6.46(4.49,8.71) | 0.62(0.43,0.83) | 3.67(2.56,5.19) | 0.29(0.20,0.41) | -2.58(-2.91,-2.25) |
| Lesotho | 1.83(1.23,2.77) | 0.27(0.18,0.41) | 3.03(1.94,4.40) | 0.48(0.31,0.70) | 2.86(2.49,3.23) |
| Liberia | 20.78(11.78,28.67) | 1.84(1.04,2.54) | 19.02(12.44,26.91) | 0.87(0.57,1.23) | -2.29(-2.98,-1.58) |
| Libya | 13.43(9.08,17.79) | 0.74(0.50,0.98) | 12.68(8.89,17.41) | 0.85(0.60,1.17) | 0.95(0.64,1.26) |
| Lithuania | 1.49(1.23,1.81) | 0.18(0.15,0.22) | 0.95(0.75,1.21) | 0.23(0.18,0.30) | 0.49(-0.12,1.10) |
| Luxembourg | 0.15(0.14,0.17) | 0.23(0.21,0.26) | 0.10(0.08,0.12) | 0.10(0.08,0.12) | -4.15(-4.66,-3.65) |
| Madagascar | 90.32(64.41,118.13) | 1.66(1.18,2.17) | 97.76(66.38,139.10) | 0.83(0.57,1.19) | -1.75(-1.96,-1.55) |
| Malawi | 283.60(205.69,374.14) | 6.23(4.52,8.22) | 260.70(131.72,542.44) | 3.21(1.62,6.68) | -1.79(-2.06,-1.51) |
| Malaysia | 22.73(16.05,30.12) | 0.35(0.24,0.46) | 14.02(9.82,18.51) | 0.18(0.13,0.24) | -1.61(-1.94,-1.27) |
| Maldives | 0.46(0.22,0.67) | 0.44(0.21,0.64) | 0.15(0.11,0.23) | 0.15(0.11,0.23) | -2.97(-3.17,-2.78) |
| Mali | 46.90(31.31,68.65) | 1.14(0.76,1.66) | 59.75(36.03,109.76) | 0.52(0.31,0.95) | -2.22(-2.39,-2.04) |
| Malta | 0.18(0.13,0.21) | 0.20(0.15,0.24) | 0.19(0.15,0.25) | 0.30(0.23,0.39) | -0.84(-1.63,-0.03) |
| Marshall Islands | 0.03(0.02,0.05) | 0.14(0.09,0.21) | 0.04(0.03,0.07) | 0.24(0.15,0.38) | 1.70(1.26,2.15) |
| Mauritania | 7.40(5.00,10.48) | 0.80(0.54,1.13) | 10.27(6.69,14.34) | 0.55(0.36,0.77) | -1.35(-1.63,-1.06) |
| Mauritius | 0.27(0.24,0.29) | 0.08(0.07,0.09) | 0.28(0.25,0.32) | 0.14(0.12,0.15) | 0.53(-0.11,1.17) |
| Mexico | 171.56(156.59,189.57) | 0.51(0.47,0.57) | 66.86(57.50,77.77) | 0.21(0.18,0.24) | -2.41(-2.72,-2.10) |
| Micronesia (Federated States of) | 0.11(0.07,0.16) | 0.23(0.15,0.35) | 0.06(0.04,0.10) | 0.21(0.14,0.33) | -0.18(-0.35,-0.01) |
| Monaco | 0.01(0.01,0.01) | 0.24(0.16,0.35) | 0.01(0.01,0.01) | 0.19(0.14,0.27) | -1.99(-2.50,-1.48) |
| Mongolia | 8.86(5.73,12.37) | 0.98(0.64,1.37) | 2.77(1.94,3.92) | 0.25(0.18,0.36) | -4.57(-5.09,-4.05) |
| Montenegro | 0.61(0.48,0.75) | 0.38(0.30,0.46) | 0.14(0.10,0.21) | 0.12(0.09,0.19) | -3.29(-3.91,-2.66) |
| Morocco | 54.91(35.39,77.41) | 0.56(0.36,0.79) | 23.15(15.69,34.23) | 0.24(0.16,0.35) | -2.37(-2.59,-2.15) |
| Mozambique | 21.67(11.84,53.57) | 0.35(0.19,0.86) | 20.69(10.12,49.83) | 0.15(0.07,0.35) | -2.36(-2.67,-2.05) |
| Myanmar | 126.37(44.06,197.53) | 0.86(0.30,1.34) | 59.54(38.63,92.68) | 0.38(0.25,0.59) | -2.85(-3.00,-2.70) |
| Namibia | 3.27(2.08,4.71) | 0.55(0.35,0.78) | 5.59(3.40,8.46) | 0.68(0.41,1.02) | 1.68(1.10,2.25) |
| Nauru | 0.01(0.01,0.02) | 0.31(0.21,0.44) | 0.02(0.01,0.02) | 0.40(0.25,0.59) | 0.70(0.22,1.17) |
| Nepal | 72.53(32.95,109.73) | 0.86(0.39,1.30) | 29.41(18.63,50.36) | 0.32(0.20,0.55) | -2.86(-3.10,-2.63) |
| Netherlands | 7.01(6.18,7.89) | 0.26(0.23,0.29) | 3.17(2.61,3.73) | 0.12(0.10,0.14) | -2.50(-2.77,-2.24) |
| New Zealand | 1.38(1.21,1.57) | 0.17(0.15,0.20) | 0.85(0.72,1.01) | 0.09(0.07,0.10) | -2.84(-3.84,-1.82) |
| Nicaragua | 9.30(7.05,12.04) | 0.51(0.39,0.66) | 2.92(2.21,3.89) | 0.15(0.11,0.20) | -3.11(-3.39,-2.83) |
| Niger | 76.46(43.07,111.70) | 1.88(1.06,2.75) | 89.88(52.79,141.17) | 0.70(0.41,1.11) | -3.18(-3.43,-2.93) |
| Nigeria | 708.11(444.85,987.26) | 1.81(1.14,2.52) | 1285.39(732.11,1831.87) | 1.27(0.72,1.80) | -0.89(-1.02,-0.76) |
| Niue | 0.00(0.00,0.00) | 0.23(0.15,0.33) | 0.01(0.00,0.01) | 1.54(1.04,2.30) | 2.59(1.31,3.90) |
| North Macedonia | 2.25(1.83,2.82) | 0.43(0.35,0.53) | 0.59(0.42,0.78) | 0.18(0.13,0.24) | -2.02(-2.58,-1.47) |
| Northern Mariana Islands | 0.01(0.01,0.02) | 0.07(0.04,0.14) | 0.01(0.01,0.02) | 0.11(0.07,0.17) | 2.76(1.93,3.60) |
| Norway | 2.41(2.25,2.60) | 0.30(0.28,0.33) | 0.69(0.60,0.79) | 0.07(0.06,0.09) | -4.13(-4.55,-3.70) |
| Oman | 3.63(2.36,5.21) | 0.43(0.28,0.62) | 2.61(1.91,3.38) | 0.21(0.16,0.28) | -1.13(-1.57,-0.69) |
| Pakistan | 567.15(409.30,751.55) | 1.15(0.83,1.53) | 1091.79(754.34,1552.66) | 1.28(0.88,1.82) | 0.78(0.60,0.95) |
| Palau | 0.01(0.00,0.01) | 0.11(0.08,0.16) | 0.00(0.00,0.00) | 0.08(0.05,0.13) | -0.90(-1.08,-0.72) |
| Palestine | 3.36(2.36,4.59) | 0.35(0.24,0.47) | 3.84(2.87,5.17) | 0.21(0.15,0.28) | -0.96(-1.40,-0.51) |
| Panama | 3.99(3.45,4.62) | 0.48(0.41,0.55) | 4.44(3.62,5.33) | 0.38(0.31,0.46) | -0.64(-0.84,-0.44) |
| Papua New Guinea | 4.77(2.65,7.43) | 0.28(0.16,0.44) | 12.44(7.61,19.38) | 0.32(0.19,0.49) | 0.59(0.34,0.84) |
| Paraguay | 8.08(6.11,10.51) | 0.48(0.37,0.63) | 6.50(4.60,9.20) | 0.32(0.23,0.46) | -0.97(-1.40,-0.54) |
| Peru | 67.44(55.01,88.54) | 0.81(0.66,1.07) | 36.35(26.49,48.95) | 0.38(0.28,0.51) | -2.19(-2.38,-2.00) |
| Philippines | 143.14(96.32,189.70) | 0.57(0.38,0.75) | 102.55(83.29,137.04) | 0.30(0.24,0.40) | -1.50(-1.68,-1.32) |
| Poland | 37.46(35.87,39.01) | 0.39(0.37,0.41) | 9.13(8.09,10.00) | 0.16(0.14,0.17) | -2.99(-3.40,-2.57) |
| Portugal | 9.08(7.86,10.43) | 0.43(0.37,0.49) | 1.89(1.53,2.27) | 0.14(0.11,0.17) | -4.59(-4.99,-4.18) |
| Puerto Rico | 5.28(4.64,6.03) | 0.53(0.47,0.61) | 0.66(0.55,0.79) | 0.15(0.12,0.18) | -3.57(-3.83,-3.32) |
| Qatar | 0.23(0.16,0.33) | 0.18(0.13,0.26) | 0.49(0.35,0.71) | 0.10(0.07,0.14) | -1.25(-1.76,-0.74) |
| Republic of Korea | 48.18(38.10,63.41) | 0.42(0.34,0.56) | 6.40(4.68,8.68) | 0.11(0.08,0.14) | -4.67(-4.93,-4.41) |
| Republic of Moldova | 11.67(10.00,13.47) | 0.94(0.81,1.09) | 1.70(1.41,2.06) | 0.33(0.27,0.39) | -2.38(-2.88,-1.88) |
| Romania | 33.95(29.95,38.16) | 0.61(0.54,0.69) | 6.00(5.13,7.07) | 0.20(0.17,0.23) | -3.73(-4.09,-3.38) |
| Russian Federation | 242.75(234.85,250.45) | 0.70(0.68,0.72) | 43.08(39.97,46.00) | 0.17(0.15,0.18) | -4.91(-5.37,-4.44) |
| Rwanda | 97.62(65.41,135.98) | 2.88(1.93,4.01) | 53.65(36.53,88.32) | 1.08(0.73,1.78) | -3.72(-4.03,-3.42) |
| Saint Kitts and Nevis | 0.11(0.10,0.12) | 0.78(0.69,0.88) | 0.05(0.04,0.06) | 0.52(0.44,0.61) | -1.02(-1.23,-0.82) |
| Saint Lucia | 0.44(0.38,0.53) | 0.86(0.73,1.02) | 0.19(0.15,0.24) | 0.64(0.51,0.79) | -1.37(-1.62,-1.12) |
| Saint Vincent and the Grenadines | 0.54(0.45,0.63) | 1.31(1.10,1.54) | 0.23(0.19,0.27) | 0.90(0.77,1.08) | -0.96(-1.33,-0.59) |
| Samoa | 0.32(0.22,0.45) | 0.45(0.30,0.64) | 0.24(0.15,0.41) | 0.30(0.19,0.51) | -1.28(-1.39,-1.17) |
| San Marino | 0.03(0.02,0.04) | 0.72(0.52,0.96) | 0.01(0.01,0.01) | 0.23(0.16,0.32) | -3.36(-3.49,-3.23) |
| Sao Tome and Principe | 0.75(0.53,1.07) | 1.32(0.94,1.88) | 0.34(0.20,0.57) | 0.44(0.26,0.73) | -3.07(-3.48,-2.65) |
| Saudi Arabia | 31.17(17.80,44.93) | 0.48(0.27,0.69) | 11.24(6.76,16.13) | 0.15(0.09,0.21) | -3.74(-3.83,-3.64) |
| Senegal | 47.70(32.48,66.64) | 1.31(0.89,1.83) | 39.22(26.76,58.64) | 0.62(0.42,0.92) | -2.36(-2.61,-2.12) |
| Serbia | 11.52(7.70,14.86) | 0.53(0.36,0.69) | 1.59(1.16,2.28) | 0.12(0.09,0.17) | -5.13(-5.64,-4.62) |
| Seychelles | 0.05(0.04,0.07) | 0.21(0.16,0.30) | 0.03(0.02,0.04) | 0.13(0.09,0.17) | 0.05(-0.92,1.04) |
| Sierra Leone | 31.09(18.74,44.05) | 1.72(1.03,2.43) | 33.29(23.04,48.27) | 0.93(0.64,1.35) | -1.89(-2.05,-1.73) |
| Singapore | 2.27(1.93,2.73) | 0.35(0.30,0.42) | 1.13(0.93,1.36) | 0.14(0.12,0.17) | -2.47(-2.81,-2.13) |
| Slovakia | 4.62(3.66,5.96) | 0.35(0.28,0.45) | 1.65(1.24,2.25) | 0.19(0.14,0.26) | -1.87(-2.12,-1.61) |
| Slovenia | 1.31(1.13,1.48) | 0.32(0.27,0.36) | 0.46(0.37,0.58) | 0.15(0.12,0.18) | -1.67(-2.12,-1.23) |
| Solomon Islands | 0.24(0.13,0.38) | 0.16(0.08,0.25) | 0.51(0.34,0.78) | 0.20(0.13,0.30) | 0.91(0.62,1.21) |
| Somalia | 64.28(34.92,94.42) | 1.65(0.90,2.42) | 90.18(45.84,143.14) | 0.87(0.44,1.39) | -1.63(-2.01,-1.25) |
| South Africa | 44.08(30.15,65.90) | 0.32(0.22,0.48) | 48.57(39.72,60.58) | 0.32(0.26,0.40) | -0.31(-0.67,0.05) |
| South Sudan | 54.92(31.92,78.53) | 2.09(1.22,2.99) | 82.23(50.74,116.56) | 1.91(1.18,2.71) | 0.14(-0.57,0.86) |
| Spain | 25.05(21.26,28.93) | 0.32(0.27,0.37) | 8.08(6.45,10.09) | 0.12(0.10,0.16) | -3.53(-3.75,-3.30) |
| Sri Lanka | 16.09(11.77,20.90) | 0.29(0.21,0.38) | 6.21(4.09,8.83) | 0.12(0.08,0.17) | -2.79(-3.36,-2.21) |
| Sudan | 92.01(36.99,182.62) | 1.03(0.42,2.05) | 73.35(48.25,103.49) | 0.44(0.29,0.62) | -2.43(-2.55,-2.31) |
| Suriname | 0.98(0.58,1.26) | 0.75(0.45,0.97) | 0.73(0.50,0.99) | 0.51(0.35,0.69) | -1.00(-1.33,-0.67) |
| Sweden | 2.74(2.45,3.09) | 0.18(0.16,0.20) | 1.30(1.11,1.52) | 0.07(0.06,0.08) | -2.03(-2.68,-1.36) |
| Switzerland | 3.06(2.69,3.52) | 0.26(0.23,0.30) | 1.28(1.09,1.51) | 0.10(0.08,0.11) | -3.65(-3.97,-3.33) |
| Syrian Arab Republic | 23.30(17.08,29.78) | 0.39(0.29,0.50) | 7.67(5.74,10.37) | 0.21(0.16,0.28) | -1.97(-2.60,-1.33) |
| Taiwan (Province of China) | 22.24(19.84,24.65) | 0.40(0.36,0.45) | 5.40(4.60,6.39) | 0.18(0.16,0.22) | -2.53(-2.90,-2.16) |
| Tajikistan | 29.20(13.23,42.83) | 1.26(0.57,1.84) | 25.36(17.09,36.89) | 0.71(0.48,1.03) | -2.45(-2.84,-2.05) |
| Thailand | 88.25(67.85,117.32) | 0.52(0.40,0.70) | 21.85(16.18,34.35) | 0.22(0.17,0.35) | -3.39(-3.79,-2.98) |
| Timor-Leste | 2.03(0.76,3.20) | 0.61(0.23,0.96) | 1.32(0.93,2.03) | 0.25(0.18,0.39) | -2.98(-3.40,-2.56) |
| Togo | 17.85(12.43,25.31) | 1.01(0.71,1.44) | 22.37(14.78,34.16) | 0.68(0.45,1.03) | -1.17(-1.31,-1.02) |
| Tokelau | 0.00(0.00,0.00) | 0.23(0.15,0.35) | 0.01(0.00,0.01) | 1.34(0.84,2.09) | 1.48(-0.22,3.22) |
| Tonga | 0.23(0.15,0.33) | 0.56(0.37,0.79) | 0.23(0.14,0.38) | 0.59(0.37,0.97) | -0.20(-0.63,0.24) |
| Trinidad and Tobago | 3.33(2.91,3.79) | 0.82(0.72,0.93) | 1.08(0.86,1.37) | 0.40(0.31,0.50) | -1.91(-2.32,-1.50) |
| Tunisia | 28.35(19.95,36.89) | 0.91(0.64,1.19) | 9.67(6.90,13.23) | 0.35(0.25,0.48) | -2.77(-2.88,-2.66) |
| Turkey | 154.72(94.18,210.62) | 0.76(0.46,1.03) | 47.50(35.76,62.42) | 0.26(0.19,0.34) | -3.43(-3.71,-3.14) |
| Turkmenistan | 3.86(3.33,4.47) | 0.26(0.22,0.30) | 2.64(2.17,3.22) | 0.17(0.14,0.21) | -1.81(-2.30,-1.31) |
| Tuvalu | 0.01(0.01,0.02) | 0.32(0.18,0.47) | 0.01(0.01,0.01) | 0.19(0.14,0.28) | -1.23(-1.39,-1.06) |
| Uganda | 210.08(144.27,292.45) | 2.50(1.71,3.47) | 445.76(270.18,722.69) | 2.25(1.36,3.64) | -0.18(-0.43,0.06) |
| Ukraine | 41.78(29.13,51.48) | 0.37(0.26,0.45) | 16.75(13.91,20.22) | 0.26(0.22,0.32) | -1.31(-1.73,-0.89) |
| United Arab Emirates | 1.69(1.07,2.57) | 0.29(0.18,0.44) | 1.45(1.06,2.00) | 0.11(0.08,0.15) | -2.74(-3.09,-2.39) |
| United Kingdom | 20.75(20.13,21.37) | 0.19(0.18,0.20) | 9.97(9.30,10.54) | 0.08(0.08,0.09) | -2.60(-2.90,-2.31) |
| United Republic of Tanzania | 269.97(186.17,359.59) | 2.24(1.54,2.98) | 333.32(220.07,512.39) | 1.37(0.90,2.10) | -1.03(-1.31,-0.76) |
| United States Virgin Islands | 0.20(0.15,0.27) | 0.63(0.47,0.85) | 0.02(0.01,0.03) | 0.16(0.10,0.25) | -3.46(-3.92,-3.00) |
| United States of America | 105.77(103.53,108.28) | 0.19(0.19,0.19) | 42.87(39.86,45.77) | 0.07(0.07,0.08) | -3.19(-3.36,-3.03) |
| Uruguay | 3.42(2.93,3.98) | 0.42(0.36,0.49) | 1.55(1.26,1.92) | 0.24(0.19,0.29) | -2.25(-2.79,-1.71) |
| Uzbekistan | 52.00(36.35,67.64) | 0.61(0.42,0.79) | 42.68(33.53,54.55) | 0.42(0.33,0.54) | -0.96(-1.25,-0.68) |
| Vanuatu | 0.09(0.05,0.14) | 0.13(0.07,0.20) | 0.19(0.12,0.28) | 0.16(0.11,0.24) | 1.15(0.72,1.58) |
| Venezuela (Bolivarian Republic of) | 36.42(33.96,39.08) | 0.51(0.48,0.55) | 26.56(20.83,33.08) | 0.40(0.31,0.50) | -0.76(-1.23,-0.29) |
| Viet Nam | 125.53(83.90,171.37) | 0.47(0.32,0.65) | 75.02(49.52,106.91) | 0.30(0.20,0.43) | -1.14(-1.29,-0.99) |
| Yemen | 32.70(18.28,61.87) | 0.46(0.26,0.87) | 34.70(22.84,54.89) | 0.25(0.17,0.40) | -1.85(-1.98,-1.72) |
| Zambia | 91.12(62.94,120.94) | 2.43(1.68,3.22) | 99.18(59.60,165.15) | 1.20(0.72,2.00) | -2.13(-2.28,-1.98) |
| Zimbabwe | 22.28(10.81,32.19) | 0.46(0.22,0.67) | 67.60(30.88,97.37) | 1.07(0.49,1.55) | 5.16(4.00,6.34) |
| ^a^EAPC is expressed as 95% CIs | | | | | |

**Table S5** DALYs of Hodgkin lymphoma in Children Between 1990 and 2021 at 204 Countries and Territories.

| Location | Rate per 100000 (95% UI) | | | | |
| --- | --- | --- | --- | --- | --- |
|  | 1990 | | 2021 | | EAPC^a^ |
|  | Cases | Rates | Cases | Rates |  |
| Global | 217338.96(121249.10,280191.02) | 12.50(6.97,16.11) | 156611.11(85731.20,211399.21) | 7.78(4.26,10.51) | -1.40(-1.47,-1.33) |
| 5 SDI | | | | | |
| High SDI | 4565.97(4027.24,5087.24) | 2.46(2.17,2.74) | 1024.15(865.27,1204.00) | 0.59(0.50,0.70) | -4.57(-4.63,-4.51) |
| High-middle SDI | 21585.71(14297.77,26639.06) | 7.89(5.23,9.74) | 3854.77(2978.29,5025.52) | 1.67(1.29,2.18) | -5.12(-5.27,-4.97) |
| Middle SDI | 54174.05(29816.09,68918.38) | 9.39(5.17,11.94) | 19130.70(10885.21,24928.46) | 3.37(1.92,4.40) | -3.12(-3.25,-2.99) |
| Low-middle SDI | 76812.63(41552.90,105781.80) | 16.27(8.80,22.41) | 56519.91(34179.98,82369.89) | 9.75(5.89,14.21) | -1.51(-1.59,-1.42) |
| Low SDI | 60101.92(28945.66,88692.85) | 26.26(12.64,38.75) | 76021.84(33846.99,109900.11) | 16.52(7.35,23.88) | -1.50(-1.59,-1.41) |
| 21 GBD Region | | | | | |
| Andean Latin America | 1149.70(781.48,1573.20) | 7.74(5.26,10.59) | 570.46(391.65,875.75) | 3.15(2.16,4.84) | -2.72(-2.93,-2.51) |
| Australasia | 101.34(83.10,123.20) | 2.21(1.81,2.69) | 30.15(22.33,41.05) | 0.53(0.39,0.72) | -4.23(-4.91,-3.55) |
| Caribbean | 552.42(401.90,739.54) | 4.84(3.52,6.48) | 331.20(203.36,496.67) | 2.88(1.77,4.32) | -1.62(-1.75,-1.49) |
| Central Asia | 2417.36(1945.57,2871.09) | 9.67(7.79,11.49) | 1320.79(1062.44,1666.68) | 4.77(3.84,6.02) | -2.29(-2.64,-1.94) |
| Central Europe | 2424.49(2123.88,2715.42) | 8.22(7.20,9.21) | 222.12(186.45,282.87) | 1.25(1.05,1.60) | -6.29(-6.58,-5.99) |
| Central Latin America | 8956.07(8376.52,9617.47) | 13.91(13.01,14.94) | 2295.85(2002.07,2658.38) | 3.62(3.15,4.19) | -3.82(-4.09,-3.55) |
| Central Sub-Saharan Africa | 915.49(495.59,1677.21) | 3.62(1.96,6.63) | 1324.37(863.53,2141.28) | 2.26(1.47,3.65) | -1.25(-1.38,-1.12) |
| East Asia | 28849.79(10616.53,41759.95) | 8.75(3.22,12.66) | 2970.97(1922.13,4730.08) | 1.11(0.72,1.77) | -7.14(-7.40,-6.88) |
| Eastern Europe | 5150.49(4840.85,5480.00) | 10.01(9.41,10.65) | 784.84(718.03,880.32) | 2.21(2.03,2.48) | -5.07(-5.27,-4.88) |
| Eastern Sub-Saharan Africa | 29932.02(18917.83,43616.01) | 33.05(20.89,48.16) | 35714.86(18058.87,57869.48) | 20.02(10.12,32.43) | -1.52(-1.61,-1.43) |
| High-income Asia Pacific | 168.87(137.87,226.92) | 0.48(0.39,0.64) | 40.32(35.04,48.39) | 0.18(0.16,0.22) | -2.83(-3.11,-2.55) |
| High-income North America | 1152.22(1099.67,1210.62) | 1.87(1.78,1.96) | 315.39(292.71,340.21) | 0.48(0.45,0.52) | -4.58(-4.74,-4.41) |
| North Africa and Middle East | 13105.63(7883.82,18011.51) | 9.33(5.61,12.82) | 8140.08(4960.87,11452.03) | 4.44(2.71,6.25) | -2.10(-2.30,-1.89) |
| Oceania | 38.96(18.61,59.57) | 1.45(0.69,2.22) | 54.98(20.90,91.12) | 1.08(0.41,1.79) | -0.68(-0.82,-0.55) |
| South Asia | 86040.70(43825.52,122692.20) | 19.85(10.11,28.31) | 58465.56(36816.34,88168.46) | 11.53(7.26,17.39) | -1.64(-1.75,-1.54) |
| Southeast Asia | 5193.79(3469.33,8021.60) | 3.04(2.03,4.70) | 2277.18(1570.82,4152.25) | 1.32(0.91,2.40) | -2.74(-2.82,-2.65) |
| Southern Latin America | 683.63(541.31,877.02) | 4.58(3.63,5.88) | 260.21(200.43,339.20) | 1.80(1.38,2.34) | -2.69(-2.98,-2.40) |
| Southern Sub-Saharan Africa | 784.85(494.75,1099.44) | 3.79(2.39,5.31) | 1110.49(642.68,1555.09) | 4.61(2.67,6.46) | 0.73(0.52,0.94) |
| Tropical Latin America | 3749.62(3283.90,4241.20) | 6.99(6.13,7.91) | 1000.21(829.49,1174.48) | 1.99(1.65,2.34) | -3.34(-3.70,-2.99) |
| Western Europe | 2177.36(2017.68,2353.12) | 3.07(2.84,3.31) | 565.08(496.98,649.00) | 0.83(0.73,0.95) | -3.94(-4.15,-3.73) |
| Western Sub-Saharan Africa | 23794.15(4520.10,40822.57) | 27.08(5.14,46.45) | 38816.00(8031.50,70737.72) | 18.07(3.74,32.94) | -1.38(-1.51,-1.26) |
| 204 countries and territories | | | | | |
| Afghanistan | 572.49(288.82,1145.91) | 13.29(6.70,26.60) | 1373.65(849.98,2223.73) | 9.67(5.99,15.66) | -1.08(-2.77,0.63) |
| Albania | 99.22(39.87,162.50) | 8.88(3.57,14.54) | 10.37(5.54,17.88) | 2.34(1.25,4.03) | -2.87(-5.06,-0.63) |
| Algeria | 2821.07(1183.83,4726.67) | 26.30(11.04,44.07) | 1156.25(585.36,1949.39) | 8.69(4.40,14.66) | -2.99(-4.38,-1.59) |
| American Samoa | 0.01(0.00,0.02) | 0.05(0.02,0.09) | 0.01(0.00,0.02) | 0.07(0.02,0.15) | 2.47(-5.36,10.95) |
| Andorra | 0.15(0.09,0.25) | 1.62(0.98,2.68) | 0.07(0.04,0.13) | 0.70(0.39,1.24) | -3.18(-7.65,1.49) |
| Angola | 212.39(92.59,439.72) | 4.50(1.96,9.33) | 333.56(187.60,566.36) | 2.19(1.23,3.71) | -2.80(-6.74,1.31) |
| Antigua and Barbuda | 0.12(0.09,0.17) | 0.64(0.50,0.93) | 0.07(0.06,0.08) | 0.39(0.33,0.48) | -1.53(-6.88,4.13) |
| Argentina | 573.96(453.35,743.00) | 5.66(4.47,7.33) | 198.08(151.39,257.45) | 1.95(1.49,2.53) | -2.40(-5.19,0.47) |
| Armenia | 66.88(39.74,82.77) | 6.41(3.81,7.93) | 15.19(12.15,19.28) | 2.56(2.05,3.26) | -0.89(-3.18,1.45) |
| Australia | 93.65(76.80,115.27) | 2.47(2.03,3.04) | 26.48(18.94,36.83) | 0.56(0.40,0.78) | -3.95(-7.13,-0.67) |
| Austria | 41.72(33.55,50.83) | 3.09(2.49,3.77) | 8.19(5.61,11.38) | 0.63(0.43,0.88) | -3.03(-5.92,-0.04) |
| Azerbaijan | 358.00(193.27,617.78) | 14.75(7.96,25.46) | 237.29(117.82,420.37) | 10.05(4.99,17.81) | -1.72(-3.01,-0.41) |
| Bahamas | 1.28(1.07,1.56) | 1.59(1.33,1.93) | 0.74(0.57,0.94) | 0.91(0.71,1.16) | -2.14(-6.47,2.39) |
| Bahrain | 12.23(7.55,19.98) | 7.49(4.62,12.24) | 8.77(5.44,13.85) | 2.95(1.84,4.67) | -2.62(-4.40,-0.80) |
| Bangladesh | 14020.32(7194.30,26268.59) | 28.66(14.71,53.71) | 5737.39(2999.42,12218.57) | 12.54(6.55,26.70) | -2.62(-4.17,-1.05) |
| Barbados | 1.55(1.18,1.91) | 2.49(1.90,3.06) | 0.49(0.34,0.68) | 1.05(0.73,1.45) | -1.41(-6.05,3.46) |
| Belarus | 217.54(152.11,324.17) | 9.05(6.33,13.49) | 36.10(24.12,53.01) | 2.29(1.53,3.36) | -1.08(-3.19,1.07) |
| Belgium | 44.28(31.72,59.94) | 2.45(1.76,3.32) | 15.67(10.28,23.10) | 0.82(0.54,1.21) | -2.89(-6.12,0.45) |
| Belize | 4.03(3.48,4.60) | 4.93(4.25,5.62) | 1.89(1.62,2.25) | 1.53(1.32,1.83) | -3.01(-5.85,-0.07) |
| Benin | 67.94(30.86,115.68) | 2.81(1.27,4.78) | 56.45(26.72,120.94) | 0.93(0.44,1.99) | -2.47(-7.82,3.19) |
| Bermuda | 0.27(0.21,0.35) | 2.28(1.76,2.95) | 0.08(0.06,0.11) | 0.98(0.75,1.26) | -2.08(-6.28,2.31) |
| Bhutan | 44.36(17.27,72.84) | 16.92(6.59,27.78) | 17.51(8.22,40.96) | 9.35(4.39,21.89) | -2.45(-4.33,-0.54) |
| Bolivia (Plurinational State of) | 457.83(231.78,778.86) | 17.05(8.63,29.00) | 204.29(103.98,382.06) | 5.86(2.98,10.96) | -3.21(-5.80,-0.55) |
| Bosnia and Herzegovina | 59.30(34.70,88.15) | 5.41(3.17,8.05) | 7.76(4.53,14.46) | 1.58(0.92,2.95) | -2.73(-5.24,-0.16) |
| Botswana | 27.24(16.13,46.37) | 4.61(2.73,7.85) | 24.65(13.49,45.08) | 3.53(1.93,6.46) | 0.63(-2.02,3.35) |
| Brazil | 3665.81(3209.75,4167.16) | 7.06(6.18,8.02) | 941.07(784.53,1110.17) | 1.95(1.63,2.30) | -3.10(-5.89,-0.22) |
| Brunei Darussalam | 1.36(0.74,2.62) | 1.50(0.82,2.89) | 0.74(0.47,1.24) | 0.79(0.50,1.31) | -1.02(-5.49,3.66) |
| Bulgaria | 192.86(155.63,231.16) | 11.11(8.96,13.31) | 18.78(14.76,23.86) | 1.92(1.51,2.44) | -3.94(-6.34,-1.49) |
| Burkina Faso | 131.14(59.09,241.88) | 2.78(1.25,5.13) | 112.55(49.46,248.08) | 1.09(0.48,2.39) | -1.96(-7.32,3.72) |
| Burundi | 910.49(489.32,1382.23) | 34.74(18.67,52.73) | 1147.09(466.78,2141.25) | 19.59(7.97,36.58) | -2.11(-4.30,0.13) |
| Cabo Verde | 0.15(0.06,0.33) | 0.09(0.04,0.21) | 0.05(0.02,0.11) | 0.04(0.02,0.08) | -0.10(-9.01,9.69) |
| Cambodia | 177.37(99.73,330.68) | 3.81(2.14,7.09) | 75.16(31.97,177.66) | 1.47(0.62,3.47) | -3.02(-6.53,0.62) |
| Cameroon | 123.78(56.70,208.31) | 2.54(1.16,4.27) | 119.53(58.99,238.27) | 0.89(0.44,1.77) | -2.09(-7.56,3.70) |
| Canada | 185.82(152.59,226.36) | 3.23(2.65,3.94) | 27.08(19.46,35.71) | 0.44(0.32,0.58) | -6.02(-8.99,-2.94) |
| Central African Republic | 58.95(27.16,117.62) | 4.82(2.22,9.62) | 86.74(42.70,145.23) | 3.80(1.87,6.36) | -0.88(-4.50,2.87) |
| Chad | 81.08(36.77,135.53) | 2.77(1.26,4.63) | 117.80(54.60,249.35) | 1.31(0.61,2.77) | -1.36(-6.44,4.00) |
| Chile | 72.28(55.28,94.23) | 1.82(1.39,2.37) | 50.23(38.17,67.45) | 1.38(1.05,1.85) | -1.70(-4.84,1.56) |
| China | 28583.23(10411.64,41454.63) | 8.98(3.27,13.02) | 2898.18(1873.63,4584.89) | 1.12(0.72,1.77) | -5.88(-8.90,-2.77) |
| Colombia | 1318.22(1098.97,1590.93) | 11.30(9.42,13.64) | 405.81(315.00,522.33) | 3.82(2.97,4.92) | -2.53(-4.92,-0.08) |
| Comoros | 57.75(26.65,97.65) | 27.15(12.53,45.91) | 46.31(22.86,87.93) | 19.29(9.52,36.61) | -1.24(-3.44,1.01) |
| Congo | 34.12(17.32,58.90) | 3.24(1.65,5.59) | 37.04(22.50,63.24) | 1.92(1.17,3.28) | -1.84(-5.56,2.03) |
| Cook Islands | 0.05(0.02,0.11) | 0.73(0.30,1.61) | 0.02(0.01,0.05) | 0.58(0.17,1.41) | -1.27(-4.97,2.58) |
| Costa Rica | 184.13(154.19,217.99) | 16.38(13.72,19.39) | 42.78(33.25,54.48) | 4.20(3.27,5.36) | -2.79(-4.84,-0.69) |
| Croatia | 45.87(36.70,56.92) | 4.65(3.72,5.77) | 4.12(2.91,5.77) | 0.69(0.49,0.97) | -3.62(-7.02,-0.10) |
| Cuba | 177.41(123.05,234.96) | 7.09(4.91,9.38) | 43.55(30.26,60.31) | 2.45(1.70,3.39) | -2.70(-5.49,0.16) |
| Cyprus | 6.05(2.81,10.10) | 3.06(1.42,5.10) | 1.79(1.01,3.04) | 0.82(0.46,1.39) | -3.40(-6.40,-0.30) |
| Czechia | 178.61(133.59,235.33) | 8.11(6.06,10.68) | 17.76(11.71,26.00) | 1.03(0.68,1.52) | -4.28(-6.88,-1.60) |
| Côte d'Ivoire | 587.81(195.54,1059.42) | 10.31(3.43,18.58) | 853.50(302.28,1577.39) | 7.38(2.61,13.63) | -0.52(-3.85,2.93) |
| Democratic People's Republic of Korea | 204.01(99.28,363.21) | 3.43(1.67,6.11) | 66.03(26.62,153.15) | 1.38(0.56,3.21) | -2.47(-5.72,0.90) |
| Democratic Republic of the Congo | 591.37(296.50,1024.48) | 3.34(1.67,5.79) | 847.03(520.71,1389.35) | 2.23(1.37,3.66) | -1.68(-5.29,2.06) |
| Denmark | 27.01(22.41,32.12) | 3.06(2.54,3.64) | 3.83(2.94,4.89) | 0.40(0.31,0.51) | -4.71(-7.75,-1.57) |
| Djibouti | 44.49(25.06,74.67) | 25.55(14.39,42.88) | 69.63(32.71,129.17) | 16.85(7.92,31.26) | -1.32(-3.42,0.82) |
| Dominica | 0.55(0.33,0.88) | 2.23(1.33,3.56) | 0.41(0.23,0.64) | 3.01(1.69,4.70) | 1.39(-3.22,6.22) |
| Dominican Republic | 19.00(10.56,43.14) | 0.70(0.39,1.60) | 12.22(5.16,26.24) | 0.42(0.18,0.89) | -2.55(-7.86,3.07) |
| Ecuador | 210.86(154.57,291.22) | 5.46(4.00,7.53) | 163.29(110.95,231.27) | 3.22(2.19,4.56) | -1.30(-4.16,1.63) |
| Egypt | 562.54(222.39,1456.98) | 2.54(1.00,6.57) | 321.33(107.91,925.03) | 0.87(0.29,2.51) | -3.35(-6.61,0.02) |
| El Salvador | 189.78(120.75,282.18) | 8.79(5.60,13.08) | 66.66(43.18,106.43) | 3.67(2.37,5.85) | -2.26(-4.19,-0.28) |
| Equatorial Guinea | 8.25(3.92,16.16) | 4.19(1.99,8.21) | 9.19(4.00,20.75) | 1.57(0.68,3.55) | -3.60(-7.32,0.28) |
| Eritrea | 512.00(293.38,810.64) | 32.16(18.43,50.92) | 600.67(283.41,1066.30) | 23.79(11.23,42.24) | -1.12(-3.07,0.87) |
| Estonia | 33.07(23.02,45.30) | 9.47(6.59,12.98) | 2.68(1.80,3.81) | 1.24(0.83,1.76) | -3.08(-6.12,0.05) |
| Eswatini | 20.76(12.36,35.41) | 5.38(3.20,9.18) | 19.99(11.44,34.72) | 4.84(2.77,8.42) | 0.23(-2.46,3.01) |
| Ethiopia | 11768.40(5959.16,20248.49) | 48.30(24.46,83.11) | 10471.28(5820.78,19624.74) | 23.61(13.12,44.25) | -2.94(-4.70,-1.14) |
| Fiji | 9.67(4.06,20.97) | 3.43(1.44,7.45) | 13.92(5.97,25.29) | 5.11(2.19,9.28) | 2.08(0.12,4.08) |
| Finland | 26.12(21.42,32.09) | 2.71(2.22,3.33) | 4.70(3.30,6.40) | 0.55(0.39,0.76) | -3.75(-7.05,-0.33) |
| France | 276.42(218.52,352.74) | 2.36(1.87,3.01) | 72.77(47.78,106.32) | 0.63(0.41,0.92) | -3.43(-6.84,0.10) |
| Gabon | 10.43(5.83,17.65) | 2.56(1.43,4.33) | 10.80(6.22,17.89) | 1.69(0.97,2.80) | -0.70(-4.57,3.32) |
| Gambia | 42.94(12.83,84.83) | 9.31(2.78,18.39) | 57.10(23.56,111.30) | 5.75(2.37,11.20) | -1.72(-5.19,1.87) |
| Georgia | 252.68(127.81,366.14) | 18.46(9.34,26.75) | 36.68(24.53,50.44) | 4.98(3.33,6.85) | -3.81(-5.23,-2.37) |
| Germany | 362.06(299.87,439.68) | 2.80(2.32,3.40) | 92.12(66.36,125.59) | 0.77(0.55,1.05) | -2.78(-5.60,0.12) |
| Ghana | 23.83(7.26,52.14) | 0.35(0.11,0.78) | 10.67(3.86,26.15) | 0.08(0.03,0.20) | -5.89(-13.45,2.34) |
| Greece | 116.38(99.98,132.50) | 5.75(4.94,6.55) | 24.77(20.34,30.30) | 1.78(1.46,2.17) | -2.33(-3.61,-1.03) |
| Greenland | 0.50(0.10,1.00) | 3.52(0.70,7.04) | 0.08(0.04,0.12) | 0.65(0.38,1.06) | -4.59(-8.43,-0.59) |
| Grenada | 0.77(0.54,1.21) | 2.30(1.63,3.61) | 0.26(0.18,0.37) | 1.20(0.83,1.68) | -1.40(-6.68,4.18) |
| Guam | 0.81(0.42,1.27) | 1.93(1.01,3.04) | 0.51(0.21,0.93) | 1.39(0.57,2.55) | 0.38(-3.79,4.73) |
| Guatemala | 338.88(192.50,429.65) | 8.34(4.74,10.58) | 112.88(92.78,135.03) | 2.29(1.88,2.74) | -2.70(-4.82,-0.53) |
| Guinea | 505.14(132.85,1003.10) | 18.36(4.83,36.45) | 643.68(217.63,1423.41) | 10.65(3.60,23.54) | -1.69(-4.35,1.04) |
| Guinea-Bissau | 18.65(8.44,31.87) | 3.87(1.75,6.61) | 10.46(4.34,23.57) | 1.16(0.48,2.62) | -3.39(-8.36,1.85) |
| Guyana | 10.51(8.85,12.15) | 3.58(3.01,4.13) | 4.52(3.63,5.71) | 2.12(1.70,2.67) | 0.17(-3.21,3.66) |
| Haiti | 207.53(92.98,374.09) | 7.65(3.43,13.79) | 239.57(117.69,399.72) | 5.50(2.70,9.18) | -1.23(-5.48,3.21) |
| Honduras | 130.40(63.46,197.34) | 5.90(2.87,8.93) | 57.87(29.03,106.26) | 1.77(0.89,3.24) | -3.90(-5.93,-1.84) |
| Hungary | 108.07(75.49,143.69) | 5.07(3.54,6.74) | 8.20(5.53,11.69) | 0.59(0.40,0.84) | -3.92(-7.05,-0.67) |
| Iceland | 2.04(1.57,2.56) | 3.21(2.47,4.04) | 0.47(0.32,0.72) | 0.70(0.47,1.07) | -3.35(-6.76,0.20) |
| India | 51097.82(24635.89,71870.02) | 15.65(7.54,22.01) | 20890.97(12764.55,33041.34) | 5.70(3.48,9.02) | -3.20(-4.78,-1.59) |
| Indonesia | 1881.63(1263.42,3583.21) | 2.78(1.87,5.29) | 802.05(405.62,1920.37) | 1.19(0.60,2.85) | -2.24(-5.86,1.52) |
| Iran (Islamic Republic of) | 1267.59(890.07,2072.50) | 4.99(3.51,8.16) | 733.72(336.77,1071.73) | 3.64(1.67,5.31) | -0.59(-2.95,1.82) |
| Iraq | 1072.29(430.68,1763.22) | 13.02(5.23,21.41) | 639.99(284.83,1182.60) | 4.75(2.12,8.78) | -2.92(-5.16,-0.64) |
| Ireland | 29.12(22.53,37.90) | 2.96(2.29,3.86) | 5.69(4.09,7.60) | 0.57(0.41,0.76) | -4.28(-7.24,-1.22) |
| Israel | 38.97(31.52,48.59) | 2.54(2.06,3.17) | 17.58(13.40,22.95) | 0.67(0.51,0.87) | -3.12(-6.79,0.69) |
| Italy | 397.48(367.17,440.23) | 4.31(3.98,4.77) | 107.59(93.49,125.47) | 1.42(1.23,1.65) | -3.35(-6.37,-0.23) |
| Jamaica | 55.16(42.04,69.98) | 6.60(5.03,8.38) | 5.28(3.78,7.32) | 0.90(0.65,1.25) | -4.41(-8.95,0.35) |
| Japan | 77.96(75.02,82.08) | 0.34(0.32,0.36) | 27.57(25.84,29.58) | 0.18(0.17,0.19) | -2.04(-7.84,4.11) |
| Jordan | 56.71(36.34,95.10) | 3.47(2.22,5.82) | 73.65(45.18,110.71) | 2.03(1.24,3.05) | -1.60(-5.45,2.42) |
| Kazakhstan | 311.17(261.95,373.72) | 5.99(5.04,7.19) | 118.52(95.55,145.70) | 2.18(1.76,2.68) | -3.91(-6.37,-1.38) |
| Kenya | 917.31(582.09,1500.01) | 8.21(5.21,13.43) | 1224.38(652.30,2519.78) | 6.56(3.49,13.50) | 0.06(-2.15,2.33) |
| Kiribati | 0.27(0.06,0.59) | 0.90(0.22,2.01) | 0.33(0.07,0.80) | 0.80(0.17,1.91) | -0.66(-3.80,2.58) |
| Kuwait | 78.69(62.89,97.67) | 14.20(11.35,17.62) | 11.51(8.22,15.19) | 1.36(0.97,1.80) | -2.17(-5.92,1.73) |
| Kyrgyzstan | 244.56(172.05,341.60) | 14.58(10.26,20.36) | 56.61(37.44,78.50) | 2.49(1.65,3.45) | -3.06(-5.20,-0.88) |
| Lao People's Democratic Republic | 78.75(38.78,162.17) | 4.27(2.10,8.80) | 36.69(15.82,86.72) | 1.60(0.69,3.78) | -2.90(-6.51,0.84) |
| Latvia | 51.44(36.00,69.44) | 9.04(6.33,12.20) | 2.72(1.67,4.01) | 0.92(0.56,1.35) | -2.53(-5.06,0.06) |
| Lebanon | 146.16(71.68,244.93) | 13.97(6.85,23.42) | 66.20(34.12,113.82) | 5.18(2.67,8.91) | -2.95(-4.93,-0.93) |
| Lesotho | 31.13(17.16,52.85) | 4.56(2.51,7.74) | 37.06(18.78,66.65) | 5.88(2.98,10.57) | 2.12(-0.22,4.51) |
| Liberia | 32.68(14.56,54.51) | 2.89(1.29,4.82) | 24.19(11.00,50.37) | 1.11(0.50,2.30) | -3.26(-8.40,2.18) |
| Libya | 324.25(178.71,514.27) | 17.90(9.87,28.40) | 207.65(110.64,326.20) | 13.92(7.42,21.87) | 0.24(-1.92,2.44) |
| Lithuania | 68.25(51.94,88.93) | 8.22(6.25,10.71) | 6.63(4.55,9.71) | 1.63(1.12,2.38) | -2.30(-4.58,0.03) |
| Luxembourg | 1.11(0.95,1.26) | 1.68(1.44,1.91) | 0.35(0.27,0.44) | 0.35(0.27,0.44) | -5.03(-8.92,-0.98) |
| Madagascar | 1341.23(761.99,2149.12) | 24.58(13.97,39.39) | 1948.93(1006.48,3333.85) | 16.61(8.58,28.41) | -1.38(-3.47,0.75) |
| Malawi | 1048.75(509.42,1762.54) | 23.05(11.20,38.74) | 919.95(349.63,1817.26) | 11.32(4.30,22.37) | -2.01(-6.26,2.42) |
| Malaysia | 227.31(76.50,418.14) | 3.46(1.16,6.36) | 110.93(45.70,195.03) | 1.46(0.60,2.56) | -2.21(-5.32,1.00) |
| Maldives | 3.69(2.10,6.01) | 3.51(2.00,5.73) | 1.13(0.66,1.91) | 1.13(0.66,1.91) | -3.38(-6.64,-0.01) |
| Mali | 930.42(222.37,1920.79) | 22.53(5.38,46.51) | 1297.49(445.41,2763.80) | 11.21(3.85,23.88) | -2.15(-4.13,-0.13) |
| Malta | 2.46(1.81,3.09) | 2.81(2.06,3.53) | 1.48(1.07,2.04) | 2.31(1.67,3.19) | -1.60(-4.56,1.45) |
| Marshall Islands | 0.17(0.05,0.38) | 0.76(0.24,1.74) | 0.13(0.04,0.26) | 0.75(0.25,1.50) | 0.70(-3.44,5.02) |
| Mauritania | 21.13(10.41,35.13) | 2.29(1.13,3.80) | 12.86(5.70,27.89) | 0.69(0.31,1.50) | -2.97(-7.96,2.30) |
| Mauritius | 8.00(7.20,9.01) | 2.43(2.18,2.73) | 2.69(2.27,3.10) | 1.30(1.10,1.49) | -1.52(-4.09,1.12) |
| Mexico | 6092.82(5647.92,6625.61) | 18.23(16.90,19.83) | 1203.59(1056.97,1385.76) | 3.75(3.30,4.32) | -3.45(-5.38,-1.47) |
| Micronesia (Federated States of) | 0.50(0.17,1.04) | 1.08(0.37,2.27) | 0.19(0.06,0.38) | 0.61(0.20,1.25) | -1.05(-5.31,3.40) |
| Monaco | 0.16(0.09,0.31) | 4.68(2.51,8.74) | 0.15(0.06,0.39) | 2.98(1.29,7.77) | -2.14(-4.45,0.22) |
| Mongolia | 66.51(29.71,136.34) | 7.39(3.30,15.15) | 47.09(27.49,76.36) | 4.33(2.53,7.03) | -3.23(-5.77,-0.63) |
| Montenegro | 12.41(7.88,17.97) | 7.68(4.88,11.12) | 3.00(1.96,5.04) | 2.69(1.75,4.52) | -3.41(-5.31,-1.47) |
| Morocco | 1457.68(703.00,2466.90) | 14.90(7.18,25.21) | 546.58(267.30,981.52) | 5.58(2.73,10.02) | -2.50(-4.14,-0.84) |
| Mozambique | 2548.71(1394.55,3904.08) | 41.08(22.48,62.93) | 3375.47(1299.30,6214.81) | 23.66(9.11,43.56) | -1.84(-2.60,-1.07) |
| Myanmar | 655.49(310.90,1340.82) | 4.44(2.10,9.07) | 245.21(104.59,592.17) | 1.57(0.67,3.79) | -3.29(-7.05,0.63) |
| Namibia | 43.52(19.36,71.73) | 7.24(3.22,11.94) | 54.48(25.45,95.88) | 6.60(3.08,11.62) | 0.84(-1.71,3.46) |
| Nauru | 0.04(0.01,0.08) | 0.89(0.30,1.87) | 0.03(0.01,0.06) | 0.76(0.27,1.57) | 0.16(-4.70,5.28) |
| Nepal | 1852.03(915.12,3504.41) | 21.98(10.86,41.59) | 830.52(376.47,1953.07) | 9.00(4.08,21.17) | -2.76(-4.24,-1.25) |
| Netherlands | 117.36(95.09,141.09) | 4.31(3.49,5.18) | 38.34(28.81,50.96) | 1.43(1.07,1.90) | -2.74(-5.32,-0.09) |
| New Zealand | 7.69(6.29,9.70) | 0.96(0.79,1.21) | 3.67(2.72,4.90) | 0.37(0.28,0.50) | -3.05(-6.72,0.76) |
| Nicaragua | 130.20(77.74,188.51) | 7.15(4.27,10.35) | 39.19(24.84,69.41) | 1.98(1.25,3.51) | -3.28(-5.66,-0.84) |
| Niger | 155.48(70.00,271.62) | 3.83(1.72,6.69) | 141.28(58.88,316.08) | 1.11(0.46,2.48) | -3.95(-9.00,1.38) |
| Nigeria | 20862.75(3529.40,36655.15) | 53.32(9.02,93.69) | 35231.25(6416.27,66200.91) | 34.68(6.32,65.17) | -1.21(-2.75,0.36) |
| Niue | 0.01(0.00,0.01) | 0.79(0.27,1.64) | 0.01(0.00,0.01) | 1.79(0.67,3.54) | 1.33(-3.88,6.82) |
| North Macedonia | 60.02(35.94,83.87) | 11.39(6.82,15.92) | 12.19(8.03,22.84) | 3.72(2.45,6.97) | -2.83(-4.66,-0.97) |
| Northern Mariana Islands | 0.02(0.01,0.04) | 0.14(0.06,0.34) | 0.02(0.01,0.04) | 0.21(0.09,0.38) | 2.54(-2.76,8.12) |
| Norway | 3.35(3.05,3.66) | 0.42(0.38,0.46) | 0.27(0.23,0.32) | 0.03(0.02,0.04) | -5.78(-11.58,0.39) |
| Oman | 72.98(30.15,126.72) | 8.68(3.59,15.08) | 32.10(13.56,55.58) | 2.62(1.11,4.54) | -2.06(-4.36,0.30) |
| Pakistan | 19026.18(9625.71,28733.48) | 38.64(19.55,58.35) | 30989.17(17028.68,48329.52) | 36.27(19.93,56.56) | 0.37(-0.98,1.73) |
| Palau | 0.00(0.00,0.00) | 0.00(0.00,0.00) | 0.00(0.00,0.00) | 0.00(0.00,0.00) | -1.17(-12.07,11.08) |
| Palestine | 109.74(65.86,163.69) | 11.33(6.80,16.91) | 106.64(61.73,161.23) | 5.71(3.31,8.63) | -1.23(-2.73,0.29) |
| Panama | 48.88(37.84,57.45) | 5.86(4.54,6.89) | 29.70(23.49,36.40) | 2.58(2.04,3.16) | -1.46(-4.47,1.65) |
| Papua New Guinea | 16.43(4.52,34.81) | 0.97(0.27,2.05) | 30.11(9.71,60.65) | 0.77(0.25,1.55) | -0.00(-4.61,4.82) |
| Paraguay | 83.81(47.58,133.22) | 5.02(2.85,7.98) | 59.14(34.37,100.72) | 2.95(1.71,5.02) | -1.24(-4.17,1.77) |
| Peru | 481.01(308.58,805.62) | 5.79(3.72,9.71) | 202.88(122.74,361.16) | 2.13(1.29,3.79) | -2.61(-6.06,0.96) |
| Philippines | 325.28(200.42,491.00) | 1.29(0.79,1.95) | 277.65(194.54,413.85) | 0.82(0.57,1.22) | -1.21(-5.83,3.63) |
| Poland | 731.62(687.75,776.65) | 7.64(7.18,8.11) | 52.52(46.71,59.32) | 0.89(0.79,1.01) | -5.06(-7.94,-2.10) |
| Portugal | 90.51(71.74,114.25) | 4.28(3.39,5.40) | 11.98(8.54,16.71) | 0.88(0.63,1.23) | -5.00(-8.21,-1.68) |
| Puerto Rico | 34.53(26.84,44.84) | 3.47(2.70,4.50) | 2.71(2.08,3.52) | 0.61(0.47,0.79) | -4.40(-7.87,-0.80) |
| Qatar | 2.83(1.51,5.53) | 2.26(1.21,4.43) | 4.79(2.31,9.59) | 0.97(0.47,1.94) | -1.56(-4.45,1.41) |
| Republic of Korea | 82.08(51.71,139.26) | 0.72(0.45,1.22) | 9.93(5.64,17.18) | 0.16(0.09,0.28) | -4.54(-9.61,0.83) |
| Republic of Moldova | 239.81(202.49,282.93) | 19.40(16.38,22.89) | 20.58(16.09,25.55) | 3.94(3.08,4.89) | -3.60(-5.66,-1.50) |
| Romania | 619.21(469.38,794.68) | 11.12(8.43,14.27) | 43.25(28.18,62.15) | 1.44(0.94,2.06) | -5.46(-8.14,-2.70) |
| Russian Federation | 3918.81(3716.90,4139.48) | 11.29(10.71,11.93) | 527.92(487.79,571.27) | 2.02(1.87,2.19) | -5.23(-7.34,-3.08) |
| Rwanda | 1393.11(811.33,2147.63) | 41.06(23.91,63.30) | 852.60(381.25,1613.52) | 17.15(7.67,32.46) | -3.45(-5.76,-1.08) |
| Saint Kitts and Nevis | 0.01(0.01,0.02) | 0.10(0.09,0.11) | 0.04(0.03,0.04) | 0.37(0.32,0.44) | 1.57(-5.26,8.91) |
| Saint Lucia | 2.20(1.81,2.80) | 4.26(3.51,5.44) | 0.78(0.60,0.97) | 2.62(2.02,3.26) | -1.79(-5.60,2.16) |
| Saint Vincent and the Grenadines | 1.97(1.61,2.40) | 4.80(3.91,5.85) | 0.56(0.45,0.69) | 2.26(1.82,2.76) | -1.66(-5.94,2.82) |
| Samoa | 6.32(3.08,11.64) | 8.87(4.33,16.33) | 4.31(1.93,8.90) | 5.40(2.42,11.13) | -1.39(-3.47,0.73) |
| San Marino | 0.22(0.12,0.36) | 5.27(2.98,8.83) | 0.08(0.05,0.16) | 1.90(1.05,3.60) | -3.10(-6.49,0.41) |
| Sao Tome and Principe | 0.05(0.03,0.09) | 0.09(0.05,0.16) | 0.03(0.01,0.07) | 0.04(0.02,0.08) | -3.09(-11.75,6.42) |
| Saudi Arabia | 375.96(81.12,809.47) | 5.74(1.24,12.35) | 118.17(21.14,260.26) | 1.56(0.28,3.44) | -3.93(-6.74,-1.04) |
| Senegal | 111.65(51.69,186.74) | 3.06(1.42,5.11) | 58.35(25.60,133.31) | 0.92(0.40,2.10) | -3.41(-8.36,1.81) |
| Serbia | 184.40(100.31,299.92) | 8.50(4.63,13.83) | 24.09(11.84,51.21) | 1.81(0.89,3.86) | -5.35(-7.51,-3.13) |
| Seychelles | 0.53(0.32,0.90) | 2.24(1.33,3.81) | 0.13(0.07,0.22) | 0.55(0.32,0.93) | -1.22(-4.73,2.41) |
| Sierra Leone | 51.09(21.10,87.83) | 2.82(1.16,4.85) | 40.03(18.48,81.24) | 1.12(0.52,2.27) | -2.66(-7.97,2.95) |
| Singapore | 7.46(5.92,9.45) | 1.15(0.91,1.46) | 2.08(1.50,2.72) | 0.26(0.19,0.33) | -2.52(-7.37,2.59) |
| Slovakia | 76.11(44.76,113.46) | 5.74(3.38,8.56) | 15.31(8.83,26.03) | 1.79(1.03,3.04) | -2.80(-5.46,-0.06) |
| Slovenia | 18.02(14.57,22.07) | 4.36(3.52,5.34) | 1.54(1.10,2.10) | 0.49(0.35,0.67) | -3.80(-7.04,-0.44) |
| Solomon Islands | 1.50(0.48,3.17) | 0.96(0.31,2.04) | 2.00(0.63,4.30) | 0.77(0.24,1.65) | 0.17(-3.77,4.27) |
| Somalia | 1074.33(571.34,1842.74) | 27.58(14.67,47.30) | 2163.42(943.78,4041.20) | 20.94(9.14,39.12) | -1.15(-3.11,0.85) |
| South Africa | 424.76(296.13,706.68) | 3.12(2.18,5.19) | 458.50(292.99,671.42) | 3.02(1.93,4.42) | -0.51(-3.25,2.32) |
| South Sudan | 657.51(353.94,1116.35) | 25.06(13.49,42.54) | 1047.00(606.78,1650.38) | 24.38(14.13,38.43) | 0.10(-2.43,2.70) |
| Spain | 327.86(264.20,415.57) | 4.18(3.37,5.30) | 63.31(46.40,86.62) | 0.98(0.72,1.34) | -4.06(-7.04,-0.98) |
| Sri Lanka | 222.91(136.16,333.94) | 4.03(2.46,6.04) | 55.74(30.48,105.39) | 1.09(0.60,2.06) | -3.84(-6.60,-1.01) |
| Sudan | 1008.06(500.45,1995.44) | 11.34(5.63,22.44) | 1145.29(618.46,1966.58) | 6.90(3.73,11.85) | -1.85(-4.31,0.66) |
| Suriname | 4.14(2.17,6.32) | 3.18(1.67,4.86) | 3.65(2.12,5.94) | 2.55(1.48,4.14) | -0.86(-4.59,3.02) |
| Sweden | 10.47(8.35,13.25) | 0.68(0.54,0.86) | 5.91(4.29,7.96) | 0.32(0.24,0.44) | -1.76(-5.62,2.25) |
| Switzerland | 31.38(25.49,37.83) | 2.72(2.21,3.27) | 4.93(3.72,6.46) | 0.37(0.28,0.49) | -4.94(-8.40,-1.35) |
| Syrian Arab Republic | 24.91(16.09,36.36) | 0.42(0.27,0.61) | 9.19(5.20,13.15) | 0.25(0.14,0.36) | -1.60(-7.33,4.48) |
| Taiwan (Province of China) | 62.55(51.07,75.44) | 1.14(0.93,1.37) | 6.76(5.26,8.52) | 0.23(0.18,0.29) | -3.88(-8.66,1.15) |
| Tajikistan | 56.44(26.84,96.48) | 2.43(1.16,4.16) | 62.48(34.79,116.45) | 1.74(0.97,3.25) | -2.05(-6.73,2.86) |
| Thailand | 452.09(250.94,682.66) | 2.68(1.49,4.05) | 69.14(34.28,145.62) | 0.71(0.35,1.49) | -4.10(-8.08,0.06) |
| Timor-Leste | 10.48(5.46,20.24) | 3.15(1.64,6.09) | 7.35(3.31,17.85) | 1.41(0.64,3.43) | -2.81(-6.39,0.90) |
| Togo | 45.65(21.90,76.54) | 2.59(1.24,4.34) | 28.27(12.89,58.35) | 0.85(0.39,1.76) | -2.72(-7.77,2.62) |
| Tokelau | 0.01(0.00,0.01) | 0.85(0.26,2.00) | 0.01(0.00,0.02) | 2.56(0.70,6.04) | 0.62(-4.13,5.61) |
| Tonga | 0.16(0.05,0.34) | 0.38(0.13,0.82) | 0.12(0.03,0.27) | 0.30(0.09,0.69) | -0.46(-7.16,6.72) |
| Trinidad and Tobago | 12.42(8.78,14.78) | 3.06(2.16,3.64) | 3.13(2.48,3.85) | 1.15(0.91,1.41) | -2.65(-6.82,1.71) |
| Tunisia | 685.88(351.19,1068.96) | 22.09(11.31,34.42) | 213.82(108.74,365.86) | 7.73(3.93,13.23) | -3.04(-4.76,-1.28) |
| Turkey | 1840.69(1102.35,3389.62) | 8.98(5.38,16.54) | 470.00(270.77,927.86) | 2.54(1.46,5.01) | -3.69(-6.37,-0.94) |
| Turkmenistan | 148.06(118.37,182.54) | 9.87(7.89,12.16) | 146.70(116.45,184.07) | 9.63(7.64,12.08) | -1.02(-1.78,-0.26) |
| Tuvalu | 0.04(0.01,0.09) | 1.22(0.36,2.54) | 0.02(0.01,0.05) | 0.61(0.20,1.24) | -1.64(-5.82,2.72) |
| Uganda | 2935.15(1575.44,4498.91) | 34.86(18.71,53.43) | 5970.06(2554.24,10457.60) | 30.10(12.88,52.72) | -0.37(-2.82,2.14) |
| Ukraine | 621.58(372.14,848.39) | 5.46(3.27,7.46) | 188.22(140.56,254.50) | 2.97(2.22,4.01) | -2.04(-4.37,0.34) |
| United Arab Emirates | 26.83(13.35,49.03) | 4.55(2.27,8.32) | 18.78(10.91,38.75) | 1.40(0.82,2.89) | -3.08(-5.34,-0.78) |
| United Kingdom | 222.89(213.04,234.90) | 2.04(1.95,2.15) | 82.50(76.45,89.42) | 0.70(0.65,0.76) | -2.68(-5.84,0.58) |
| United Republic of Tanzania | 3638.20(2118.30,5515.38) | 30.13(17.54,45.67) | 4507.53(2234.88,8025.58) | 18.47(9.16,32.89) | -1.04(-3.49,1.47) |
| United States Virgin Islands | 0.24(0.14,0.45) | 0.76(0.43,1.42) | 0.04(0.01,0.08) | 0.27(0.09,0.60) | -2.87(-8.18,2.75) |
| United States of America | 965.88(932.17,1003.85) | 1.73(1.67,1.80) | 288.22(269.18,311.71) | 0.48(0.45,0.52) | -3.65(-6.76,-0.43) |
| Uruguay | 37.35(29.19,46.22) | 4.56(3.57,5.65) | 11.90(9.24,15.60) | 1.80(1.40,2.37) | -2.74(-5.90,0.52) |
| Uzbekistan | 913.04(622.10,1271.61) | 10.67(7.27,14.86) | 600.22(419.41,843.83) | 5.95(4.16,8.36) | -1.39(-3.63,0.90) |
| Vanuatu | 0.50(0.16,1.02) | 0.73(0.24,1.50) | 0.77(0.26,1.56) | 0.66(0.22,1.34) | 0.50(-3.51,4.68) |
| Venezuela (Bolivarian Republic of) | 522.76(439.23,601.12) | 7.37(6.19,8.47) | 337.37(255.74,432.19) | 5.09(3.86,6.52) | -1.01(-3.52,1.56) |
| Viet Nam | 1142.74(432.05,2025.07) | 4.31(1.63,7.64) | 590.15(254.05,1062.04) | 2.38(1.03,4.29) | -1.53(-4.52,1.56) |
| Yemen | 578.89(326.21,1044.66) | 8.16(4.60,14.73) | 874.42(477.68,1570.06) | 6.34(3.46,11.39) | -1.40(-3.22,0.45) |
| Zambia | 1063.20(598.79,1696.32) | 28.32(15.95,45.18) | 1339.44(619.02,2518.99) | 16.19(7.48,30.45) | -1.95(-4.39,0.57) |
| Zimbabwe | 237.44(93.33,387.72) | 4.93(1.94,8.05) | 515.80(251.39,831.55) | 8.20(3.99,13.21) | 4.17(1.09,7.34) |
| ^a^EAPC is expressed as 95% CIs | | | | | |

**Table S6** DALYs of Non-Hodgkin lymphoma in Children Between 1990 and 2021 at 204 Countries and Territories.

| Location | Rate per 100000 (95% UI) | | | | |
| --- | --- | --- | --- | --- | --- |
|  | 1990 | | 2021 | | EAPC^a^ |
|  | Cases | Rates | Cases | Rates |  |
| Global | 1102346.32(836303.95,1316917.95) | 63.38(48.09,75.72) | 761085.36(602606.50,948912.73) | 37.83(29.95,47.17) | -1.51(-1.61,-1.40) |
| 5 SDI | | | | | |
| High SDI | 42938.50(40791.48,45092.95) | 23.11(21.95,24.27) | 16201.97(14995.59,17393.34) | 9.39(8.69,10.08) | -2.96(-3.05,-2.87) |
| High-middle SDI | 138345.40(119944.22,158628.66) | 50.56(43.84,57.97) | 37408.44(32186.93,44297.50) | 16.20(13.94,19.19) | -3.65(-3.84,-3.47) |
| Middle SDI | 303674.44(255665.33,353037.50) | 52.61(44.29,61.16) | 125629.47(103248.82,155971.70) | 22.16(18.21,27.51) | -2.60(-2.78,-2.43) |
| Low-middle SDI | 306898.12(205627.98,391484.01) | 65.01(43.55,82.92) | 230248.63(185145.73,293375.42) | 39.71(31.93,50.60) | -1.44(-1.53,-1.35) |
| Low SDI | 309591.61(201695.81,409472.51) | 135.24(88.11,178.88) | 350984.47(240530.35,471925.94) | 76.26(52.26,102.54) | -1.71(-1.81,-1.60) |
| 21 GBD Region | | | | | |
| Andean Latin America | 10031.91(8381.31,12474.55) | 67.55(56.43,83.99) | 5857.70(4682.62,7553.76) | 32.37(25.88,41.75) | -2.22(-2.40,-2.03) |
| Australasia | 972.43(862.91,1094.07) | 21.20(18.82,23.86) | 409.97(336.55,484.22) | 7.15(5.87,8.45) | -3.43(-3.99,-2.86) |
| Caribbean | 12052.53(7142.76,16393.66) | 105.61(62.59,143.65) | 7730.79(4566.73,11149.79) | 67.19(39.69,96.91) | -1.08(-1.35,-0.81) |
| Central Asia | 13504.65(11053.23,15688.07) | 54.04(44.23,62.77) | 8243.72(6808.41,10244.58) | 29.79(24.60,37.02) | -2.01(-2.20,-1.83) |
| Central Europe | 10612.93(9989.53,11244.68) | 36.00(33.88,38.14) | 2578.10(2333.22,2855.72) | 14.56(13.18,16.13) | -2.79(-3.06,-2.52) |
| Central Latin America | 28353.04(26468.32,30385.17) | 44.04(41.11,47.20) | 12411.28(10568.73,14725.47) | 19.55(16.65,23.20) | -2.09(-2.30,-1.89) |
| Central Sub-Saharan Africa | 17560.51(5257.12,25953.29) | 69.41(20.78,102.59) | 16677.96(11189.67,23091.16) | 28.42(19.07,39.35) | -2.51(-2.72,-2.30) |
| East Asia | 209159.50(173974.04,255126.65) | 63.41(52.75,77.35) | 42491.44(34061.03,54539.66) | 15.89(12.74,20.40) | -4.48(-4.83,-4.12) |
| Eastern Europe | 25740.17(24460.27,26923.32) | 50.02(47.53,52.32) | 5748.47(5342.40,6223.71) | 16.22(15.07,17.56) | -3.70(-4.13,-3.27) |
| Eastern Sub-Saharan Africa | 168477.30(119364.46,226748.53) | 186.02(131.79,250.36) | 176326.64(118574.95,250699.21) | 98.82(66.45,140.50) | -1.84(-1.97,-1.71) |
| High-income Asia Pacific | 9952.21(9087.41,11209.30) | 28.27(25.82,31.85) | 2527.38(2347.43,2728.34) | 11.27(10.47,12.17) | -3.13(-3.35,-2.90) |
| High-income North America | 10433.52(10115.12,10806.48) | 16.92(16.40,17.52) | 4243.12(3922.81,4581.94) | 6.47(5.98,6.98) | -3.24(-3.41,-3.08) |
| North Africa and Middle East | 69726.00(51695.85,93026.52) | 49.63(36.80,66.22) | 39550.74(32870.68,51394.60) | 21.57(17.93,28.04) | -2.41(-2.61,-2.21) |
| Oceania | 589.08(378.09,854.00) | 21.98(14.11,31.87) | 1318.87(870.08,1963.71) | 25.96(17.12,38.65) | 0.73(0.50,0.96) |
| South Asia | 268297.54(167728.63,350213.37) | 61.91(38.70,80.81) | 178714.33(140209.04,233215.82) | 35.25(27.65,46.00) | -1.74(-1.86,-1.61) |
| Southeast Asia | 73783.18(46430.36,98374.35) | 43.21(27.19,57.61) | 39411.06(31914.86,53265.23) | 22.83(18.48,30.85) | -1.95(-2.01,-1.88) |
| Southern Latin America | 5080.94(4633.35,5597.39) | 34.04(31.04,37.50) | 2452.92(2085.22,2873.45) | 16.92(14.39,19.82) | -1.93(-2.23,-1.62) |
| Southern Sub-Saharan Africa | 6319.04(4995.97,7927.51) | 30.54(24.15,38.32) | 10827.95(7669.36,13967.35) | 44.99(31.87,58.04) | 1.91(1.43,2.40) |
| Tropical Latin America | 24912.55(22230.82,27647.58) | 46.47(41.46,51.57) | 8500.52(6874.04,10030.48) | 16.94(13.70,19.98) | -2.70(-3.15,-2.24) |
| Western Europe | 15928.12(15288.53,16594.16) | 22.43(21.53,23.37) | 7114.41(6543.20,7744.55) | 10.44(9.61,11.37) | -2.60(-2.76,-2.43) |
| Western Sub-Saharan Africa | 120859.18(84772.51,160450.53) | 137.53(96.46,182.58) | 187947.98(119464.74,250299.69) | 87.51(55.63,116.55) | -1.27(-1.40,-1.14) |
| 204 countries and territories | | | | | |
| Afghanistan | 2472.06(864.07,4143.00) | 57.38(20.06,96.17) | 4246.14(2684.38,6970.21) | 29.90(18.90,49.08) | -1.76(-2.03,-1.48) |
| Albania | 358.32(259.55,468.40) | 32.07(23.23,41.92) | 69.87(46.19,98.04) | 15.75(10.41,22.10) | -1.98(-2.71,-1.24) |
| Algeria | 6750.50(4855.81,8774.61) | 62.94(45.28,81.82) | 3683.90(2684.48,4994.12) | 27.69(20.18,37.54) | -2.43(-2.56,-2.30) |
| American Samoa | 2.36(1.67,3.40) | 12.41(8.76,17.89) | 2.89(1.81,4.53) | 20.38(12.78,31.95) | 2.18(1.92,2.44) |
| Andorra | 5.49(3.50,7.74) | 57.76(36.86,81.49) | 1.56(1.10,2.18) | 15.34(10.78,21.40) | -3.93(-4.18,-3.68) |
| Angola | 4703.44(995.13,7831.36) | 99.76(21.11,166.10) | 5021.65(3071.08,7472.91) | 32.94(20.14,49.02) | -3.37(-3.69,-3.05) |
| Antigua and Barbuda | 5.86(4.87,6.92) | 32.19(26.76,38.07) | 4.53(3.99,5.20) | 26.79(23.58,30.75) | -0.53(-1.04,-0.01) |
| Argentina | 3644.19(3208.26,4144.74) | 35.96(31.65,40.89) | 1915.06(1572.28,2302.72) | 18.81(15.44,22.61) | -1.72(-2.02,-1.41) |
| Armenia | 262.50(188.53,319.41) | 25.16(18.07,30.62) | 114.97(91.47,147.01) | 19.41(15.44,24.82) | 0.74(0.13,1.36) |
| Australia | 848.31(747.27,966.56) | 22.41(19.74,25.53) | 327.52(262.80,392.74) | 6.90(5.53,8.27) | -3.59(-4.09,-3.08) |
| Austria | 247.62(226.11,270.40) | 18.36(16.77,20.05) | 115.16(100.53,132.74) | 8.88(7.75,10.23) | -1.98(-2.25,-1.71) |
| Azerbaijan | 961.78(656.68,1340.44) | 39.63(27.06,55.23) | 663.48(428.70,1033.46) | 28.11(18.16,43.78) | -1.60(-1.94,-1.27) |
| Bahamas | 36.20(30.78,42.15) | 44.88(38.16,52.25) | 22.51(17.95,28.30) | 27.73(22.11,34.86) | -1.81(-2.23,-1.40) |
| Bahrain | 40.96(30.80,52.60) | 25.09(18.87,32.22) | 36.66(26.62,49.85) | 12.36(8.97,16.80) | -2.33(-2.72,-1.95) |
| Bangladesh | 47093.96(20169.39,70118.11) | 96.28(41.24,143.36) | 17444.32(11465.90,28988.02) | 38.12(25.05,63.34) | -2.77(-2.91,-2.63) |
| Barbados | 47.64(41.46,54.61) | 76.39(66.49,87.57) | 17.75(13.06,23.36) | 37.70(27.73,49.60) | -1.15(-1.65,-0.65) |
| Belarus | 346.06(288.60,420.66) | 14.40(12.01,17.50) | 322.85(258.21,416.33) | 20.46(16.36,26.38) | 2.39(1.71,3.07) |
| Belgium | 351.64(304.34,402.51) | 19.47(16.85,22.29) | 253.43(203.91,314.59) | 13.25(10.66,16.45) | -1.98(-2.25,-1.72) |
| Belize | 36.66(32.41,40.80) | 44.78(39.59,49.84) | 18.92(16.06,22.34) | 15.36(13.04,18.15) | -2.76(-3.06,-2.45) |
| Benin | 2749.21(1791.93,4022.79) | 113.52(73.99,166.11) | 4997.59(3141.59,7172.44) | 82.19(51.67,117.96) | -0.80(-0.95,-0.66) |
| Bermuda | 5.91(4.89,7.16) | 49.56(40.98,60.07) | 2.24(1.78,2.74) | 26.59(21.14,32.43) | -1.58(-1.96,-1.20) |
| Bhutan | 198.03(88.87,315.79) | 75.53(33.90,120.44) | 69.41(40.10,131.05) | 37.08(21.42,70.02) | -2.73(-3.04,-2.42) |
| Bolivia (Plurinational State of) | 2857.42(2014.14,3851.02) | 106.39(74.99,143.38) | 1588.99(1167.02,2141.13) | 45.58(33.47,61.41) | -2.82(-2.93,-2.71) |
| Bosnia and Herzegovina | 235.81(182.51,297.22) | 21.52(16.66,27.13) | 68.66(45.88,92.28) | 14.00(9.36,18.82) | -1.08(-1.39,-0.77) |
| Botswana | 147.65(98.48,221.23) | 25.01(16.68,37.47) | 256.17(164.63,380.38) | 36.69(23.58,54.47) | 2.00(1.60,2.41) |
| Brazil | 24227.17(21559.59,26907.74) | 46.64(41.51,51.80) | 7954.86(6438.17,9378.74) | 16.51(13.36,19.46) | -2.78(-3.25,-2.30) |
| Brunei Darussalam | 44.46(32.67,59.44) | 49.09(36.07,65.62) | 21.56(16.09,27.35) | 22.79(17.01,28.91) | -1.34(-1.83,-0.85) |
| Bulgaria | 590.62(521.17,650.62) | 34.02(30.02,37.47) | 182.95(157.72,213.21) | 18.74(16.16,21.84) | -1.90(-2.36,-1.43) |
| Burkina Faso | 5301.70(3430.75,7634.18) | 112.35(72.70,161.77) | 8898.03(6146.28,12335.88) | 85.79(59.26,118.93) | -0.51(-0.71,-0.31) |
| Burundi | 5781.21(3564.11,8940.42) | 220.56(135.98,341.09) | 4582.71(2489.41,8465.05) | 78.28(42.52,144.60) | -2.80(-3.04,-2.55) |
| Cabo Verde | 25.82(14.98,43.09) | 16.41(9.52,27.39) | 81.86(34.47,115.40) | 57.16(24.07,80.59) | 2.74(1.77,3.71) |
| Cambodia | 2426.64(1012.02,3600.11) | 52.06(21.71,77.24) | 1302.09(911.00,2090.62) | 25.45(17.80,40.86) | -2.66(-2.91,-2.41) |
| Cameroon | 5222.59(3455.27,7200.70) | 106.98(70.78,147.49) | 12028.32(8023.55,16699.25) | 89.32(59.58,124.00) | -0.19(-0.40,0.02) |
| Canada | 1284.12(1129.46,1449.59) | 22.33(19.64,25.20) | 385.34(316.03,466.71) | 6.24(5.12,7.56) | -4.86(-5.29,-4.43) |
| Central African Republic | 910.99(257.89,1513.29) | 74.51(21.09,123.77) | 1139.34(568.37,1785.06) | 49.89(24.89,78.16) | -1.02(-1.18,-0.87) |
| Chad | 2675.95(1636.23,4050.82) | 91.44(55.91,138.42) | 7606.41(4744.22,10985.02) | 84.38(52.63,121.85) | 0.10(-0.04,0.23) |
| Chile | 1150.74(1023.25,1292.83) | 28.97(25.76,32.55) | 407.24(343.22,485.22) | 11.15(9.40,13.29) | -2.90(-3.21,-2.59) |
| China | 205190.83(170360.96,250898.13) | 64.45(53.51,78.81) | 41207.39(32904.56,52922.11) | 15.87(12.67,20.38) | -4.54(-4.90,-4.17) |
| Colombia | 6574.80(5652.43,7516.53) | 56.37(48.46,64.45) | 2519.46(1997.88,3159.98) | 23.74(18.82,29.77) | -1.83(-2.22,-1.44) |
| Comoros | 289.97(154.96,417.57) | 136.33(72.86,196.33) | 228.93(150.02,344.38) | 95.33(62.47,143.40) | -1.25(-1.62,-0.88) |
| Congo | 575.55(243.29,881.13) | 54.66(23.11,83.68) | 549.66(388.71,744.20) | 28.49(20.15,38.57) | -2.03(-2.25,-1.81) |
| Cook Islands | 0.69(0.45,0.98) | 10.50(6.75,14.80) | 0.44(0.23,0.84) | 11.51(6.10,22.07) | -1.27(-1.88,-0.66) |
| Costa Rica | 432.61(380.23,501.13) | 38.48(33.82,44.58) | 271.14(218.81,340.29) | 26.65(21.51,33.45) | -1.19(-1.37,-1.01) |
| Croatia | 295.36(243.32,352.74) | 29.92(24.65,35.74) | 101.18(76.53,130.10) | 16.94(12.82,21.79) | -1.28(-1.86,-0.70) |
| Cuba | 1458.87(1303.12,1633.56) | 58.26(52.04,65.24) | 465.15(372.92,570.19) | 26.18(20.99,32.09) | -2.04(-2.39,-1.68) |
| Cyprus | 45.36(33.38,58.32) | 22.92(16.86,29.46) | 19.27(13.90,26.11) | 8.81(6.35,11.94) | -2.37(-3.27,-1.45) |
| Czechia | 619.90(528.24,724.05) | 28.13(23.97,32.86) | 260.66(199.33,328.57) | 15.19(11.61,19.14) | -1.55(-1.89,-1.21) |
| Côte d'Ivoire | 6415.92(4529.66,8901.27) | 112.49(79.42,156.07) | 10995.49(7375.21,18253.39) | 95.02(63.73,157.74) | -0.20(-0.43,0.03) |
| Democratic People's Republic of Korea | 2124.14(1345.88,3095.05) | 35.70(22.62,52.02) | 829.87(543.64,1313.31) | 17.38(11.39,27.51) | -2.03(-2.29,-1.77) |
| Democratic Republic of the Congo | 11068.64(3635.49,16225.08) | 62.52(20.54,91.65) | 9585.11(6096.10,13840.43) | 25.22(16.04,36.42) | -2.44(-2.67,-2.22) |
| Denmark | 167.55(145.95,193.98) | 18.97(16.52,21.96) | 76.36(61.78,94.16) | 8.00(6.47,9.87) | -3.18(-3.43,-2.92) |
| Djibouti | 215.94(142.05,311.47) | 124.02(81.58,178.89) | 320.57(190.79,537.97) | 77.59(46.18,130.21) | -1.41(-1.80,-1.01) |
| Dominica | 15.93(12.39,20.34) | 64.19(49.94,81.98) | 13.87(10.06,18.80) | 101.35(73.50,137.37) | 1.80(1.55,2.05) |
| Dominican Republic | 1655.76(1206.33,2088.65) | 61.43(44.75,77.49) | 810.84(539.44,1164.29) | 27.59(18.36,39.62) | -2.70(-3.01,-2.39) |
| Ecuador | 1508.31(1338.42,1717.51) | 39.02(34.63,44.43) | 1231.54(985.68,1533.06) | 24.29(19.44,30.23) | -1.42(-2.00,-0.85) |
| Egypt | 8055.13(6033.22,13575.16) | 36.31(27.20,61.20) | 3580.80(2221.73,8029.06) | 9.72(6.03,21.79) | -3.97(-4.56,-3.37) |
| El Salvador | 694.17(546.75,871.82) | 32.17(25.34,40.40) | 296.22(226.71,384.92) | 16.29(12.46,21.16) | -1.95(-2.19,-1.71) |
| Equatorial Guinea | 129.49(43.24,203.71) | 65.76(21.96,103.45) | 171.88(84.53,328.90) | 29.38(14.45,56.22) | -3.29(-3.63,-2.94) |
| Eritrea | 2437.88(1597.04,3357.97) | 153.14(100.32,210.94) | 2428.78(1453.44,4165.78) | 96.20(57.57,165.01) | -1.43(-1.60,-1.26) |
| Estonia | 103.71(83.94,127.53) | 29.71(24.04,36.53) | 72.01(54.94,94.60) | 33.32(25.42,43.77) | 0.15(-0.65,0.96) |
| Eswatini | 154.77(88.23,238.62) | 40.12(22.87,61.86) | 178.92(113.31,277.81) | 43.36(27.46,67.33) | 0.61(0.32,0.90) |
| Ethiopia | 52822.17(13975.63,82042.95) | 216.81(57.36,336.74) | 37694.23(26300.20,52216.50) | 84.99(59.30,117.74) | -3.35(-3.63,-3.07) |
| Fiji | 35.26(24.19,54.22) | 12.53(8.60,19.26) | 62.52(40.12,93.38) | 22.94(14.72,34.26) | 2.28(2.03,2.53) |
| Finland | 197.03(164.90,232.92) | 20.42(17.09,24.14) | 110.13(85.82,137.95) | 13.00(10.13,16.29) | -2.19(-2.61,-1.77) |
| France | 2504.11(2229.07,2777.52) | 21.38(19.03,23.71) | 1373.76(1156.73,1635.58) | 11.83(9.97,14.09) | -2.29(-2.50,-2.08) |
| Gabon | 172.41(103.98,247.98) | 42.31(25.52,60.85) | 210.33(135.26,312.39) | 32.91(21.16,48.88) | -0.23(-0.46,0.00) |
| Gambia | 564.52(377.82,780.77) | 122.38(81.91,169.27) | 742.73(483.60,1254.18) | 74.76(48.68,126.25) | -1.75(-2.04,-1.46) |
| Georgia | 803.69(577.28,1051.92) | 58.72(42.18,76.85) | 111.48(78.98,149.40) | 15.15(10.73,20.30) | -4.09(-4.80,-3.38) |
| Germany | 2454.28(2195.26,2757.54) | 18.96(16.96,21.30) | 1138.30(980.33,1335.45) | 9.51(8.19,11.16) | -2.04(-2.27,-1.80) |
| Ghana | 10676.87(5011.37,16303.32) | 158.96(74.61,242.73) | 5146.53(3332.52,8602.00) | 39.95(25.87,66.77) | -5.74(-6.68,-4.80) |
| Greece | 185.84(167.80,204.23) | 9.18(8.29,10.09) | 77.73(66.92,89.72) | 5.57(4.80,6.43) | -1.28(-1.63,-0.93) |
| Greenland | 7.23(3.31,10.33) | 50.83(23.28,72.60) | 1.68(1.11,2.40) | 14.29(9.47,20.39) | -3.84(-4.35,-3.33) |
| Grenada | 40.74(32.71,50.50) | 121.96(97.92,151.16) | 17.83(14.43,21.96) | 81.68(66.09,100.60) | -0.69(-1.01,-0.38) |
| Guam | 9.91(8.09,12.25) | 23.76(19.39,29.37) | 6.52(4.66,8.93) | 17.82(12.75,24.42) | 1.64(0.73,2.56) |
| Guatemala | 1416.16(1284.24,1565.40) | 34.87(31.62,38.55) | 655.70(538.72,813.70) | 13.29(10.92,16.49) | -2.21(-2.52,-1.91) |
| Guinea | 4154.44(2770.17,5784.85) | 150.97(100.67,210.22) | 4219.68(2606.69,8401.62) | 69.80(43.12,138.97) | -1.91(-2.09,-1.74) |
| Guinea-Bissau | 692.32(431.94,1026.39) | 143.52(89.54,212.77) | 578.15(357.19,908.82) | 64.37(39.77,101.19) | -2.31(-2.65,-1.96) |
| Guyana | 120.43(96.65,144.81) | 40.97(32.88,49.27) | 67.43(51.53,86.97) | 31.60(24.15,40.76) | 0.75(0.23,1.27) |
| Haiti | 6531.48(1780.38,10536.87) | 240.74(65.62,388.37) | 5527.03(2347.52,8912.17) | 126.98(53.93,204.75) | -1.60(-1.88,-1.32) |
| Honduras | 575.91(436.71,743.05) | 26.07(19.77,33.63) | 268.74(143.32,455.47) | 8.20(4.37,13.90) | -3.87(-4.13,-3.62) |
| Hungary | 656.37(552.85,775.63) | 30.80(25.94,36.40) | 189.48(155.11,235.24) | 13.65(11.17,16.94) | -1.79(-2.21,-1.38) |
| Iceland | 13.41(11.26,15.55) | 21.14(17.74,24.51) | 8.49(6.41,10.95) | 12.58(9.49,16.21) | -1.20(-1.92,-0.48) |
| India | 166972.41(99052.82,224263.48) | 51.14(30.34,68.68) | 67096.50(52313.80,86213.80) | 18.31(14.28,23.53) | -3.32(-3.55,-3.09) |
| Indonesia | 26032.64(14063.48,39380.34) | 38.43(20.76,58.14) | 13940.58(9909.76,21671.44) | 20.72(14.73,32.21) | -1.86(-1.91,-1.81) |
| Iran (Islamic Republic of) | 7862.73(6115.66,10181.00) | 30.97(24.09,40.11) | 3442.06(2687.25,4522.35) | 17.06(13.32,22.41) | -1.24(-1.77,-0.70) |
| Iraq | 5749.50(3367.06,8154.29) | 69.81(40.88,99.00) | 3474.17(2408.15,4878.76) | 25.81(17.89,36.24) | -2.74(-3.08,-2.39) |
| Ireland | 207.87(174.24,242.67) | 21.16(17.73,24.70) | 85.33(66.53,107.67) | 8.56(6.67,10.80) | -2.82(-3.21,-2.43) |
| Israel | 484.33(429.68,547.62) | 31.59(28.03,35.72) | 372.59(307.78,455.50) | 14.18(11.71,17.33) | -2.38(-2.80,-1.97) |
| Italy | 2960.86(2773.64,3152.95) | 32.08(30.05,34.16) | 1000.16(882.12,1146.00) | 13.16(11.61,15.08) | -3.34(-3.59,-3.09) |
| Jamaica | 781.59(656.93,908.44) | 93.58(78.65,108.77) | 254.82(199.79,322.32) | 43.64(34.21,55.20) | -2.36(-2.70,-2.01) |
| Japan | 5767.83(5597.63,5916.43) | 24.98(24.24,25.62) | 1865.83(1754.76,1967.13) | 12.08(11.36,12.74) | -2.50(-2.73,-2.27) |
| Jordan | 888.52(659.37,1199.41) | 54.40(40.37,73.43) | 1331.43(985.99,1800.83) | 36.65(27.14,49.57) | -1.60(-1.98,-1.22) |
| Kazakhstan | 2966.93(2592.46,3355.41) | 57.10(49.89,64.57) | 814.86(658.68,1002.07) | 15.02(12.14,18.47) | -4.33(-4.78,-3.89) |
| Kenya | 5422.47(3565.74,7784.03) | 48.54(31.92,69.69) | 5936.81(4454.86,7555.36) | 31.81(23.87,40.48) | -0.21(-0.72,0.31) |
| Kiribati | 2.91(1.65,4.06) | 9.86(5.57,13.76) | 3.06(1.49,5.14) | 7.28(3.53,12.23) | -1.07(-1.26,-0.87) |
| Kuwait | 350.08(301.13,410.07) | 63.15(54.32,73.97) | 172.39(132.34,213.75) | 20.39(15.65,25.28) | -1.73(-2.67,-0.78) |
| Kyrgyzstan | 513.22(404.43,673.66) | 30.59(24.11,40.16) | 335.06(253.97,426.31) | 14.73(11.17,18.74) | -2.29(-2.81,-1.76) |
| Lao People's Democratic Republic | 1154.45(391.52,1820.40) | 62.64(21.24,98.77) | 645.70(415.24,1030.18) | 28.12(18.08,44.86) | -2.65(-2.72,-2.59) |
| Latvia | 99.43(85.02,114.73) | 17.48(14.94,20.17) | 41.19(33.42,50.42) | 13.87(11.25,16.97) | 0.99(0.26,1.73) |
| Lebanon | 541.64(374.37,732.47) | 51.79(35.79,70.03) | 316.08(221.84,445.30) | 24.73(17.36,34.84) | -2.49(-2.83,-2.15) |
| Lesotho | 152.27(101.73,230.65) | 22.31(14.90,33.79) | 248.15(157.42,359.68) | 39.36(24.97,57.05) | 2.84(2.46,3.22) |
| Liberia | 1779.84(992.79,2457.02) | 157.48(87.84,217.40) | 1579.24(1032.43,2242.50) | 72.25(47.23,102.59) | -2.37(-3.08,-1.66) |
| Libya | 1134.29(761.33,1503.12) | 62.64(42.04,83.00) | 1081.52(755.02,1480.62) | 72.51(50.62,99.26) | 0.99(0.69,1.29) |
| Lithuania | 126.29(103.16,152.69) | 15.20(12.42,18.38) | 80.86(63.66,103.90) | 19.83(15.61,25.48) | 0.50(-0.11,1.11) |
| Luxembourg | 13.43(12.03,15.02) | 20.33(18.20,22.73) | 9.47(7.32,11.63) | 9.36(7.23,11.49) | -3.91(-4.41,-3.40) |
| Madagascar | 7696.33(5491.06,10148.43) | 141.06(100.64,186.01) | 8173.41(5533.49,11700.36) | 69.66(47.16,99.72) | -1.81(-2.02,-1.60) |
| Malawi | 24424.20(17655.44,32189.06) | 536.84(388.06,707.51) | 22003.69(10995.54,46258.79) | 270.85(135.35,569.42) | -1.85(-2.13,-1.57) |
| Malaysia | 1879.71(1321.95,2501.76) | 28.60(20.11,38.06) | 1157.82(809.61,1537.51) | 15.21(10.63,20.19) | -1.60(-1.94,-1.26) |
| Maldives | 38.74(17.96,56.72) | 36.89(17.10,53.99) | 13.06(9.09,19.25) | 13.03(9.08,19.22) | -2.94(-3.14,-2.73) |
| Mali | 4047.14(2673.62,5933.12) | 98.00(64.74,143.67) | 5065.90(3025.94,9389.20) | 43.76(26.14,81.11) | -2.27(-2.45,-2.09) |
| Malta | 16.89(12.00,20.25) | 19.30(13.71,23.14) | 21.15(15.39,29.20) | 33.04(24.05,45.61) | -0.30(-1.09,0.50) |
| Marshall Islands | 2.54(1.69,3.84) | 11.57(7.72,17.48) | 3.52(2.22,5.56) | 20.19(12.71,31.87) | 1.71(1.25,2.16) |
| Mauritania | 626.48(419.31,890.52) | 67.77(45.36,96.34) | 856.72(557.23,1203.04) | 46.23(30.07,64.92) | -1.39(-1.68,-1.11) |
| Mauritius | 22.12(20.22,24.16) | 6.70(6.13,7.32) | 23.65(20.71,26.65) | 11.40(9.98,12.85) | 0.54(-0.09,1.18) |
| Mexico | 14495.09(13181.16,16051.54) | 43.38(39.45,48.04) | 5564.45(4752.36,6515.45) | 17.35(14.82,20.32) | -2.44(-2.76,-2.13) |
| Micronesia (Federated States of) | 8.92(5.57,13.38) | 19.42(12.13,29.13) | 5.39(3.47,8.33) | 17.61(11.32,27.21) | -0.20(-0.36,-0.03) |
| Monaco | 0.75(0.50,1.12) | 21.27(14.11,31.69) | 0.91(0.66,1.29) | 18.33(13.24,25.89) | -1.83(-2.35,-1.31) |
| Mongolia | 757.68(490.31,1057.93) | 84.19(54.48,117.55) | 231.06(161.26,324.86) | 21.26(14.84,29.90) | -4.61(-5.13,-4.10) |
| Montenegro | 52.22(40.75,63.70) | 32.32(25.22,39.42) | 11.78(8.58,17.45) | 10.57(7.70,15.66) | -3.27(-3.88,-2.66) |
| Morocco | 4631.99(2952.67,6525.31) | 47.33(30.17,66.68) | 1944.13(1328.18,2864.73) | 19.85(13.56,29.26) | -2.37(-2.59,-2.15) |
| Mozambique | 1858.35(1013.16,4605.30) | 29.95(16.33,74.23) | 1728.77(846.79,4160.29) | 12.12(5.94,29.16) | -2.45(-2.76,-2.14) |
| Myanmar | 10669.60(3643.74,16736.69) | 72.21(24.66,113.27) | 4977.54(3219.87,7727.28) | 31.88(20.62,49.49) | -2.88(-3.03,-2.73) |
| Namibia | 277.09(175.68,400.43) | 46.12(29.24,66.66) | 471.06(284.40,717.00) | 57.07(34.45,86.86) | 1.69(1.10,2.30) |
| Nauru | 1.13(0.76,1.59) | 26.78(18.05,37.63) | 1.36(0.84,2.01) | 34.12(21.14,50.58) | 0.70(0.22,1.18) |
| Nepal | 6187.69(2774.03,9364.96) | 73.44(32.93,111.15) | 2448.25(1550.67,4202.57) | 26.53(16.81,45.55) | -2.95(-3.19,-2.71) |
| Netherlands | 597.97(525.11,676.54) | 21.94(19.27,24.82) | 281.60(231.89,331.63) | 10.50(8.65,12.36) | -2.36(-2.63,-2.09) |
| New Zealand | 124.11(107.58,141.76) | 15.51(13.45,17.72) | 82.45(69.94,100.10) | 8.40(7.12,10.20) | -2.57(-3.58,-1.55) |
| Nicaragua | 789.42(595.74,1017.35) | 43.35(32.71,55.86) | 244.53(183.47,326.59) | 12.35(9.26,16.49) | -3.14(-3.43,-2.86) |
| Niger | 6575.93(3684.75,9659.84) | 161.85(90.69,237.75) | 7587.74(4450.54,11945.73) | 59.45(34.87,93.59) | -3.25(-3.50,-2.99) |
| Nigeria | 61011.32(38081.15,85319.48) | 155.94(97.33,218.07) | 109622.16(61684.54,157384.74) | 107.91(60.72,154.92) | -0.92(-1.05,-0.79) |
| Niue | 0.16(0.11,0.23) | 19.71(13.20,28.50) | 0.53(0.36,0.79) | 136.33(92.68,203.11) | 2.69(1.40,3.99) |
| North Macedonia | 189.38(154.06,237.58) | 35.95(29.25,45.10) | 49.43(34.79,65.67) | 15.09(10.62,20.05) | -1.99(-2.55,-1.43) |
| Northern Mariana Islands | 0.73(0.44,1.43) | 6.02(3.63,11.74) | 1.10(0.72,1.70) | 9.75(6.39,15.12) | 2.75(1.96,3.54) |
| Norway | 209.51(193.76,227.44) | 26.24(24.27,28.49) | 64.26(55.83,74.38) | 6.96(6.04,8.05) | -3.84(-4.29,-3.39) |
| Oman | 306.09(197.74,440.73) | 36.42(23.53,52.44) | 224.76(164.18,289.22) | 18.38(13.42,23.65) | -1.05(-1.50,-0.60) |
| Pakistan | 47845.44(34279.68,63555.81) | 97.16(69.61,129.07) | 91655.86(62965.53,130514.42) | 107.27(73.69,152.75) | 0.78(0.60,0.96) |
| Palau | 0.45(0.30,0.62) | 9.86(6.63,13.60) | 0.23(0.16,0.36) | 7.18(4.86,11.13) | -0.84(-1.03,-0.66) |
| Palestine | 282.10(198.15,386.94) | 29.13(20.46,39.96) | 323.41(241.30,435.21) | 17.32(12.92,23.31) | -0.94(-1.38,-0.50) |
| Panama | 334.57(288.46,388.71) | 40.12(34.59,46.61) | 376.24(307.95,452.47) | 32.62(26.70,39.23) | -0.62(-0.82,-0.42) |
| Papua New Guinea | 410.05(228.26,642.58) | 24.12(13.43,37.80) | 1070.85(646.55,1672.96) | 27.34(16.51,42.71) | 0.58(0.33,0.83) |
| Paraguay | 685.38(516.09,894.41) | 41.05(30.91,53.57) | 545.66(386.30,773.07) | 27.18(19.24,38.50) | -0.98(-1.40,-0.55) |
| Peru | 5666.18(4616.09,7468.33) | 68.26(55.61,89.97) | 3037.16(2213.53,4104.63) | 31.84(23.21,43.04) | -2.20(-2.40,-2.01) |
| Philippines | 12014.40(7978.29,15961.46) | 47.65(31.64,63.30) | 8519.00(6917.10,11427.10) | 25.06(20.35,33.61) | -1.53(-1.71,-1.34) |
| Poland | 3130.63(2995.15,3260.82) | 32.69(31.27,34.05) | 780.43(691.07,857.82) | 13.26(11.74,14.57) | -2.89(-3.30,-2.48) |
| Portugal | 774.40(666.15,890.38) | 36.60(31.48,42.08) | 173.57(139.73,209.05) | 12.74(10.26,15.35) | -4.34(-4.75,-3.94) |
| Puerto Rico | 441.87(388.22,502.14) | 44.37(38.99,50.43) | 55.91(46.20,66.88) | 12.58(10.40,15.05) | -3.50(-3.76,-3.24) |
| Qatar | 19.13(13.62,27.48) | 15.30(10.89,21.98) | 42.91(29.95,61.49) | 8.69(6.06,12.45) | -1.09(-1.58,-0.60) |
| Republic of Korea | 3951.76(3120.54,5198.01) | 34.75(27.44,45.71) | 543.98(395.64,733.91) | 8.96(6.51,12.08) | -4.52(-4.77,-4.27) |
| Republic of Moldova | 1003.49(857.66,1163.26) | 81.19(69.39,94.12) | 147.83(122.39,179.49) | 28.30(23.43,34.36) | -2.33(-2.85,-1.81) |
| Romania | 2841.48(2501.56,3194.04) | 51.03(44.93,57.36) | 507.08(432.86,593.24) | 16.85(14.38,19.71) | -3.68(-4.02,-3.33) |
| Russian Federation | 20582.13(19888.68,21253.38) | 59.32(57.32,61.25) | 3692.15(3429.06,3967.13) | 14.16(13.15,15.21) | -4.82(-5.30,-4.35) |
| Rwanda | 8291.23(5564.22,11575.42) | 244.37(164.00,341.17) | 4510.72(3066.44,7506.06) | 90.75(61.69,151.01) | -3.75(-4.06,-3.44) |
| Saint Kitts and Nevis | 9.14(8.09,10.29) | 64.75(57.29,72.84) | 4.25(3.59,5.00) | 43.11(36.49,50.77) | -1.01(-1.22,-0.81) |
| Saint Lucia | 37.28(31.46,44.43) | 72.33(61.04,86.21) | 15.62(12.43,19.54) | 52.62(41.85,65.80) | -1.40(-1.64,-1.15) |
| Saint Vincent and the Grenadines | 44.73(37.64,52.64) | 108.87(91.62,128.13) | 18.57(15.65,22.17) | 74.41(62.72,88.85) | -0.99(-1.36,-0.62) |
| Samoa | 27.71(18.48,39.19) | 38.89(25.94,55.00) | 21.08(13.00,35.43) | 26.37(16.26,44.31) | -1.25(-1.36,-1.13) |
| San Marino | 2.65(1.92,3.55) | 64.70(46.79,86.65) | 0.94(0.67,1.35) | 21.48(15.13,30.70) | -3.25(-3.36,-3.14) |
| Sao Tome and Principe | 63.30(44.80,89.91) | 111.70(79.06,158.66) | 28.20(16.44,46.60) | 36.23(21.12,59.87) | -3.15(-3.57,-2.72) |
| Saudi Arabia | 2636.95(1503.18,3814.71) | 40.24(22.94,58.21) | 948.29(566.53,1366.47) | 12.53(7.49,18.06) | -3.74(-3.84,-3.64) |
| Senegal | 4082.49(2779.11,5711.87) | 111.81(76.11,156.43) | 3257.92(2214.89,4903.50) | 51.22(34.82,77.09) | -2.46(-2.70,-2.21) |
| Serbia | 975.19(648.29,1257.51) | 44.96(29.89,57.98) | 135.34(98.07,193.42) | 10.19(7.39,14.57) | -5.13(-5.64,-4.61) |
| Seychelles | 4.18(3.12,5.96) | 17.63(13.13,25.12) | 2.53(1.83,3.39) | 10.83(7.80,14.48) | 0.10(-0.85,1.06) |
| Sierra Leone | 2670.93(1593.26,3797.56) | 147.35(87.90,209.51) | 2788.55(1925.33,4078.35) | 77.98(53.84,114.04) | -1.98(-2.14,-1.81) |
| Singapore | 188.16(159.81,225.73) | 28.98(24.61,34.76) | 96.00(79.23,115.32) | 11.82(9.76,14.20) | -2.36(-2.70,-2.02) |
| Slovakia | 387.33(305.56,498.50) | 29.22(23.05,37.60) | 141.46(106.10,194.69) | 16.51(12.39,22.73) | -1.77(-2.02,-1.52) |
| Slovenia | 110.59(95.75,125.50) | 26.74(23.15,30.35) | 42.27(33.88,52.49) | 13.53(10.85,16.81) | -1.38(-1.83,-0.94) |
| Solomon Islands | 20.41(11.11,32.23) | 13.11(7.13,20.70) | 43.07(28.86,64.78) | 16.56(11.10,24.91) | 0.89(0.59,1.20) |
| Somalia | 5492.87(2968.85,8107.47) | 141.00(76.21,208.12) | 7540.10(3800.76,12100.48) | 73.00(36.80,117.15) | -1.68(-2.06,-1.29) |
| South Africa | 3715.33(2503.52,5576.92) | 27.29(18.39,40.97) | 4022.72(3285.10,5016.58) | 26.46(21.61,32.99) | -0.31(-0.68,0.05) |
| South Sudan | 4720.59(2741.01,6758.07) | 179.89(104.45,257.53) | 6951.17(4282.50,9855.37) | 161.85(99.71,229.46) | 0.11(-0.61,0.83) |
| Spain | 2113.80(1805.40,2433.71) | 26.98(23.04,31.06) | 739.16(600.80,928.13) | 11.41(9.27,14.32) | -3.22(-3.44,-3.00) |
| Sri Lanka | 1326.16(972.51,1719.08) | 23.97(17.58,31.07) | 514.69(340.67,733.61) | 10.08(6.67,14.37) | -2.75(-3.32,-2.18) |
| Sudan | 7885.98(3094.74,15744.96) | 88.68(34.80,177.06) | 6185.94(4060.84,8763.18) | 37.29(24.48,52.82) | -2.48(-2.59,-2.36) |
| Suriname | 81.46(48.62,105.37) | 62.53(37.32,80.89) | 60.43(42.20,81.85) | 42.18(29.46,57.13) | -1.00(-1.33,-0.67) |
| Sweden | 238.33(211.61,270.44) | 15.43(13.70,17.51) | 117.68(99.92,138.90) | 6.46(5.49,7.63) | -1.86(-2.53,-1.19) |
| Switzerland | 275.51(240.89,317.25) | 23.84(20.84,27.45) | 128.27(105.77,155.90) | 9.63(7.94,11.70) | -3.31(-3.66,-2.96) |
| Syrian Arab Republic | 1941.21(1416.61,2482.01) | 32.78(23.92,41.91) | 638.67(482.68,866.33) | 17.43(13.18,23.65) | -1.95(-2.58,-1.31) |
| Taiwan (Province of China) | 1844.53(1647.89,2042.67) | 33.49(29.92,37.08) | 454.18(386.92,535.98) | 15.41(13.13,18.19) | -2.49(-2.85,-2.12) |
| Tajikistan | 2499.28(1115.38,3667.62) | 107.63(48.03,157.95) | 2145.82(1439.26,3115.06) | 59.87(40.15,86.91) | -2.46(-2.86,-2.06) |
| Thailand | 7295.17(5625.03,9743.55) | 43.27(33.37,57.80) | 1820.32(1341.63,2862.39) | 18.64(13.74,29.31) | -3.37(-3.78,-2.96) |
| Timor-Leste | 173.19(63.32,273.43) | 52.07(19.04,82.20) | 110.62(77.38,170.67) | 21.25(14.86,32.78) | -3.05(-3.48,-2.63) |
| Togo | 1518.44(1056.91,2163.82) | 86.15(59.97,122.77) | 1864.58(1228.44,2851.68) | 56.35(37.12,86.18) | -1.23(-1.37,-1.08) |
| Tokelau | 0.12(0.08,0.18) | 20.07(12.50,30.33) | 0.46(0.29,0.73) | 117.06(73.58,186.64) | 1.54(-0.17,3.28) |
| Tonga | 19.97(13.48,28.58) | 47.77(32.24,68.35) | 20.05(12.57,32.48) | 51.37(32.22,83.24) | -0.14(-0.57,0.29) |
| Trinidad and Tobago | 276.41(241.26,315.47) | 68.03(59.38,77.64) | 89.70(70.97,114.39) | 32.93(26.05,41.99) | -1.88(-2.28,-1.47) |
| Tunisia | 2394.35(1675.28,3118.31) | 77.10(53.95,100.41) | 825.25(586.92,1121.16) | 29.84(21.22,40.54) | -2.72(-2.83,-2.60) |
| Turkey | 12836.74(7782.56,17444.71) | 62.65(37.98,85.14) | 3999.77(3004.57,5283.18) | 21.60(16.22,28.53) | -3.37(-3.66,-3.08) |
| Turkmenistan | 329.10(283.40,378.70) | 21.93(18.88,25.23) | 221.63(182.44,270.25) | 14.54(11.97,17.73) | -1.83(-2.33,-1.33) |
| Tuvalu | 0.95(0.53,1.43) | 27.35(15.15,41.05) | 0.62(0.43,0.89) | 16.58(11.54,23.97) | -1.27(-1.43,-1.10) |
| Uganda | 18046.19(12372.25,25140.95) | 214.33(146.95,298.60) | 37563.42(22580.24,61705.64) | 189.37(113.84,311.08) | -0.22(-0.47,0.02) |
| Ukraine | 3479.05(2426.27,4306.37) | 30.59(21.33,37.86) | 1391.58(1155.07,1688.29) | 21.93(18.20,26.61) | -1.26(-1.67,-0.85) |
| United Arab Emirates | 139.85(88.63,213.46) | 23.73(15.04,36.22) | 120.13(88.32,165.89) | 8.97(6.60,12.39) | -2.70(-3.05,-2.35) |
| United Kingdom | 1846.41(1777.99,1923.32) | 16.91(16.28,17.61) | 938.85(859.66,1022.16) | 7.97(7.30,8.67) | -2.37(-2.67,-2.07) |
| United Republic of Tanzania | 23027.97(15776.43,30613.37) | 190.70(130.65,253.52) | 28171.53(18569.50,43830.24) | 115.44(76.10,179.61) | -1.05(-1.33,-0.78) |
| United States Virgin Islands | 16.47(12.33,22.10) | 51.55(38.61,69.19) | 1.78(1.11,2.77) | 13.29(8.29,20.71) | -3.36(-3.82,-2.89) |
| United States of America | 9141.93(8881.89,9465.38) | 16.35(15.89,16.93) | 3856.03(3551.86,4157.01) | 6.49(5.98,6.99) | -3.05(-3.21,-2.90) |
| Uruguay | 285.76(246.57,331.40) | 34.91(30.12,40.48) | 130.48(105.69,162.60) | 19.78(16.03,24.65) | -2.23(-2.76,-1.69) |
| Uzbekistan | 4410.47(3057.57,5750.09) | 51.55(35.74,67.21) | 3605.36(2819.76,4619.85) | 35.73(27.94,45.78) | -0.97(-1.24,-0.69) |
| Vanuatu | 7.23(4.29,11.55) | 10.61(6.31,16.96) | 15.97(10.41,23.55) | 13.70(8.93,20.21) | 1.13(0.69,1.57) |
| Venezuela (Bolivarian Republic of) | 3040.30(2837.81,3261.55) | 42.86(40.00,45.98) | 2214.80(1731.25,2771.11) | 33.44(26.14,41.83) | -0.75(-1.22,-0.28) |
| Viet Nam | 10639.43(7062.76,14546.50) | 40.13(26.64,54.87) | 6328.50(4163.61,9040.53) | 25.56(16.81,36.51) | -1.14(-1.30,-0.98) |
| Yemen | 2768.05(1552.00,5258.54) | 39.02(21.88,74.12) | 2895.43(1919.89,4562.57) | 21.00(13.92,33.09) | -1.88(-2.01,-1.74) |
| Zambia | 7829.53(5401.74,10387.42) | 208.54(143.87,276.66) | 8338.29(5001.90,13967.83) | 100.81(60.47,168.87) | -2.18(-2.34,-2.03) |
| Zimbabwe | 1871.94(904.54,2716.50) | 38.87(18.78,56.40) | 5650.93(2567.75,8184.76) | 89.79(40.80,130.04) | 5.22(4.03,6.42) |
| ^a^EAPC is expressed as 95% CIs | | | | | |

**Table S7** Prediction of Incidence of Hodgkin Lymphoma and Non-Hodgkin Lymphoma in children to 2035.

| Year | Hodgkin Lymphoma | | | Non-Hodgkin Lymphoma | | |
| --- | --- | --- | --- | --- | --- | --- |
|  | Both, %(UIs) | Boy, %(UIs) | Girl, %(UIs) | Both, %(UIs) | Boy, %(UIs) | Girl, %(UIs) |
| 2021 | 0.21(0.21, 0.21) | 0.26(0.26, 0.26) | 0.16(0.16, 0.16) | 1.03(1.03, 1.03) | 1.31(1.31, 1.31) | 0.74(0.74, 0.74) |
| 2022 | 0.21(0.21, 0.22) | 0.26(0.26, 0.27) | 0.16(0.16, 0.16) | 1.02(0.99, 1.04) | 1.31(1.28, 1.34) | 0.71(0.69, 0.73) |
| 2023 | 0.21(0.20, 0.22) | 0.26(0.25, 0.27) | 0.16(0.15, 0.16) | 1.00(0.96, 1.05) | 1.30(1.24, 1.36) | 0.70(0.68, 0.73) |
| 2024 | 0.21(0.20, 0.22) | 0.26(0.25, 0.27) | 0.16(0.15, 0.17) | 1.00(0.93, 1.07) | 1.29(1.21, 1.36) | 0.70(0.66, 0.74) |
| 2025 | 0.21(0.20, 0.22) | 0.26(0.24, 0.27) | 0.16(0.15, 0.17) | 0.99(0.91, 1.08) | 1.28(1.19, 1.37) | 0.70(0.66, 0.75) |
| 2026 | 0.21(0.20, 0.22) | 0.26(0.24, 0.27) | 0.16(0.15, 0.17) | 0.99(0.89, 1.10) | 1.27(1.17, 1.37) | 0.71(0.67, 0.76) |
| 2027 | 0.21(0.20, 0.22) | 0.26(0.24, 0.27) | 0.16(0.15, 0.17) | 0.99(0.88, 1.12) | 1.26(1.15, 1.37) | 0.73(0.68, 0.78) |
| 2028 | 0.21(0.19, 0.22) | 0.25(0.24, 0.27) | 0.16(0.15, 0.17) | 0.99(0.86, 1.13) | 1.25(1.13, 1.37) | 0.74(0.69, 0.80) |
| 2029 | 0.21(0.19, 0.22) | 0.25(0.23, 0.27) | 0.16(0.15, 0.17) | 0.99(0.85, 1.15) | 1.24(1.11, 1.37) | 0.76(0.71, 0.81) |
| 2030 | 0.20(0.19, 0.22) | 0.25(0.23, 0.27) | 0.16(0.14, 0.17) | 0.99(0.84, 1.16) | 1.23(1.10, 1.37) | 0.77(0.72, 0.83) |
| 2031 | 0.20(0.19, 0.22) | 0.25(0.23, 0.27) | 0.16(0.14, 0.17) | 0.98(0.82, 1.18) | 1.22(1.08, 1.37) | 0.78(0.72, 0.84) |
| 2032 | 0.20(0.19, 0.22) | 0.25(0.23, 0.27) | 0.16(0.14, 0.17) | 0.98(0.81, 1.19) | 1.21(1.07, 1.37) | 0.78(0.73, 0.85) |
| 2033 | 0.20(0.18, 0.22) | 0.25(0.22, 0.27) | 0.16(0.14, 0.18) | 0.98(0.80, 1.20) | 1.20(1.05, 1.36) | 0.78(0.72, 0.85) |
| 2034 | 0.2(0.18, 0.22) | 0.25(0.22, 0.27) | 0.16(0.14, 0.18) | 0.98(0.80, 1.22) | 1.19(1.04, 1.36) | 0.78(0.72, 0.84) |
| 2035 | 0.20(0.18, 0.22) | 0.24(0.22, 0.27) | 0.16(0.14, 0.18) | 0.98(0.79, 1.23) | 1.18(1.02, 1.36) | 0.77(0.71, 0.84) |

**Table S8** Prediction of Deaths of Hodgkin Lymphoma and Non-Hodgkin Lymphoma in children to 2035.

| Year | Hodgkin Lymphoma | | | Non-Hodgkin Lymphoma | | |
| --- | --- | --- | --- | --- | --- | --- |
|  | Both, %(UIs) | Boy, %(UIs) | Girl, %(UIs) | Both, %(UIs) | Boy, %(UIs) | Girl, %(UIs) |
| 2021 | 0.1(0.1, 0.1) | 0.12(0.12, 0.12) | 0.07(0.07, 0.07) | 0.45(0.45, 0.45) | 0.58(0.58, 0.58) | 0.31(0.31, 0.31) |
| 2022 | 0.09(0.09, 0.09) | 0.12(0.12, 0.12) | 0.07(0.07, 0.07) | 0.44(0.43, 0.44) | 0.56(0.55, 0.57) | 0.3(0.3, 0.31) |
| 2023 | 0.09(0.09, 0.1) | 0.12(0.11, 0.12) | 0.07(0.06, 0.07) | 0.43(0.41, 0.44) | 0.55(0.53, 0.57) | 0.29(0.28, 0.31) |
| 2024 | 0.09(0.09, 0.09) | 0.11(0.11, 0.12) | 0.07(0.06, 0.07) | 0.42(0.4, 0.44) | 0.54(0.51, 0.57) | 0.29(0.27, 0.31) |
| 2025 | 0.09(0.08, 0.09) | 0.11(0.11, 0.12) | 0.06(0.06, 0.07) | 0.41(0.38, 0.44) | 0.53(0.49, 0.57) | 0.28(0.26, 0.31) |
| 2026 | 0.09(0.08, 0.09) | 0.11(0.1, 0.12) | 0.06(0.06, 0.07) | 0.4(0.37, 0.44) | 0.52(0.48, 0.57) | 0.28(0.25, 0.31) |
| 2027 | 0.09(0.08, 0.09) | 0.11(0.1, 0.12) | 0.06(0.06, 0.07) | 0.4(0.36, 0.44) | 0.51(0.47, 0.56) | 0.28(0.25, 0.31) |
| 2028 | 0.09(0.08, 0.09) | 0.11(0.1, 0.12) | 0.06(0.06, 0.07) | 0.39(0.35, 0.44) | 0.5(0.45, 0.56) | 0.27(0.24, 0.31) |
| 2029 | 0.08(0.08, 0.09) | 0.11(0.1, 0.12) | 0.06(0.06, 0.07) | 0.38(0.34, 0.43) | 0.49(0.44, 0.55) | 0.27(0.23, 0.31) |
| 2030 | 0.08(0.08, 0.09) | 0.1(0.09, 0.11) | 0.06(0.06, 0.07) | 0.38(0.33, 0.43) | 0.48(0.43, 0.54) | 0.26(0.23, 0.31) |
| 2031 | 0.08(0.07, 0.09) | 0.1(0.09, 0.11) | 0.06(0.05, 0.07) | 0.37(0.32, 0.43) | 0.47(0.42, 0.54) | 0.26(0.22, 0.31) |
| 2032 | 0.08(0.07, 0.09) | 0.1(0.09, 0.11) | 0.06(0.05, 0.06) | 0.37(0.32, 0.42) | 0.47(0.41, 0.53) | 0.26(0.21, 0.31) |
| 2033 | 0.08(0.07, 0.09) | 0.1(0.09, 0.11) | 0.06(0.05, 0.06) | 0.36(0.31, 0.42) | 0.46(0.4, 0.53) | 0.25(0.21, 0.31) |
| 2034 | 0.08(0.07, 0.09) | 0.1(0.09, 0.11) | 0.06(0.05, 0.06) | 0.35(0.3, 0.41) | 0.45(0.39, 0.52) | 0.25(0.21, 0.31) |
| 2035 | 0.08(0.07, 0.09) | 0.1(0.08, 0.11) | 0.06(0.05, 0.06) | 0.35(0.29, 0.41) | 0.44(0.38, 0.51) | 0.25(0.2, 0.31) |

**Table S9** Prediction of DALYs of Hodgkin Lymphoma and Non-Hodgkin Lymphoma in children to 2035.

| Year | Hodgkin Lymphoma | | | Non-Hodgkin Lymphoma | | |
| --- | --- | --- | --- | --- | --- | --- |
|  | Both, %(UIs) | Boy, %(UIs) | Girl, %(UIs) | Both, %(UIs) | Boy, %(UIs) | Girl, %(UIs) |
| 2021 | 7.78(7.78, 7.78) | 9.97(9.97, 9.97) | 5.45(5.45, 5.45) | 37.83(37.83, 37.83) | 48.86(48.86, 48.86) | 26.07(26.07, 26.07) |
| 2022 | 7.61(7.48, 7.74) | 9.72(9.53, 9.92) | 5.36(5.26, 5.45) | 36.51(35.92, 37.11) | 47.35(46.58, 48.13) | 25.35(24.85, 25.85) |
| 2023 | 7.49(7.24, 7.75) | 9.56(9.19, 9.94) | 5.29(5.11, 5.47) | 35.37(34.2, 36.57) | 46.38(44.58, 48.26) | 24.76(23.81, 25.75) |
| 2024 | 7.38(7.05, 7.72) | 9.4(8.93, 9.9) | 5.22(4.99, 5.46) | 34.37(32.64, 36.18) | 45.5(42.9, 48.26) | 24.26(22.87, 25.73) |
| 2025 | 7.26(6.88, 7.66) | 9.25(8.7, 9.83) | 5.15(4.88, 5.44) | 33.47(31.21, 35.89) | 44.64(41.49, 48.03) | 23.82(22.03, 25.76) |
| 2026 | 7.15(6.73, 7.6) | 9.09(8.48, 9.75) | 5.08(4.78, 5.41) | 32.66(29.91, 35.66) | 43.79(40.21, 47.68) | 23.43(21.27, 25.82) |
| 2027 | 7.04(6.58, 7.54) | 8.94(8.27, 9.67) | 5.02(4.69, 5.37) | 31.92(28.72, 35.47) | 42.96(39.04, 47.27) | 23.08(20.59, 25.88) |
| 2028 | 6.93(6.44, 7.47) | 8.8(8.08, 9.57) | 4.95(4.6, 5.34) | 31.23(27.63, 35.3) | 42.14(37.94, 46.81) | 22.75(19.96, 25.93) |
| 2029 | 6.83(6.31, 7.39) | 8.65(7.9, 9.48) | 4.89(4.51, 5.3) | 30.58(26.62, 35.13) | 41.34(36.9, 46.32) | 22.44(19.39, 25.98) |
| 2030 | 6.72(6.18, 7.32) | 8.51(7.72, 9.37) | 4.82(4.43, 5.25) | 29.97(25.69, 34.96) | 40.55(35.91, 45.81) | 22.14(18.85, 26.01) |
| 2031 | 6.62(6.05, 7.24) | 8.37(7.55, 9.27) | 4.76(4.35, 5.21) | 29.38(24.81, 34.79) | 39.78(34.96, 45.28) | 21.86(18.35, 26.03) |
| 2032 | 6.52(5.93, 7.16) | 8.23(7.38, 9.17) | 4.7(4.27, 5.17) | 28.82(24, 34.62) | 39.03(34.05, 44.73) | 21.58(17.89, 26.04) |
| 2033 | 6.42(5.81, 7.09) | 8.09(7.22, 9.06) | 4.64(4.2, 5.12) | 28.28(23.23, 34.44) | 38.29(33.18, 44.18) | 21.31(17.44, 26.04) |
| 2034 | 6.32(5.7, 7.01) | 7.96(7.07, 8.96) | 4.58(4.13, 5.08) | 27.76(22.51, 34.24) | 37.56(32.34, 43.62) | 21.05(17.03, 26.02) |
| 2035 | 6.22(5.59, 6.93) | 7.83(6.92, 8.85) | 4.52(4.06, 5.03) | 27.25(21.82, 34.04) | 36.85(31.53, 43.06) | 20.79(16.63, 25.99) |

**Table S10** Autoregressive Integrated Moving Average (ARIMA) Model Parameters and Their Corresponding AIC and BIC for Prediction of Incidence, Deaths and YLDs rate for Hodgkin lymphom and Non-Hodgkin lymphoma in Children to 2035.

| Measures | Hodgkin Lymphoma | | | | | Non-Hodgkin Lymphoma | | | | |
| --- | --- | --- | --- | --- | --- | --- | --- | --- | --- | --- |
|  | Sex | Parameters | AIC | BIC | Ljung-Box test p-value | Sex | Parameters | AIC | BIC | Ljung-Box test p-value |
| Incidence | Boys | ARIMA (0,1,1) | -197.58 | -193.28 | 0.81 | Boys | ARIMA (0,1,1) | -183.64 | -179.34 | 0.30 |
|  | Girls | ARIMA (0,1,1) | -193.42 | -190.55 | 0.96 | Girls | ARIMA (3,0,0) | -180.70 | -173.37 | 0.67 |
|  | Both | ARIMA (0,1,1) | -203.21 | -198.91 | 0.77 | Both | ARIMA (1,1,0) | -183.66 | -180.80 | 0.80 |
| Deaths | Boys | ARIMA (0,1,1) | -192.44 | -188.14 | 0.82 | Boys | ARIMA (0,1,2) | -202.42 | -196.69 | 0.19 |
|  | Girls | ARIMA (0,1,1) | -198.77 | -194.47 | 0.82 | Girls | ARIMA (1,1,0) | -193.56 | -189.26 | 0.04 |
|  | Both | ARIMA (0,1,1) | -200.66 | -196.36 | 0.76 | Both | ARIMA (1,1,1) | -205.41 | -199.67 | 0.17 |
| DALYs | Boys | ARIMA (0,1,1) | -192.45 | -188.15 | 0.84 | Boys | ARIMA (0,1,2) | -202.42 | -196.69 | 0.19 |
|  | Girls | ARIMA (0,1,1) | -198.72 | -194.42 | 0.78 | Girls | ARIMA (1,1,0) | -192.80 | -188.50 | 0.05 |
|  | Both | ARIMA (0,1,1) | -200.45 | -196.15 | 0.76 | Both | ARIMA (1,1,0) | -204.21 | -199.90 | 0.10 |

**Table S11** Country Priority Index for childhood Hodgkin and non-Hodgkin lymphoma across 204 countries and territories.

| Rank | Country or territory | Tier | Rate-based burden score | Population- burden score | Trajectory score | CPI | Sensitivity rank range |
| --- | --- | --- | --- | --- | --- | --- | --- |
| 1 | Pakistan | T1 | 98.15 | 99.51 | 92.36 | 96.67 | 1–2 |
| 2 | Uganda | T1 | 99.26 | 97.78 | 86.02 | 94.36 | 2–4 |
| 3 | South Sudan | T1 | 98.77 | 90.15 | 89.90 | 92.94 | 3–6 |
| 4 | Zimbabwe | T1 | 89.90 | 84.98 | 99.88 | 91.58 | 2–16 |
| 5 | Nigeria | T1 | 97.91 | 100.00 | 76.79 | 91.56 | 1–9 |
| 6 | United Republic of Tanzania | T1 | 96.18 | 97.04 | 76.79 | 90.00 | 4–12 |
| 7 | Côte d'Ivoire | T1 | 89.96 | 91.63 | 84.54 | 88.71 | 6–11 |
| 8 | Somalia | T1 | 91.56 | 92.86 | 71.18 | 85.20 | 8–19 |
| 9 | Kenya | T1 | 75.80 | 90.64 | 88.67 | 85.04 | 5–21 |
| 10 | Libya | T1 | 89.29 | 70.44 | 91.38 | 83.70 | 5–25 |
| 11 | Madagascar | T1 | 89.22 | 93.60 | 65.83 | 82.88 | 10–24 |
| 12 | Eritrea | T1 | 96.12 | 79.56 | 72.29 | 82.66 | 9–20 |
| 13 | Malawi† | T1 | 95.75 | 93.10 | 57.33 | 82.06 | 7–27 |
| 14 | Zambia | T1 | 94.15 | 93.35 | 54.06 | 80.52 | 8–32 |
| 15 | Haiti | T1 | 89.53 | 82.02 | 69.83 | 80.46 | 14–22 |
| 16 | Afghanistan | T1 | 77.59 | 88.67 | 70.94 | 79.06 | 13–31 |
| 17 | Namibia | T1 | 83.62 | 55.67 | 95.14 | 78.14 | 7–50 |
| 18 | Uzbekistan | T1 | 77.09 | 81.77 | 72.97 | 77.28 | 19–28 |
| 19 | Guinea† | T1 | 87.81 | 83.99 | 59.98 | 77.26 | 16–33 |
| 20 | Venezuela (Bolivarian Republic of) | T1 | 74.32 | 77.09 | 79.25 | 76.89 | 15–29 |
| 21 | South Africa | T1 | 64.78 | 81.53 | 84.36 | 76.89 | 9–37 |
| 22 | Lesotho | T2 | 79.00 | 47.78 | 98.77 | 75.18 | 8–63 |
| 23 | Burundi | T2 | 92.55 | 87.68 | 44.46 | 74.90 | 13–44 |
| 24 | Mali† | T2 | 84.61 | 89.66 | 50.18 | 74.82 | 17–46 |
| 25 | Chad | T2 | 67.92 | 80.79 | 74.82 | 74.51 | 17–36 |
| 26 | Central African Republic | T2 | 77.34 | 66.50 | 79.62 | 74.49 | 22–41 |
| 27 | Ethiopia | T2 | 94.21 | 98.28 | 29.68 | 74.06 | 5–65 |
| 28 | Sudan | T2 | 79.43 | 89.90 | 50.74 | 73.36 | 20–54 |
| 29 | Bangladesh | T2 | 83.50 | 96.80 | 38.67 | 72.99 | 12–70 |
| 30 | Djibouti | T2 | 91.26 | 56.40 | 69.70 | 72.45 | 21–52 |
| 31 | Comoros | T2 | 94.95 | 48.77 | 72.35 | 72.02 | 16–60 |
| 32 | Iran (Islamic Republic of) | T2 | 54.62 | 82.02 | 79.19 | 71.94 | 12–54 |
| 33 | Viet Nam | T2 | 59.36 | 86.45 | 68.84 | 71.55 | 18–58 |
| 34 | Yemen | T2 | 66.69 | 82.76 | 63.61 | 71.02 | 28–53 |
| 35 | Botswana | T2 | 72.29 | 45.32 | 92.86 | 70.16 | 17–74 |
| 36 | Eswatini | T2 | 77.59 | 41.38 | 90.95 | 69.97 | 14–72 |
| 37 | Burkina Faso | T2 | 60.90 | 81.53 | 65.52 | 69.31 | 25–63 |
| 38 | Cameroon | T2 | 59.73 | 83.50 | 63.36 | 68.86 | 26–67 |
| 39 | Gambia | T2 | 84.73 | 59.61 | 61.88 | 68.74 | 33–59 |
| 40 | Azerbaijan | T2 | 76.05 | 67.73 | 62.25 | 68.68 | 36–46 |
| 41 | Democratic Republic of the Congo | T2 | 58.37 | 90.89 | 53.82 | 67.69 | 30–81 |
| 42 | Ecuador | T2 | 62.19 | 70.20 | 69.52 | 67.30 | 36–57 |
| 43 | Rwanda | T2 | 93.78 | 85.71 | 20.14 | 66.54 | 14–84 |
| 44 | Paraguay | T2 | 64.84 | 58.37 | 74.88 | 66.03 | 37–67 |
| 45 | Philippines | T2 | 40.27 | 85.71 | 70.63 | 65.54 | 16–84 |
| 46 | Mozambique† | T2 | 58.37 | 83.50 | 52.52 | 64.80 | 38–83 |
| 47 | Palestine | T2 | 60.41 | 58.62 | 75.37 | 64.80 | 40–71 |
| 48 | Fiji | T2 | 65.15 | 31.53 | 97.48 | 64.72 | 20–103 |
| 49 | Algeria | T2 | 74.63 | 85.96 | 32.76 | 64.45 | 24–89 |
| 50 | Jordan | T2 | 62.87 | 66.75 | 63.24 | 64.29 | 46–64 |
| 51 | Ukraine | T2 | 60.59 | 71.43 | 60.59 | 64.20 | 44–72 |
| 52 | Dominica | T3 | 83.19 | 12.56 | 96.31 | 64.02 | 6–106 |
| 53 | Colombia | T3 | 63.73 | 78.08 | 48.15 | 63.32 | 46–82 |
| 54 | Nepal† | T3 | 73.52 | 81.03 | 34.67 | 63.07 | 27–86 |
| 55 | Benin | T3 | 59.24 | 71.67 | 58.13 | 63.01 | 47–73 |
| 56 | Panama | T3 | 66.26 | 50.00 | 72.66 | 62.97 | 39–76 |
| 57 | Papua New Guinea | T3 | 42.30 | 56.90 | 88.49 | 62.56 | 30–99 |
| 58 | Turkmenistan | T3 | 58.74 | 56.90 | 70.63 | 62.09 | 50–78 |
| 59 | India | T3 | 61.64 | 99.01 | 25.55 | 62.07 | 23–124 |
| 60 | Morocco | T3 | 63.73 | 78.08 | 43.78 | 61.86 | 48–88 |
| 61 | Indonesia† | T3 | 40.39 | 91.13 | 53.02 | 61.51 | 32–115 |
| 62 | Peru | T3 | 64.04 | 75.86 | 44.09 | 61.33 | 49–87 |
| 63 | Tajikistan | T3 | 66.93 | 67.49 | 48.34 | 60.92 | 56–76 |
| 64 | Iraq | T3 | 66.56 | 81.77 | 31.83 | 60.06 | 33–105 |
| 65 | Guyana | T3 | 63.67 | 25.37 | 90.64 | 59.89 | 25–114 |
| 66 | Gabon | T3 | 59.24 | 38.18 | 82.20 | 59.87 | 37–102 |
| 67 | Angola | T3 | 65.33 | 82.51 | 31.40 | 59.75 | 34–107 |
| 68 | Bolivia (Plurinational State of) | T3 | 80.54 | 72.66 | 25.92 | 59.71 | 28–99 |
| 69 | Belarus | T3 | 54.25 | 49.51 | 72.97 | 58.91 | 57–92 |
| 70 | Tokelau† | T3 | 80.97 | 0.99 | 93.23 | 58.39 | 11–126 |
| 71 | Tunisia | T3 | 75.74 | 68.97 | 29.80 | 58.17 | 39–98 |
| 72 | Suriname | T3 | 70.32 | 23.40 | 79.74 | 57.82 | 29–107 |
| 73 | Mexico | T3 | 56.90 | 89.90 | 26.60 | 57.80 | 36–134 |
| 74 | Argentina | T3 | 47.84 | 73.40 | 51.85 | 57.70 | 53–102 |
| 75 | Niue | T3 | 74.75 | 1.23 | 96.98 | 57.66 | 13–137 |
| 76 | Armenia | T3 | 54.50 | 36.45 | 79.74 | 56.90 | 48–109 |
| 77 | Sierra Leone | T3 | 59.85 | 64.78 | 45.81 | 56.81 | 65–93 |
| 78 | Congo | T3 | 58.00 | 53.94 | 56.34 | 56.10 | 75–83 |
| 79 | Costa Rica | T3 | 67.36 | 50.25 | 49.63 | 55.75 | 72–96 |
| 80 | Togo | T3 | 54.13 | 59.85 | 51.23 | 55.07 | 73–96 |
| 81 | Brazil | T3 | 44.40 | 90.89 | 28.63 | 54.64 | 55–148 |
| 82 | Myanmar | T3 | 58.00 | 81.03 | 24.32 | 54.45 | 51–135 |
| 83 | Malaysia | T3 | 39.41 | 67.73 | 55.79 | 54.31 | 55–113 |
| 84 | El Salvador | T3 | 53.88 | 55.42 | 51.66 | 53.65 | 80–95 |
| 85 | Oman | T3 | 53.20 | 45.57 | 61.58 | 53.45 | 77–100 |
| 86 | Samoa | T3 | 68.47 | 21.18 | 69.89 | 53.18 | 40–114 |
| 87 | Saint Vincent and the Grenadines | T3 | 74.75 | 14.78 | 69.46 | 53.00 | 35–134 |
| 88 | Cuba | T3 | 60.28 | 54.19 | 42.18 | 52.22 | 79–107 |
| 89 | Bhutan† | T3 | 80.91 | 33.50 | 41.50 | 51.97 | 69–151 |
| 90 | Turkey | T3 | 57.33 | 81.53 | 15.64 | 51.50 | 52–149 |
| 91 | Niger | T3 | 56.96 | 81.53 | 15.21 | 51.23 | 53–150 |
| 92 | Lebanon | T3 | 66.01 | 55.42 | 32.02 | 51.15 | 69–127 |
| 93 | Democratic People's Republic of Korea | T3 | 42.67 | 62.32 | 47.60 | 50.86 | 76–119 |
| 94 | Saint Lucia | T3 | 73.34 | 14.78 | 63.24 | 50.45 | 44–144 |
| 95 | Senegal | T3 | 54.37 | 68.97 | 27.77 | 50.37 | 66–137 |
| 96 | Cambodia | T3 | 52.09 | 66.75 | 31.83 | 50.23 | 73–133 |
| 97 | Guatemala | T3 | 43.29 | 64.04 | 43.04 | 50.12 | 81–130 |
| 98 | Grenada | T3 | 61.21 | 12.56 | 73.58 | 49.12 | 47–139 |
| 99 | Mauritania | T3 | 50.92 | 50.74 | 45.57 | 49.08 | 93–108 |
| 100 | Liberia | T3 | 58.37 | 57.39 | 31.03 | 48.93 | 77–125 |
| 101 | Lao People's Democratic Republic | T3 | 56.34 | 54.43 | 34.79 | 48.52 | 85–121 |
| 102 | Guam | T3 | 41.87 | 12.07 | 90.89 | 48.28 | 50–165 |
| 103 | Malta | T3 | 59.17 | 17.49 | 67.49 | 48.05 | 62–135 |
| 104 | Cabo Verde | T3 | 42.98 | 17.00 | 83.74 | 47.91 | 61–154 |
| 105 | Solomon Islands | T3 | 30.54 | 20.69 | 90.76 | 47.33 | 65–168 |
| 106 | Nauru | T3 | 47.41 | 3.82 | 90.27 | 47.17 | 42–167 |
| 107 | Tonga† | T3 | 43.10 | 12.32 | 84.98 | 46.80 | 59–162 |
| 108 | Marshall Islands | T3 | 35.16 | 8.50 | 94.33 | 46.00 | 55–177 |
| 109 | Kuwait | T3 | 45.63 | 37.19 | 53.76 | 45.53 | 103–124 |
| 110 | Barbados | T3 | 52.09 | 13.79 | 70.57 | 45.48 | 68–149 |
| 111 | Republic of Moldova | T3 | 68.97 | 40.64 | 26.79 | 45.46 | 86–167 |
| 112 | Mongolia | T3 | 62.50 | 49.26 | 24.38 | 45.38 | 84–152 |
| 113 | Lithuania | T3 | 47.23 | 28.82 | 60.10 | 45.38 | 90–136 |
| 114 | Kyrgyzstan | T3 | 46.06 | 54.68 | 34.42 | 45.05 | 95–139 |
| 115 | Saint Kitts and Nevis† | T3 | 42.00 | 6.53 | 86.33 | 44.95 | 57–173 |
| 116 | Guinea-Bissau | T3 | 58.13 | 45.81 | 30.42 | 44.79 | 91–148 |
| 117 | China | T3 | 32.64 | 97.29 | 2.34 | 44.09 | 61–190 |
| 118 | Chile | T3 | 30.17 | 54.43 | 47.66 | 44.09 | 91–143 |
| 119 | Vanuatu | T3 | 23.46 | 14.78 | 93.41 | 43.88 | 75–186 |
| 120 | Dominican Republic | T3 | 39.72 | 49.51 | 40.33 | 43.19 | 111–140 |
| 121 | Brunei Darussalam | T3 | 38.36 | 16.63 | 74.45 | 43.14 | 80–163 |
| 122 | Syrian Arab Republic | T3 | 22.41 | 45.20 | 59.85 | 42.49 | 86–146 |
| 123 | American Samoa | T3 | 25.12 | 3.94 | 98.03 | 42.36 | 66–196 |
| 124 | Russian Federation | T3 | 40.33 | 81.53 | 3.82 | 41.89 | 68–181 |
| 125 | Kazakhstan | T3 | 45.75 | 66.26 | 13.36 | 41.79 | 82–161 |
| 126 | Uruguay | T3 | 48.52 | 36.21 | 40.46 | 41.73 | 115–146 |
| 127 | Slovakia | T3 | 42.86 | 38.42 | 42.86 | 41.38 | 124–136 |
| 128 | Germany | T3 | 17.18 | 66.50 | 38.98 | 40.89 | 84–164 |
| 129 | Trinidad and Tobago | T3 | 50.62 | 26.60 | 45.26 | 40.83 | 109–157 |
| 130 | North Macedonia | T3 | 51.35 | 30.05 | 39.78 | 40.39 | 117–160 |
| 131 | Mauritius | T3 | 30.48 | 19.95 | 69.95 | 40.13 | 101–170 |
| 132 | Egypt† | T3 | 19.09 | 79.06 | 21.43 | 39.86 | 95–184 |
| 133 | Estonia | T3 | 46.18 | 23.65 | 49.57 | 39.80 | 111–156 |
| 134 | Micronesia (Federated States of) | T3 | 30.36 | 9.85 | 78.82 | 39.68 | 85–183 |
| 135 | Italy | T3 | 33.31 | 66.26 | 19.33 | 39.63 | 98–170 |
| 136 | Bulgaria | T3 | 47.48 | 41.26 | 29.25 | 39.33 | 116–159 |
| 137 | Bahamas | T3 | 45.32 | 16.87 | 55.60 | 39.27 | 100–161 |
| 138 | Bosnia and Herzegovina | T3 | 38.12 | 28.08 | 50.92 | 39.04 | 121–153 |
| 139 | Albania | T3 | 47.60 | 30.30 | 39.16 | 39.02 | 126–162 |
| 140 | Japan | T3 | 10.78 | 59.85 | 46.06 | 38.90 | 82–163 |
| 141 | Greece | T3 | 25.31 | 36.95 | 53.51 | 38.59 | 118–151 |
| 142 | United Kingdom | T3 | 13.30 | 64.78 | 37.44 | 38.51 | 90–171 |
| 143 | Saudi Arabia | T3 | 34.17 | 67.24 | 12.44 | 37.95 | 94–176 |
| 144 | Bahrain | T3 | 44.83 | 26.11 | 41.75 | 37.56 | 129–166 |
| 145 | Thailand | T3 | 33.07 | 67.49 | 11.95 | 37.50 | 97–178 |
| 146 | Timor-Leste | T3 | 47.84 | 31.28 | 33.19 | 37.44 | 130–172 |
| 147 | Antigua and Barbuda† | T3 | 33.37 | 7.51 | 71.24 | 37.38 | 97–181 |
| 148 | France | T3 | 18.53 | 66.75 | 25.92 | 37.07 | 112–180 |
| 149 | Equatorial Guinea† | T3 | 56.71 | 35.34 | 19.03 | 37.03 | 108–184 |
| 150 | Jamaica | T3 | 52.65 | 36.21 | 22.04 | 36.97 | 115–178 |
| 151 | Georgia | T3 | 54.31 | 41.63 | 14.90 | 36.95 | 105–180 |
| 152 | Monaco† | T3 | 52.40 | 6.16 | 52.09 | 36.88 | 98–179 |
| 153 | Netherlands | T3 | 23.89 | 49.75 | 36.76 | 36.80 | 128–157 |
| 154 | Northern Mariana Islands | T3 | 7.51 | 2.96 | 99.01 | 36.49 | 91–203 |
| 155 | Qatar | T3 | 17.92 | 24.14 | 67.24 | 36.43 | 113–179 |
| 156 | Nicaragua | T3 | 36.27 | 48.03 | 24.63 | 36.31 | 121–160 |
| 157 | Latvia | T3 | 26.72 | 21.18 | 60.04 | 35.98 | 127–175 |
| 158 | United States of America | T3 | 9.91 | 79.80 | 17.92 | 35.88 | 102–195 |
| 159 | Czechia | T3 | 30.23 | 43.84 | 32.51 | 35.53 | 138–155 |
| 160 | Bermuda | T3 | 43.04 | 6.65 | 56.16 | 35.28 | 104–176 |
| 161 | Kiribati† | T3 | 14.78 | 9.61 | 81.03 | 35.14 | 110–198 |
| 162 | Ghana | T3 | 39.78 | 60.84 | 2.22 | 34.28 | 96–183 |
| 163 | Belgium | T3 | 22.91 | 42.36 | 37.32 | 34.20 | 139–163 |
| 164 | Romania | T3 | 42.18 | 54.43 | 5.17 | 33.93 | 104–178 |
| 165 | Seychelles | T3 | 16.69 | 7.76 | 76.23 | 33.56 | 116–197 |
| 166 | Israel | T3 | 24.08 | 46.31 | 28.82 | 33.07 | 144–168 |
| 167 | Tuvalu | T3 | 28.02 | 2.22 | 66.32 | 32.18 | 114–194 |
| 168 | Sri Lanka | T3 | 21.12 | 56.40 | 18.97 | 32.16 | 133–185 |
| 169 | United Arab Emirates | T3 | 27.83 | 38.79 | 29.31 | 31.98 | 147–169 |
| 170 | Croatia | T3 | 29.19 | 28.08 | 38.05 | 31.77 | 153–171 |
| 171 | Poland | T3 | 25.49 | 58.62 | 11.08 | 31.73 | 122–188 |
| 172 | Honduras | T3 | 28.33 | 52.96 | 13.36 | 31.55 | 129–183 |
| 173 | Spain | T3 | 21.49 | 60.59 | 12.44 | 31.51 | 125–192 |
| 174 | Sweden | T3 | 3.57 | 31.53 | 56.34 | 30.48 | 126–188 |
| 175 | Hungary | T3 | 22.48 | 35.96 | 30.85 | 29.76 | 158–178 |
| 176 | Belize | T3 | 39.90 | 17.24 | 31.40 | 29.52 | 151–187 |
| 177 | Cook Islands† | T3 | 17.00 | 1.48 | 69.64 | 29.37 | 128–202 |
| 178 | Austria | T3 | 14.41 | 32.51 | 35.90 | 27.61 | 164–182 |
| 179 | Taiwan (Province of China) | T3 | 18.35 | 41.38 | 21.61 | 27.11 | 155–186 |
| 180 | San Marino | T3 | 49.57 | 5.17 | 25.18 | 26.64 | 146–200 |
| 181 | Palau | T3 | 2.77 | 0.00 | 76.79 | 26.52 | 141–204 |
| 182 | Singapore | T3 | 11.02 | 25.37 | 41.75 | 26.05 | 168–187 |
| 183 | Sao Tome and Principe | T3 | 37.87 | 11.45 | 27.89 | 25.74 | 154–193 |
| 184 | Montenegro | T3 | 39.29 | 17.00 | 20.26 | 25.51 | 160–195 |
| 185 | Serbia | T3 | 31.96 | 40.39 | 3.45 | 25.27 | 141–193 |
| 186 | Slovenia | T3 | 20.69 | 19.46 | 35.41 | 25.18 | 165–186 |
| 187 | Finland | T3 | 19.15 | 29.06 | 25.99 | 24.73 | 174–186 |
| 188 | Australia | T3 | 10.96 | 48.28 | 11.45 | 23.56 | 156–198 |
| 189 | Iceland | T3 | 18.35 | 11.82 | 36.76 | 22.31 | 166–195 |
| 190 | Portugal | T3 | 22.91 | 37.93 | 4.80 | 21.88 | 153–196 |
| 191 | Maldives | T3 | 26.35 | 14.53 | 21.92 | 20.94 | 173–196 |
| 192 | New Zealand† | T3 | 5.97 | 26.60 | 28.02 | 20.20 | 183–196 |
| 193 | Republic of Korea | T3 | 6.53 | 44.58 | 6.90 | 19.33 | 169–201 |
| 194 | Cyprus | T3 | 15.89 | 17.24 | 23.21 | 18.78 | 187–198 |
| 195 | Ireland | T3 | 12.56 | 29.06 | 13.61 | 18.41 | 180–197 |
| 196 | Canada† | T3 | 4.43 | 49.51 | 0.62 | 18.19 | 166–204 |
| 197 | Andorra | T3 | 25.68 | 5.30 | 22.54 | 17.84 | 174–201 |
| 198 | Puerto Rico | T3 | 20.50 | 22.17 | 9.98 | 17.55 | 178–199 |
| 199 | United States Virgin Islands† | T3 | 14.84 | 5.05 | 29.80 | 16.56 | 179–201 |
| 200 | Switzerland† | T3 | 7.76 | 31.28 | 7.88 | 15.64 | 185–201 |
| 201 | Denmark† | T3 | 6.10 | 25.86 | 9.85 | 13.94 | 191–202 |
| 202 | Greenland | T3 | 24.75 | 6.16 | 8.07 | 12.99 | 193–204 |
| 203 | Luxembourg | T3 | 7.27 | 11.58 | 4.80 | 7.88 | 201–204 |
| 204 | Norway | T3 | 2.34 | 17.73 | 3.39 | 7.82 | 199–204 |
